# Supplementary material for: Bacteriophage and Antibiotic Resistance Are Positively Associated across a Phylogenetically Diverse Set of Clinical Pseudomonas aeruginosa Isolates
Source: bioRxiv. 2026 Sep 28:2026.09.27.754834. Preprint. [Version 1] doi: 10.64898/2026.09.27.754834 (PMC13648503; doi:10.64898/2026.09.27.754834)
Supplement: Supplementary file 1 [file NIHPP2026.09.27.754834v1-supplement-1.pdf]

bioRxiv preprint doi: https://doi.org/10.64898/2026.09.27.754834; this version posted September 29, 2026. The copyright holder for this preprint (which was not certified by peer review) is the author/funder, who has granted bioRxiv a license to display the preprint in perpetuity. It is made available under a CC-BY 4.0 International license.

Supplementary Table 1

strain	Luz19	PAML31-1	OMKO1	AMK	AZM	CPM	CAZ	CIP	COL	DOR	IPM	LVX	MEM	TOB
1	0.03322575	0.06615145	0.034703267	1	0	0	0	0	0.5	0	0	0	0	0.5
2	0.406899905	0.14330975	0.868423802	0	1	0	0	0	0.5	0	0	0	0	0
3	0.268204978	0.199540241	0.694667436	1	0	0	0	0	0.5	0	0	0	0	0
4	0.224367874	0.17519317	0.677634695	1	1	1	1	0	0.5	1	0	0	0.5	0
5	0.59316481	0.576949599	0.95998133	1	0	1	1	0.5	0.5	0	0	0	0	1
6	0.292022908	0.089573829	0.656464779	1	1	1	1	0	0.5	1	0	0	1	0
7	0.37524173	0.266297298	0.760057508	0	0	0	0	0	0.5	0	0	0	0	0
8	0.926133757	0.024862455	0.913019283	1	1	1	1	0	0.5	1	0	0	1	0
9	0.931565961	0.634721076	0.967057715	1	1	1	1	1	0.5	1	0	1	1	1
10	0.904847543	0.971932478	0.684887966	1	1	1	1	1	0.5	1	1	1	1	1
11	0.315853607	-0.111989965	0.176068709	0	0	0	0	0	0	0	0	0	0	0
12	0.557465897	0.101633413	0.944153326	0.5	0	0	0	0	0.5	0	0	0	0	0
13	0.908341296	-0.080202084	0.577253352	0.5	0	0	0	0	0.5	0	0	0	0	0
14	0.559267233	0.203601209	0.852512745	0.5	1	0	0	0	0.5	0.5	0	0	0.5	0
15	0.093815828	0.193875391	0.751570195	0.5	1	0	0.5	0	0.5	0	0	0	0	0
16	0.806382819	0.331439876	0.819277069	1	0	0.5	1	0	0.5	0	0	0	0	0
17	0.942353364	0.926143239	0.899983781	0	0	0	0	0	0.5	0	0	0	0	0
18	0.110507403	0.113528651	0.758269702	0	0	0	0	0	0.5	0	0	0	0	0
19	0.764195736	0.996200929	0.837979296	1	1	1	1	1	0.5	1	0	1	1	1
20	0.174686654	0.195070726	0.685939105	0	1	0	0	0	0.5	0.5	0	0	0.5	0
21	0.311085355	0.141793708	0.492081783	0	0	0	0	0	0.5	0	0	0	0	0
22	0.151897108	0.113748982	0.769622243	0	0	0	0	0	0.5	0	0	0	0	0
23	0.229200202	0.249717714	0.840349251	1	0	0.5	1	0	0.5	1	1	0	1	1
24	0.372827635	0.499400004	0.640270817	1	0	0	0	0	0.5	0	1	0	0	0
25	0.450820252	0.994386832	0.928739979	1	0.5	1	0.5	1	0.5	1	1	0.5	1	1
26	0.080546343	0.093779274	0.723865916	0	1	0	0	0	0.5	0	0	0	0	0
27	0.0457542	0.031414063	0.774068377	0	0.5	0	0	0	0.5	0	0	0	0	0

bioRxiv preprint doi: https://doi.org/10.64898/2026.09.27.754834; this version posted September 29, 2026. The copyright holder for this preprint (which was not certified by peer review) is the author/funder, who has granted bioRxiv a license to display the preprint in perpetuity. It is made available under a CC-BY 4.0 International license.

**28**	0.924775077	0.228884211	0.971262267	1	1	1	1	1	0.5	1	0	1	0.5	1
**29**	0.986550408	0.985638416	0.814151272	1	0.5	1	1	0	0	1	1	0	0.5	1
**30**	0.443776628	0.120165346	0.591942351	1	1	1	1	1	0.5	1	1	1	1	1
**31**	0.666245403	0.960790926	0.223943725	1	1	1	1	1	0.5	1	1	1	1	1
**32**	0.968341667	0.865542332	0.867506047	0	1	1	1	0	0.5	0.5	1	0	0	0
**33**	0.263120491	0.399762143	0.279131268	1	1	1	1	0	0.5	1	1	0	1	0.5
**34**	0.50191201	0.452945441	0.920975222	1	1	1	1	0.5	0.5	1	1	0.5	1	1
**35**	0.177210704	-0.116995407	0.89757692	1	1	1	1	0	0.5	1	1	0.5	1	0
**36**	0.796737271	0.178104758	0.613100552	1	1	1	1	0	0.5	1	0	0	1	1
**37**	0.964597011	0.556078628	0.987016969	1	1	1	1	0	0.5	0	0	0	0	0
**38**	0.916986798	-0.086974663	0.025428001	1	1	0.5	0.5	0	0.5	0	0	0	0	0
**39**	0.391814237	0.683598914	0.37508583	1	1	1	1	0	0.5	1	0	0	0.5	0.5
**40**	0.432593005	0.535664875	0.562915686	1	0	0	0	0	0.5	0	0	0	0	1
**41**	0.994990329	0.680086569	0.675633282	1	1	1	1	0	0.5	1	0	0	0.5	1
**42**	0.966251062	0.927353804	0.963920948	1	1	1	1	0	0.5	1	0	0	1	0.5
**43**	0.941954	0.215881383	0.507045798	1	0.5	1	1	0	0.5	0.5	0	0	0	0
**44**	0.862125373	0.079362299	0.078902863	1	1	1	1	0	0.5	1	0	0	1	0
**45**	0.515228066	0.000389522	0.947146537	0	0	0	0	0	0.5	0	0	0	0	0
**46**	0.494019082	0.11379871	0.537610947	0	0	0	0	0	0.5	0	0	0	0	0
**47**	0.506786384	-0.039588922	0.60640882	0	0	0	0	0	0.5	0	0	0	0	0
**48**	0.569385375	-0.232279593	0.719300375	0	0	0	0	0	0.5	0	0	0	0	0
**49**	0.36287104	0.13990727	0.732637606	0	0	0	0	0	0.5	0	0	0	0	0
**50**	0.40002337	0.03758239	0.9831453	0	0	0	0	0	0	0	0	0	0	0
**51**	0.005505534	-0.022218187	0.935821188	1	0	0	0	0	0.5	0	0	0	0	0
**52**	0.43352058	0.213772613	0.838249455	0	0	0	0	0	0.5	0	0	0	0	0
**53**	0.123107078	0.094425763	0.598118963	0	0	0	0	0	0.5	0	0	0	0	0
**54**	0.812321128	0.67260405	0.713498684	0	0	0	0	0	0.5	0	0.5	0	0	0
**55**	0.374314769	0.194272775	0.693668639	0	0	0	0	0	0.5	0	0	0	0	0

bioRxiv preprint doi: https://doi.org/10.64898/2026.09.27.754834; this version posted September 29, 2026. The copyright holder for this preprint (which was not certified by peer review) is the author/funder, who has granted bioRxiv a license to display the preprint in perpetuity. It is made available under a CC-BY 4.0 International license.

Supplemental Table 1 Data matrix displaying suppression index values for each phage against each strain, as well as scaled susceptibility values based on MIC using S/I/R breakpoints adapted from CLSI 2025 M100 S35. S/I/R converted to numerical scale by assigning S=1, I=0.5, R=0 to match directionality of the suppression index used for phage AST. AMK: amikacin; AZM: aztreonam; CPM: cefepime; CAZ: ceftazidime; CIP: ciprofloxacin; COL: colistin; DOR: doripenem; IPM: imipenem; LVX: levofloxacin; MEM: meropenem; TOB: tobramycin confounded by the lineage effect.

bioRxiv preprint doi: https://doi.org/10.64898/2026.09.27.754834; this version posted September 29, 2026. The copyright holder for this preprint (which was not certified by peer review) is the author/funder, who has granted bioRxiv a license to display the preprint in perpetuity. It is made available under a CC-BY 4.0 International license.

Supplementary Table 2

strain	acsA	aroE	guaA	mutL	nuoD	ppsA	trpE	MLST
1								Unknown
2	16	5	30	11	4	31	41	233(Pasteur)
3	6	5	11	7	3	12	19	282(Pasteur)
4	38	11	3	13	1	2	4	235(Pasteur)
5	6	5	6	7	4	6	7	27(Pasteur)
6	38	11	3	13	1	2	4	235(Pasteur)
7	2	4	5	3	1	6	11	357(Pasteur)
8	18	4	5	3	1	17	13	446(Pasteur)
9	6	5	6	7	4	6	7	27(Pasteur)
10	15	5	11	72	4	15	3	2012(Pasteur)
11	13	8	9	3	1	6	9	316(Pasteur)
12	38	11	3	13	1	2	4	235(Pasteur)
13	17	5	5	4	4	4	3	111(Pasteur)
14	16	5	30	11	4	31	41	233(Pasteur)
15	16	5	30	11	4	31	41	233(Pasteur)
16	47	8	7	6	8	11	40	313(Pasteur)
17	18	4	5	3	1	17	13	446(Pasteur)
18								Unknown
19	17	3	5	4	4	4	3	966(Pasteur)
20	16	5	30	11	4	31	41	233(Pasteur)
21	16	5	30	11	4	31	41	233(Pasteur)
22	2	4	5	3	1	6	11	357(Pasteur)
23	16	5	19	3	4	13	7	1600(Pasteur)
24	38	11	3	13	1	2	4	235(Pasteur)
25	17	3	5	4	4	4	3	966(Pasteur)
26								Unknown
27	16	5	30	11	4	31	41	233(Pasteur)

bioRxiv preprint doi: https://doi.org/10.64898/2026.09.27.754834; this version posted September 29, 2026. The copyright holder for this preprint (which was not certified by peer review) is the author/funder, who has granted bioRxiv a license to display the preprint in perpetuity. It is made available under a CC-BY 4.0 International license.

**28**	11	5	1	7	9	4	7	17(Pasteur)
**29**	36	27	28	3	4	13	7	179(Pasteur)
**30**	47	8	7	6	8	11	40	313(Pasteur)
**31**	15	5	36	11	27	4	2	360(Pasteur)
**32**	17	22	5	3	1	14	3	389(Pasteur)
**33**	4	4	16	12	1	6	3	253(Pasteur)
**34**	6	28	4	3	3	4	7	252(Pasteur)
**35**	47	8	7	6	8	11	40	313(Pasteur)
**36**								Unknown
**37**	28	22	5	3	3	14	19	175(Pasteur)
**38**	17	3	5	4	4	4	3	966(Pasteur)
**39**	11	5	1	7	9	4	7	17(Pasteur)
**40**	6	28	4	3	3	4	7	252(Pasteur)
**41**	17	3	5	4	4	4	3	966(Pasteur)
**42**	16	5	19	3	4	13	7	1600(Pasteur)
**43**	17	3	5	4	4	4	3	966(Pasteur)
**44**	17	3	5	4	4	4	3	966(Pasteur)
**45**	13	8	9	3	1	17	15	309(Pasteur)
**46**	13	8	9	3	1	17	15	309(Pasteur)
**47**	13	8	9	3	1	17	15	309(Pasteur)
**48**	13	8	9	3	1	17	15	309(Pasteur)
**49**	13	8	9	3	1	17	15	309(Pasteur)
**50**	13	8	9	3	1	17	241	2775(Pasteur)
**51**	13	8	9	3	1	17	15	309(Pasteur)
**52**	13	8	9	3	1	17	15	309(Pasteur)
**53**	13	8	9	3	1	17	15	309(Pasteur)
**54**	6	5	6	7	4	6	7	27(Pasteur)
**55**	13	8	9	3	1	17	15	309(Pasteur)

bioRxiv preprint doi: https://doi.org/10.64898/2026.09.27.754834; this version posted September 29, 2026. The copyright holder for this preprint (which was not certified by peer review) is the author/funder, who has granted bioRxiv a license to display the preprint in perpetuity. It is made available under a CC-BY 4.0 International license.

Supplemental Table 2. MLST allele profiles for each strain, derived from the Pasteur MLST scheme.

bioRxiv preprint doi: https://doi.org/10.64898/2026.09.27.754834; this version posted September 29, 2026. The copyright holder for this preprint (which was not certified by peer review) is the author/funder, who has granted bioRxiv a license to display the preprint in perpetuity. It is made available under a CC-BY 4.0 International license.

Supplementary Table 4

Type	Trait	N	N_unique_values	Lambda	CI_lower	CI_upper	LogLik_full	LogLik_lambda0	LR_statistic	P_value	Q_value_BH	Significant_raw_p05	Significant_FDR_q05
**Phage**	Luz19	55	55	0.506148821444281	0.185	0.755	-7.505347280416 3	-13.153246073819 5	11.2957975868064	0.000776827224694518	0.0021751162291446 5	TRUE	TRUE
**Phage**	PAML31.1	55	55	0.458751267545214	0.115	0.745	-14.326101571336 7	-18.327601794445	8.00300044621665	0.0046699902461350 1	0.0093399804922700 1	TRUE	TRUE
**Phage**	OMKO1	55	55	0.000545076806112702	0	0.33	-0.565063825576864	-0.565063825576864	0	1	1	FALSE	FALSE

Supplemental Table 4 Full statistics for phage Pagel's Lambda results

bioRxiv preprint doi: https://doi.org/10.64898/2026.09.27.754834; this version posted September 29, 2026. The copyright holder for this preprint (which was not certified by peer review) is the author/funder, who has granted bioRxiv a license to display the preprint in perpetuity. It is made available under a CC-BY 4.0 International license.

Supplementary Table 5

Type	Trait	N	N_unique_values	Lambda	CI_lower	CI_upper	LogLik_full	LogLik_lambda0	LR_statistic	P_value	Q_value_BH	Significant_raw_p05	Significant_FDR_q05
**Antibiotic**	Amikacin	51	3	0.583623655815233	0.291073117705318	1	-36.1318771109213	-48.9478919980713	25.6320297743	4.13124810870668E-07	2.75057678170283E-06	TRUE	TRUE
**Antibiotic**	Aztreonam	51	3	0.341361215222115	0.0844289567164309	1	-46.2180832602207	-54.1415923878854	15.8470182553293	6.86746615639619E-05	0.000240361315473867	TRUE	TRUE
**Antibiotic**	Cefepime	51	3	0.548464101550168	0.264183744512241	1	-40.2512721341341	-52.7245631571829	24.9465820460976	5.89409310364892E-07	2.75057678170283E-06	TRUE	TRUE
**Antibiotic**	Ceftazidime	51	3	0.536635784670613	0.253904540043245	1	-40.3447394303149	-53.5353784486961	26.3812780367625	2.8024371717551E-07	2.75057678170283E-06	TRUE	TRUE
**Antibiotic**	Ciprofloxacin	51	3	2.09186620861407E-15	6.38673802494909E-16	6.85154803226222E-15	-30.0042867300506	-30.0043739515862	0.000174443071252028	0.989462094560771	1	FALSE	FALSE
**Antibiotic**	Colistin	51	2	0.999999998591375	NA	NA	-10.4030903574361	-11.4096182233746	2.01305573187706	0.155950931016245	0.242590337136381	FALSE	FALSE
**Antibiotic**	Doripenem	51	3	0.603108440584871	0.305673930700863	1	-47.8637556719734	-51.0477095615417	6.36790777913673	0.0116202520776147	0.0203354411358256	TRUE	TRUE
**Antibiotic**	Imipenem	51	3	0.54031073293618	0.35891983741298	0.81337295322054	-35.711753002239	-36.1429892126257	0.862472420773344	0.353047725948238	0.460013765440368	FALSE	FALSE
**Antibiotic**	Levofloxacin	51	3	2.08091284000505E-93	9.0065161337822E-94	4.80785043115164E-93	-30.0042867300497	-30.0043671622186	0.000160864337857447	0.989880517453254	1	FALSE	FALSE
**Antibiotic**	Meropenem	51	3	0.427429926201871	NA	NA	-48.6917246584325	-49.1081675721485	0.832885827432136	0.361439387131718	0.460013765440368	FALSE	FALSE
**Antibiotic**	Tobramycin	51	3	0.677171643675166	0.159267620696128	1	-39.5261845462835	-42.8142259166835	6.57608274080006	0.0103358109451815	0.0203354411358256	TRUE	TRUE

Supplemental Table 5 Full statistics for antibiotic Pagel's Lambda results

bioRxiv preprint doi: https://doi.org/10.64898/2026.09.27.754834; this version posted September 29, 2026. The copyright holder for this preprint (which was not certified by peer review) is the author/funder, who has granted bioRxiv a license to display the preprint in perpetuity. It is made available under a CC-BY 4.0 International license.

Supplemental Table 6

Antimicrobial 1	Antimicrobial 2	Kendall Tau	P Value
Luz19	PAML31.1	0.309165934351616	0.000964767846046001
Luz19	OMKO1	0.114274037489596	0.222475959443205
Luz19	Amikacin	0.217317895472959	0.0203381738672905
Luz19	Aztreonam	0.199465009672516	0.0332165914958712
Luz19	Cefepime	0.40472762873157	1.55460763325196E-05
Luz19	Ceftazidime	0.376137413806579	5.92995429048305E-05
Luz19	Ciprofloxacin	0.202083314735213	0.0309738799553664
Luz19	Colistin	-0.0479002093303253	0.609088687786896
Luz19	Doripenem	0.242820765365502	0.00953326030752416
Luz19	Imipenem	0.0491086764496132	0.600086501503812
Luz19	Levofloxacin	0.143041403789618	0.126739524762672
Luz19	Meropenem	0.159691964841854	0.088223325997386
Luz19	Tobramycin	0.21906842319661	0.0193489804835644
PAML31.1	OMKO1	0.143816192295581	0.12469593203811
PAML31.1	Amikacin	0.211705706062045	0.0238128629199807
PAML31.1	Aztreonam	0.128355444117926	0.170592794721401
PAML31.1	Cefepime	0.318005819509644	0.000686342071815776
PAML31.1	Ceftazidime	0.290683590822869	0.00191381984191577
PAML31.1	Ciprofloxacin	0.321063695436567	0.000608890369593415
PAML31.1	Colistin	0.0504771189570708	0.589965939224602
PAML31.1	Doripenem	0.282525040940641	0.00255972218837948
PAML31.1	Imipenem	0.275102018879302	0.00331464229965603
PAML31.1	Levofloxacin	0.199678298273253	0.0330288393170906
PAML31.1	Meropenem	0.219779320613198	0.0189594275274181
PAML31.1	Tobramycin	0.44859811632647	1.67481375608889E-06
OMKO1	Amikacin	-0.0237524575605046	0.799822123178491

bioRxiv preprint doi: https://doi.org/10.64898/2026.09.27.754834; this version posted September 29, 2026. The copyright holder for this preprint (which was not certified by peer review) is the author/funder, who has granted bioRxiv a license to display the preprint in perpetuity. It is made available under a CC-BY 4.0 International license.

OMKO1	Aztreonam	0.0225092679631529	0.810093641581864
OMKO1	Cefepime	0.0794038496407648	0.396603734387335
OMKO1	Ceftazidime	0.0753732234424847	0.421008928808586
OMKO1	Ciprofloxacin	0.175900767440951	0.0603965490487416
OMKO1	Colistin	-0.03299275200676	0.724669398929661
OMKO1	Doripenem	0.0145971321084693	0.876161841892514
OMKO1	Imipenem	-0.00701478250049314	0.940303299226186
OMKO1	Levofloxacin	0.154236504322655	0.0996395789327826
OMKO1	Meropenem	0.0265384807583467	0.776931210292703
OMKO1	Tobramycin	0.0301707676602921	0.747378351055097
Amikacin	Aztreonam	0.335392989012174	0.000342805868377063
Amikacin	Cefepime	0.664907772981135	1.26167808563825E-12
Amikacin	Ceftazidime	0.676081501414128	5.28607896372736E-13
Amikacin	Ciprofloxacin	0.301405748558831	0.00129196766275523
Amikacin	Colistin	0.0867186029869962	0.354553310812364
Amikacin	Doripenem	0.562237662385647	1.94474150764206E-09
Amikacin	Imipenem	0.197004688755137	0.0354492045889953
Amikacin	Levofloxacin	0.281861749948314	0.00262014651328336
Amikacin	Meropenem	0.536784610639398	1.00061911309326E-08
Amikacin	Tobramycin	0.56588980654397	1.52833686890107E-09
Aztreonam	Cefepime	0.616833702394945	4.54228271749576E-11
Aztreonam	Ceftazidime	0.604442872707195	1.09706354511814E-10
Aztreonam	Ciprofloxacin	0.237558041993191	0.0112084975599151
Aztreonam	Colistin	0.130132943390392	0.164748285221523
Aztreonam	Doripenem	0.656674234953913	2.37390331282182E-12
Aztreonam	Imipenem	0.109165894206356	0.243842120980373
Aztreonam	Levofloxacin	0.330561335889942	0.000417104358314489
Aztreonam	Meropenem	0.607227137741822	9.01214129389772E-11
Aztreonam	Tobramycin	0.267051415680937	0.00435824039611277

bioRxiv preprint doi: https://doi.org/10.64898/2026.09.27.754834; this version posted September 29, 2026. The copyright holder for this preprint (which was not certified by peer review) is the author/funder, who has granted bioRxiv a license to display the preprint in perpetuity. It is made available under a CC-BY 4.0 International license.

Cefepime	Ceftazidime	0.926430130435923	4.58124125372512E-23
Cefepime	Ciprofloxacin	0.433835208189132	3.62932945232018E-06
Cefepime	Colistin	0.0238707849774563	0.798846246949169
Cefepime	Doripenem	0.784858981695852	5.33679719549428E-17
Cefepime	Imipenem	0.316893492699464	0.000716718467994659
Cefepime	Levofloxacin	0.420224424880861	7.2488840724223E-06
Cefepime	Meropenem	0.680721312660851	3.6682960290962E-13
Cefepime	Tobramycin	0.553521204739291	3.43562414690904E-09
Ceftazidime	Ciprofloxacin	0.357128831042333	0.000137485602841555
Ceftazidime	Colistin	0.0299282677379648	0.749340381293581
Ceftazidime	Doripenem	0.747344514788569	1.48090291333006E-15
Ceftazidime	Imipenem	0.28800103180418	0.00210748672986299
Ceftazidime	Levofloxacin	0.356131255256431	0.000143533600883017
Ceftazidime	Meropenem	0.646180656781688	5.25478243668303E-12
Ceftazidime	Tobramycin	0.525755558386889	1.98987114411017E-08
Ciprofloxacin	Colistin	0.0810413043354187	0.38693803235154
Ciprofloxacin	Doripenem	0.392989944602977	2.72275361859152E-05
Ciprofloxacin	Imipenem	0.310534596722036	0.000915723064104513
Ciprofloxacin	Levofloxacin	0.868653411984192	1.79966125365605E-20
Ciprofloxacin	Meropenem	0.429176244670938	4.60958173332059E-06
Ciprofloxacin	Tobramycin	0.650330810278615	3.84337116111E-12
Colistin	Doripenem	0.00683234445522147	0.941853082220288
Colistin	Imipenem	-0.102665401266671	0.273061761686651
Colistin	Levofloxacin	0.0769095305730228	0.411604430559186
Colistin	Meropenem	0.0466635648256965	0.618362452982349
Colistin	Tobramycin	-0.0328449247749715	0.725853195403791
Doripenem	Imipenem	0.383939903438346	4.15155936583245E-05
Doripenem	Levofloxacin	0.477651100446476	3.40868108408526E-07
Doripenem	Meropenem	0.900579918988044	6.94846974030866E-22

bioRxiv preprint doi: https://doi.org/10.64898/2026.09.27.754834; this version posted September 29, 2026. The copyright holder for this preprint (which was not certified by peer review) is the author/funder, who has granted bioRxiv a license to display the preprint in perpetuity. It is made available under a CC-BY 4.0 International license.

Doripenem	Tobramycin	0.577643785254357	6.9672725017119E-10
Imipenem	Levofloxacin	0.404957468913703	1.53741004797603E-05
Imipenem	Meropenem	0.374531484500686	6.37623351680851E-05
Imipenem	Tobramycin	0.3696154669445	7.94829693938726E-05
Levofloxacin	Meropenem	0.51622891501109	3.56446751802703E-08
Levofloxacin	Tobramycin	0.546467662843488	5.41145883541978E-09
Meropenem	Tobramycin	0.569571133922189	1.1969825404119E-09

Supplemental Table 6 Kendall’s Tau correlation values by pairing, with P values, phylogenetically controlled (partial Kendall’s Tau)

bioRxiv preprint doi: https://doi.org/10.64898/2026.09.27.754834; this version posted September 29, 2026. The copyright holder for this preprint (which was not certified by peer review) is the author/funder, who has granted bioRxiv a license to display the preprint in perpetuity. It is made available under a CC-BY 4.0 International license.

Supplemental Table 7

Antimicrobial 1	Antimicrobial 2	Kendall_Tau	P_Value
Luz19	PAML31.1	0.314478114478114	0.000698403200632007
Luz19	OMKO1	0.113804713804714	0.219874359864308
Luz19	Amikacin	0.222846300488227	0.0410005705675774
Luz19	Aztreonam	0.207789546502013	0.056667800520718
Luz19	Cefepime	0.400719596884288	0.000257843311934169
Luz19	Ceftazidime	0.371551330492151	0.00070288527649898
Luz19	Ciprofloxacin	0.210728934081251	0.0567382213751934
Luz19	Colistin	-0.0415531995559314	0.710917113388619
Luz19	Doripenem	0.24950672135471	0.0221522725024653
Luz19	Imipenem	0.0644326986282696	0.562349755518756
Luz19	Levofloxacin	0.154816264713781	0.159990120498132
Luz19	Meropenem	0.170136723721774	0.114713439992402
Luz19	Tobramycin	0.225987299157516	0.0385118098256637
PAML31.1	OMKO1	0.139393939393939	0.132910025313192
PAML31.1	Amikacin	0.278557875610283	0.0106369361110816
PAML31.1	Aztreonam	0.19535769329249	0.073158867321226
PAML31.1	Cefepime	0.366346358325365	0.000835144661936959
PAML31.1	Ceftazidime	0.344496621961169	0.00167958949778114
PAML31.1	Ciprofloxacin	0.361249601282144	0.00108975690758485
PAML31.1	Colistin	0.0706404392450834	0.528654073421974
PAML31.1	Doripenem	0.329059589033023	0.00255143924198161
PAML31.1	Imipenem	0.319902696698251	0.00402180925711214
PAML31.1	Levofloxacin	0.254697725819447	0.0207984125146413
PAML31.1	Meropenem	0.267357708705644	0.0131860827519086
PAML31.1	Tobramycin	0.469358236711764	1.72413981053547E-05
OMKO1	Amikacin	-0.0233629185995721	0.830359558245747

bioRxiv preprint doi: https://doi.org/10.64898/2026.09.27.754834; this version posted September 29, 2026. The copyright holder for this preprint (which was not certified by peer review) is the author/funder, who has granted bioRxiv a license to display the preprint in perpetuity. It is made available under a CC-BY 4.0 International license.

OMKO1	Aztreonam	0.019535769329249	0.857793357869626
OMKO1	Cefepime	0.073269271665073	0.504021744094166
OMKO1	Ceftazidime	0.0685385949451541	0.531935544978916
OMKO1	Ciprofloxacin	0.168081411707664	0.128581072118439
OMKO1	Colistin	-0.0332425596447451	0.766848475677745
OMKO1	Doripenem	0.0126561380397317	0.907616778096202
OMKO1	Imipenem	-0.00791278755084013	0.943279295948559
OMKO1	Levofloxacin	0.144828118603215	0.188693849701051
OMKO1	Meropenem	0.0243052462459677	0.821717357687815
OMKO1	Tobramycin	0.02897273066122	0.790775855103335
Amikacin	Aztreonam	0.432494813542581	0.000769741703292617
Amikacin	Cefepime	0.709731993877846	4.08174025794427E-08
Amikacin	Ceftazidime	0.724433120259706	2.1296855450943E-08
Amikacin	Ciprofloxacin	0.37659765228037	0.00389694542228402
Amikacin	Colistin	0.119212866575011	0.367516576388719
Amikacin	Doripenem	0.612797635822239	1.89744266105448E-06
Amikacin	Imipenem	0.289609561315821	0.0272990902331651
Amikacin	Levofloxacin	0.374850089940042	0.00392541888780205
Amikacin	Meropenem	0.584985934561171	4.2388676577588E-06
Amikacin	Tobramycin	0.579910389719674	6.72101566167934E-06
Aztreonam	Cefepime	0.66320217011254	2.91808241406712E-07
Aztreonam	Ceftazidime	0.65643990505311	3.84382209952561E-07
Aztreonam	Ciprofloxacin	0.310961791316029	0.0171324649729665
Aztreonam	Colistin	0.156164969657582	0.237714895159303
Aztreonam	Doripenem	0.692601760603008	7.21816387863572E-08
Aztreonam	Imipenem	0.199742534108086	0.127848259455171
Aztreonam	Levofloxacin	0.406655026217331	0.00175110303146331
Aztreonam	Meropenem	0.644570436236096	4.00130658443757E-07
Aztreonam	Tobramycin	0.308189495463132	0.0166992426112692

bioRxiv preprint doi: https://doi.org/10.64898/2026.09.27.754834; this version posted September 29, 2026. The copyright holder for this preprint (which was not certified by peer review) is the author/funder, who has granted bioRxiv a license to display the preprint in perpetuity. It is made available under a CC-BY 4.0 International license.

Cefepime	Ceftazidime	0.935195082093422	6.4770194351193E-13
Cefepime	Ciprofloxacin	0.485255744879414	0.000217076542940123
Cefepime	Colistin	0.0558171655583031	0.674815025301252
Cefepime	Doripenem	0.806314659856016	4.56726464889063E-10
Cefepime	Imipenem	0.382644359255739	0.0037333242325047
Cefepime	Levofloxacin	0.483003965931659	0.000219595166655178
Cefepime	Meropenem	0.709802740423545	2.86750882383915E-08
Cefepime	Tobramycin	0.572097530461051	1.00206017741719E-05
Ceftazidime	Ciprofloxacin	0.419955192835313	0.00137095157341879
Ceftazidime	Colistin	0.063995637315692	0.630489510764121
Ceftazidime	Doripenem	0.773610015920683	2.22231344831493E-09
Ceftazidime	Imipenem	0.361807383143103	0.00610599078593126
Ceftazidime	Levofloxacin	0.431382640224006	0.000965114929638706
Ceftazidime	Meropenem	0.679928778958982	1.06100161799241E-07
Ceftazidime	Tobramycin	0.545793907871637	2.5111464881584E-05
Ciprofloxacin	Colistin	0.104491167214653	0.436230715856846
Ciprofloxacin	Doripenem	0.442863877435301	0.000689080051526708
Ciprofloxacin	Imipenem	0.364266230009518	0.00620814373720085
Ciprofloxacin	Levofloxacin	0.879079276192867	2.60762685604644E-11
Ciprofloxacin	Meropenem	0.472853613174189	0.000247550464433982
Ciprofloxacin	Tobramycin	0.663797913347633	3.77052560712402E-07
Colistin	Doripenem	0.0362591356639103	0.784043898768489
Colistin	Imipenem	-0.0697529193733527	0.605287825353237
Colistin	Levofloxacin	0.10400628679223	0.436591057655158
Colistin	Meropenem	0.0722121934473044	0.580958593199882
Colistin	Tobramycin	-0.0148983820282184	0.910463902323082
Doripenem	Imipenem	0.437042401893306	0.000866992915340557
Doripenem	Levofloxacin	0.527964928777866	4.87177191559515E-05
Doripenem	Meropenem	0.908769458092314	9.05409154723235E-13

bioRxiv preprint doi: https://doi.org/10.64898/2026.09.27.754834; this version posted September 29, 2026. The copyright holder for this preprint (which was not certified by peer review) is the author/funder, who has granted bioRxiv a license to display the preprint in perpetuity. It is made available under a CC-BY 4.0 International license.

Doripenem	Tobramycin	0.59508702457554	3.84406246535731E-06
Imipenem	Levofloxacin	0.458983352530419	0.000537179968378109
Imipenem	Meropenem	0.424620053205624	0.00106602897886769
Imipenem	Tobramycin	0.398805556562867	0.00240527953433005
Levofloxacin	Meropenem	0.559116645339574	1.36088233550079E-05
Levofloxacin	Tobramycin	0.565817806081494	1.37971370770347E-05
Meropenem	Tobramycin	0.587411285410158	3.99436198855566E-06

Supplemental Table 7 Kendall’s Tau correlation values by pairing, with P values, not phylogenetically controlled.

bioRxiv preprint doi: https://doi.org/10.64898/2026.09.27.754834; this version posted September 29, 2026. The copyright holder for this preprint (which was not certified by peer review) is the author/funder, who has granted bioRxiv a license to display the preprint in perpetuity. It is made available under a CC-BY 4.0 International license.

Supplemental Table 8

Test	N	Estimate	CI_lower_95	CI_upper_95	P_value	CI_method
**Simple Spearman (no phylogenetic correction)**	55	0.409426273013103	0.165812177696411	0.615950658431408	0.00190962693061229	Nonparametric bootstrap, 2000 resamples (percentile method)
**Partial Spearman (controlling for phylogeny via PC1 only, ~49% of variance)**	51	0.358279127546917	0.0533095839703745	0.595592115966338	0.0106257727100883	Nonparametric bootstrap, 2000 resamples (percentile method); 2000/2000 resamples valid
**Partial Mantel (controlling for FULL phylogenetic distance matrix)**	51	0.181896068983736	0.0596546457904211	0.418087983017112	0.001	P-value: Mantel permutation test (999 perms). CI: bootstrap over strains, 2000 resamples
**PGLS slope with Pagel's lambda (full-tree covariance)**	51	0.218129213253479	0.00645451706353828	0.429803909443419	0.043661413140997	Wald-type profile CI from PGLS fit (nlme::intervals); P-value from GLS t-test

Supplemental Table 8 Overall Antibiotic and Phage resistance correlation values via four different tests, varying in their approach to phylogenetic correction. Confidence intervals, exact P values, and method of calculating confidence intervals included.

bioRxiv preprint doi: https://doi.org/10.64898/2026.09.27.754834; this version posted September 29, 2026. The copyright holder for this preprint (which was not certified by peer review) is the author/funder, who has granted bioRxiv a license to display the preprint in perpetuity. It is made available under a CC-BY 4.0 International license.

Supplemental Table 9

Pairing	Gene	FC	OddsRatio	CIL	CIU	Pval	L W	PR W	P R	PS W	PS	Category	FDR
**Luz19 + Amikacin**	aac(3)-Id	6.5	NA	NA	NA	NA	1	4	8	1	13	Aminoglycoside Genes	NA
**Luz19 + Amikacin**	aac(6')-29B	0	NA	NA	NA	NA	1	0	8	1	13	Aminoglycoside Genes	NA
**Luz19 + Amikacin**	aac(6')-33	0.18	0	0	34.66	0.2188	3	1	8	9	13	Aminoglycoside Genes	1
**Luz19 + Amikacin**	aac(6')-Ib-G	0	0	0	Inf	0.9978	2	0	8	6	13	Aminoglycoside Genes	1
**Luz19 + Amikacin**	aac(6')-IB3	NA	NA	NA	NA	NA	1	1	8	0	13	Aminoglycoside Genes	NA
**Luz19 + Amikacin**	aac(6')-IIa	NA	NA	NA	NA	NA	1	1	8	0	13	Aminoglycoside Genes	NA
**Luz19 + Amikacin**	aac(6')-IIc	NA	NA	NA	NA	NA	0	0	8	0	13	Aminoglycoside Genes	NA
**Luz19 + Amikacin**	aac(6')-Il	5.69	9.883E+52	0	Inf	0.9973	3	7	8	2	13	Aminoglycoside Genes	1
**Luz19 + Amikacin**	aac(6')-SET	NA	NA	NA	NA	NA	0	0	8	0	13	Aminoglycoside Genes	NA
**Luz19 + Amikacin**	aadA1	0.54	0.57	0.01	22.88	0.765	3	3	8	9	13	Aminoglycoside Genes	1
**Luz19 + Amikacin**	aadA2	6.5	NA	NA	NA	NA	1	4	8	1	13	Aminoglycoside Genes	NA
**Luz19 + Amikacin**	aadA6	0	NA	NA	NA	NA	1	0	8	1	13	Aminoglycoside Genes	NA
**Luz19 + Amikacin**	aadA7	NA	NA	NA	NA	NA	0	0	8	0	13	Aminoglycoside Genes	NA
**Luz19 + Amikacin**	ant(2'')-la	4.88	1.03E+69	0	Inf	0.998	2	3	8	1	13	Aminoglycoside Genes	1
**Luz19 + Amikacin**	aph(3')-Ia	NA	NA	NA	NA	NA	1	1	8	0	13	Aminoglycoside Genes	NA
**Luz19 + Amikacin**	aph(3')-Ib	0	NA	NA	NA	NA	1	0	8	1	13	Aminoglycoside Genes	NA
**Luz19 + Amikacin**	aph(3')-IIb	1.08	1.459E+7	0	Inf	0.998	8	8	8	12	13	Aminoglycoside Genes	1
**Luz19 + Amikacin**	aph(6)-Id	NA	NA	NA	NA	NA	1	1	8	0	13	Aminoglycoside Genes	NA
**Luz19 + Amikacin**	bcr1	1	NA	NA	NA	NA	8	8	8	13	13	Phenicols Bicyclomycins Genes	NA
**Luz19 + Amikacin**	catB7	0	0	0	Inf	0.9979	2	0	8	2	13	Phenicols Bicyclomycins Genes	1
**Luz19 + Amikacin**	cmlA5	NA	NA	NA	NA	NA	1	1	8	0	13	Phenicols Bicyclomycins Genes	NA
**Luz19 + Amikacin**	cmlA6	6.5	NA	NA	NA	NA	1	4	8	1	13	Phenicols Bicyclomycins Genes	NA
**Luz19 + Amikacin**	cmlB	NA	NA	NA	NA	NA	0	0	8	0	13	Phenicols Bicyclomycins Genes	NA
**Luz19 + Amikacin**	crpP	0	NA	NA	NA	NA	1	0	8	1	13	Quinolone Genes	NA
**Luz19 + Amikacin**	dfrB2	3.25	NA	NA	NA	NA	1	2	8	1	13	Trimethoprim Genes	NA
**Luz19 + Amikacin**	dfrB5	8.12	208.72	0.03	1.65E+6	0.2434	2	5	8	1	13	Trimethoprim Genes	1
**Luz19 + Amikacin**	floR2	11.4	1.104E+64	0	Inf	0.9986	3	7	8	1	13	Phenicols Bicyclomycins Genes	1
**Luz19 + Amikacin**	fosA	1.08	1.661E+7	0	Inf	0.998	7	8	8	12	13	Fosfomycin Genes	1
**Luz19 + Amikacin**	GES-1	NA	NA	NA	NA	NA	1	1	8	0	13	Beta lactam Genes	NA
**Luz19 + Amikacin**	GES-19	0.23	0.14	0	5.4	0.2905	2	1	8	7	13	Beta lactam Genes	1
**Luz19 + Amikacin**	GES-20	0.2	0.11	0	2.55	0.1693	3	1	8	8	13	Beta lactam Genes	1
**Luz19 + Amikacin**	GES-26	0	0	0	Inf	0.9979	2	0	8	2	13	Beta lactam Genes	1
**Luz19 + Amikacin**	IMP-1	NA	NA	NA	NA	NA	0	0	8	0	13	Beta lactam Genes	NA

bioRxiv preprint doi: https://doi.org/10.64898/2026.09.27.754834; this version posted September 29, 2026. The copyright holder for this preprint (which was not certified by peer review) is the author/funder, who has granted bioRxiv a license to display the preprint in perpetuity. It is made available under a CC-BY 4.0 International license.

**Luz19 + Amikacin**	KPC-5	NA	NA	NA	NA	NA	0	0	8	0	13	Beta lactam Genes	NA
**Luz19 + Amikacin**	mexA	1	NA	NA	NA	NA	8	8	8	13	13	Efflux pumps Other Genes	NA
**Luz19 + Amikacin**	mexE	1	NA	NA	NA	NA	8	8	8	13	13	Efflux pumps Other Genes	NA
**Luz19 + Amikacin**	mexX	0.74	0	0	215.11	0.2686	5	5	8	11	13	Efflux pumps Other Genes	1
**Luz19 + Amikacin**	NDM-1	NA	NA	NA	NA	NA	1	2	8	0	13	Beta lactam Genes	NA
**Luz19 + Amikacin**	OXA-10	4.88	1.03E+69	0	Inf	0.998	2	3	8	1	13	Beta lactam Genes	1
**Luz19 + Amikacin**	OXA-101	NA	NA	NA	NA	NA	0	0	8	0	13	Beta lactam Genes	NA
**Luz19 + Amikacin**	OXA-2	0.2	0	0	113.93	0.2229	2	1	8	8	13	Beta lactam Genes	1
**Luz19 + Amikacin**	OXA-395	NA	NA	NA	NA	NA	1	1	8	0	13	Beta lactam Genes	NA
**Luz19 + Amikacin**	OXA-396	NA	NA	NA	NA	NA	0	0	8	0	13	Beta lactam Genes	NA
**Luz19 + Amikacin**	OXA-4	6.5	NA	NA	NA	NA	1	4	8	1	13	Beta lactam Genes	NA
**Luz19 + Amikacin**	OXA-486	6.5	NA	NA	NA	NA	1	4	8	1	13	Beta lactam Genes	NA
**Luz19 + Amikacin**	OXA-488	NA	NA	NA	NA	NA	0	0	8	0	13	Beta lactam Genes	NA
**Luz19 + Amikacin**	OXA-494	0	NA	NA	NA	NA	1	0	8	1	13	Beta lactam Genes	NA
**Luz19 + Amikacin**	OXA-50	0	NA	NA	NA	NA	1	0	8	1	13	Beta lactam Genes	NA
**Luz19 + Amikacin**	OXA-846	0.54	0.57	0.01	22.88	0.765	3	3	8	9	13	Beta lactam Genes	1
**Luz19 + Amikacin**	OXA-848	0	NA	NA	NA	NA	1	0	8	1	13	Beta lactam Genes	NA
**Luz19 + Amikacin**	OXA-9	NA	NA	NA	NA	NA	0	0	8	0	13	Beta lactam Genes	NA
**Luz19 + Amikacin**	OXA-914	NA	NA	NA	NA	NA	0	0	8	0	13	Beta lactam Genes	NA
**Luz19 + Amikacin**	OXA-937	NA	NA	NA	NA	NA	0	0	8	0	13	Beta lactam Genes	NA
**Luz19 + Amikacin**	PAO	NA	NA	NA	NA	NA	0	0	8	0	13	Beta lactam Genes	NA
**Luz19 + Amikacin**	PDC-103	NA	NA	NA	NA	NA	0	0	8	0	13	Beta lactam Genes	NA
**Luz19 + Amikacin**	PDC-11	3.25	NA	NA	NA	NA	1	2	8	1	13	Beta lactam Genes	NA
**Luz19 + Amikacin**	PDC-15	0	NA	NA	NA	NA	1	0	8	1	13	Beta lactam Genes	NA
**Luz19 + Amikacin**	PDC-16	0	NA	NA	NA	NA	1	0	8	1	13	Beta lactam Genes	NA
**Luz19 + Amikacin**	PDC-173	0	NA	NA	NA	NA	1	0	8	1	13	Beta lactam Genes	NA
**Luz19 + Amikacin**	PDC-19A	0.2	0	0	113.93	0.2229	2	1	8	8	13	Beta lactam Genes	1
**Luz19 + Amikacin**	PDC-3	6.5	NA	NA	NA	NA	1	4	8	1	13	Beta lactam Genes	NA
**Luz19 + Amikacin**	PDC-34	NA	NA	NA	NA	NA	0	0	8	0	13	Beta lactam Genes	NA
**Luz19 + Amikacin**	PDC-35	NA	NA	NA	NA	NA	0	0	8	0	13	Beta lactam Genes	NA
**Luz19 + Amikacin**	PDC-36	NA	NA	NA	NA	NA	1	1	8	0	13	Beta lactam Genes	NA
**Luz19 + Amikacin**	PDC-37	NA	NA	NA	NA	NA	0	0	8	0	13	Beta lactam Genes	NA
**Luz19 + Amikacin**	PDC-407	NA	NA	NA	NA	NA	0	0	8	0	13	Beta lactam Genes	NA
**Luz19 + Amikacin**	PDC-5	NA	NA	NA	NA	NA	0	0	8	0	13	Beta lactam Genes	NA
**Luz19 + Amikacin**	PDC-8	NA	NA	NA	NA	NA	0	0	8	0	13	Beta lactam Genes	NA
**Luz19 + Amikacin**	PME-1	NA	NA	NA	NA	NA	1	2	8	0	13	Beta lactam Genes	NA

bioRxiv preprint doi: https://doi.org/10.64898/2026.09.27.754834; this version posted September 29, 2026. The copyright holder for this preprint (which was not certified by peer review) is the author/funder, who has granted bioRxiv a license to display the preprint in perpetuity. It is made available under a CC-BY 4.0 International license.

**Luz19 + Amikacin**	qacEdelta1	0.18	0	0	34.66	0.2188	3	1	8	9	13	Efflux pumps Other Genes	1
**Luz19 + Amikacin**	qacG2	0	NA	NA	NA	NA	1	0	8	1	13	Efflux pumps Other Genes	NA
**Luz19 + Amikacin**	rmtD2	NA	NA	NA	NA	NA	1	2	8	0	13	Aminoglycoside Genes	NA
**Luz19 + Amikacin**	strA	NA	NA	NA	NA	NA	1	1	8	0	13	Aminoglycoside Genes	NA
**Luz19 + Amikacin**	sul1	1.18	3.575E7	0	Inf	0.9982	7	8	8	11	13	Sulfonamides Genes	1
**Luz19 + Amikacin**	tet(C)	NA	NA	NA	NA	NA	0	0	8	0	13	Tetracyclines Genes	NA
**Luz19 + Amikacin**	tet(G)	11.4	1.104E+64	0	Inf	0.9986	3	7	8	1	13	Tetracyclines Genes	1
**Luz19 + Amikacin**	tet(R)	3.25	NA	NA	NA	NA	1	2	8	1	13	Tetracyclines Genes	NA
**Luz19 + Amikacin**	-VEB 9.00	3.25	NA	NA	NA	NA	1	2	8	1	13	Beta lactam Genes	NA
**Luz19 + Amikacin**	VIM-11	NA	NA	NA	NA	NA	1	1	8	0	13	Beta lactam Genes	NA
**Luz19 + Amikacin**	VIM-2	3.25	1.22	0.03	53.71	0.9172	2	4	8	2	13	Beta lactam Genes	1
**Luz19 + Aztreonam**	aac(3)-Id	NA	NA	NA	NA	NA	1	1	9	0	17	Aminoglycoside Genes	NA
**Luz19 + Aztreonam**	aac(6')-29B	0	NA	NA	NA	NA	1	0	9	1	17	Aminoglycoside Genes	NA
**Luz19 + Aztreonam**	aac(6')-33	0.42	0.01	0	8.95	0.1844	3	2	9	9	17	Aminoglycoside Genes	1
**Luz19 + Aztreonam**	aac(6')-Ib-G	0.31	0.17	0.01	3.14	0.2338	2	1	9	6	17	Aminoglycoside Genes	1
**Luz19 + Aztreonam**	aac(6')-IB3	NA	NA	NA	NA	NA	1	1	9	0	17	Aminoglycoside Genes	NA
**Luz19 + Aztreonam**	aac(6')-IIa	NA	NA	NA	NA	NA	1	1	9	0	17	Aminoglycoside Genes	NA
**Luz19 + Aztreonam**	aac(6')-IIc	1.89	14.38	0.07	2786.62	0.3211	2	1	9	1	17	Aminoglycoside Genes	1
**Luz19 + Aztreonam**	aac(6')-Il	7.56	5770	0	6.67+9	0.224	3	4	9	1	17	Aminoglycoside Genes	1
**Luz19 + Aztreonam**	aac(6')-SET	0	NA	NA	NA	NA	1	0	9	1	17	Aminoglycoside Genes	NA
**Luz19 + Aztreonam**	aadA1	0.76	0.67	0.02	18.81	0.8148	4	4	9	10	17	Aminoglycoside Genes	1
**Luz19 + Aztreonam**	aadA2	NA	NA	NA	NA	NA	1	1	9	0	17	Aminoglycoside Genes	NA
**Luz19 + Aztreonam**	aadA6	0	0	0	Inf	0.9975	2	0	9	3	17	Aminoglycoside Genes	1
**Luz19 + Aztreonam**	aadA7	0	NA	NA	NA	NA	1	0	9	1	17	Aminoglycoside Genes	NA
**Luz19 + Aztreonam**	ant(2")-la	5.67	6047	0.01	5.91E+9	0.2159	2	3	9	1	17	Aminoglycoside Genes	1
**Luz19 + Aztreonam**	aph(3')-Ia	NA	NA	NA	NA	NA	1	1	9	0	17	Aminoglycoside Genes	NA
**Luz19 + Aztreonam**	aph(3')-Ib	0	NA	NA	NA	NA	1	0	9	1	17	Aminoglycoside Genes	NA
**Luz19 + Aztreonam**	aph(3')-IIb	1.13	3.422E+7	0	Inf	0.9981	12	9	9	15	17	Aminoglycoside Genes	1
**Luz19 + Aztreonam**	aph(6)-Id	NA	NA	NA	NA	NA	1	1	9	0	17	Aminoglycoside Genes	NA
**Luz19 + Aztreonam**	bcr1	1.06	2.099E+7	0	Inf	0.9982	12	9	9	16	17	Phenicols Bicyclomycins Genes	1
**Luz19 + Aztreonam**	catB7	0.47	0.09	0	6.06	0.2655	4	1	9	4	17	Phenicols Bicyclomycins Genes	1
**Luz19 + Aztreonam**	cmlA5	NA	NA	NA	NA	NA	1	1	9	0	17	Phenicols Bicyclomycins Genes	NA
**Luz19 + Aztreonam**	cmlA6	NA	NA	NA	NA	NA	1	1	9	0	17	Phenicols Bicyclomycins Genes	NA
**Luz19 + Aztreonam**	cmlB	0	NA	NA	NA	NA	1	0	9	1	17	Phenicols Bicyclomycins Genes	NA
**Luz19 + Aztreonam**	crpP	0	0	0	Inf	0.9981	2	0	9	2	17	Quinolone Genes	1

bioRxiv preprint doi: https://doi.org/10.64898/2026.09.27.754834; this version posted September 29, 2026. The copyright holder for this preprint (which was not certified by peer review) is the author/funder, who has granted bioRxiv a license to display the preprint in perpetuity. It is made available under a CC-BY 4.0 International license.

**Luz19 + Aztreonam**	dfrB2	3.78	NA	NA	NA	NA	1	2	9	1	17	Trimethoprim Genes	NA
**Luz19 + Aztreonam**	dfrB5	NA	1.983E+8	0	Inf	0.9978	2	2	9	0	17	Trimethoprim Genes	1
**Luz19 + Aztreonam**	floR2	NA	8474E+12	0	Inf	0.9982	3	4	9	0	17	Phenicols Bicyclomycins Genes	1
**Luz19 + Aztreonam**	fosA	1.06	2099E+6	0	Inf	0.9982	12	9	9	16	17	Fosfomycin Genes	1
**Luz19 + Aztreonam**	GES-1	NA	NA	NA	NA	NA	1	1	9	0	17	Beta lactam Genes	NA
**Luz19 + Aztreonam**	GES-19	0.54	0.29	0.01	8.04	0.4618	2	2	9	7	17	Beta lactam Genes	1
**Luz19 + Aztreonam**	GES-20	0.24	0.06	0	1.31	0.0747	3	1	9	8	17	Beta lactam Genes	0.7861
**Luz19 + Aztreonam**	GES-26	0.94	0.94	0.06	13.88	0.9657	2	1	9	2	17	Beta lactam Genes	1
**Luz19 + Aztreonam**	IMP-1	0	NA	NA	NA	NA	1	0	9	1	17	Beta lactam Genes	NA
**Luz19 + Aztreonam**	KPC-5	NA	NA	NA	NA	NA	1	1	9	0	17	Beta lactam Genes	NA
**Luz19 + Aztreonam**	mexA	1	NA	NA	NA	NA	13	9	9	17	17	Efflux pumps Other Genes	NA
**Luz19 + Aztreonam**	mexE	1	NA	NA	NA	NA	13	9	9	17	17	Efflux pumps Other Genes	NA
**Luz19 + Aztreonam**	mexX	0.79	0.07	0	1.76	0.1072	7	5	9	12	17	Efflux pumps Other Genes	0.8232
**Luz19 + Aztreonam**	NDM-1	NA	NA	NA	NA	NA	1	2	9	0	17	Beta lactam Genes	NA
**Luz19 + Aztreonam**	OXA-10	5.67	6047.08	0.01	5.91E+9	0.2159	2	3	9	1	17	Beta lactam Genes	1
**Luz19 + Aztreonam**	OXA-101	0	NA	NA	NA	NA	1	0	9	1	17	Beta lactam Genes	NA
**Luz19 + Aztreonam**	OXA-2	0.63	0.48	0.02	9.48	0.6315	4	3	9	9	17	Beta lactam Genes	1
**Luz19 + Aztreonam**	OXA-395	1.89	18.49	0.17	2055.48	0.2248	2	1	9	1	17	Beta lactam Genes	1
**Luz19 + Aztreonam**	OXA-396	NA	NA	NA	NA	NA	0	0	9	0	17	Beta lactam Genes	NA
**Luz19 + Aztreonam**	OXA-4	NA	NA	NA	NA	NA	1	1	9	0	17	Beta lactam Genes	NA
**Luz19 + Aztreonam**	OXA-486	1.89	0.97	0.02	50.07	0.9862	2	1	9	1	17	Beta lactam Genes	1
**Luz19 + Aztreonam**	OXA-488	0	0	0	Inf	0.9977	2	0	9	3	17	Beta lactam Genes	1
**Luz19 + Aztreonam**	OXA-494	0	NA	NA	NA	NA	1	0	9	2	17	Beta lactam Genes	NA
**Luz19 + Aztreonam**	OXA-50	NA	NA	NA	NA	NA	1	1	9	0	17	Beta lactam Genes	NA
**Luz19 + Aztreonam**	OXA-846	0.84	1.32	0.03	50.16	0.8826	3	4	9	9	17	Beta lactam Genes	1
**Luz19 + Aztreonam**	OXA-848	0	NA	NA	NA	NA	1	0	9	1	17	Beta lactam Genes	NA
**Luz19 + Aztreonam**	OXA-9	0	NA	NA	NA	NA	1	0	9	1	17	Beta lactam Genes	NA
**Luz19 + Aztreonam**	OXA-914	NA	NA	NA	NA	NA	0	1	9	0	17	Beta lactam Genes	NA
**Luz19 + Aztreonam**	OXA-937	NA	NA	NA	NA	NA	1	1	9	0	17	Beta lactam Genes	NA
**Luz19 + Aztreonam**	PAO	NA	NA	NA	NA	NA	0	0	9	0	17	Beta lactam Genes	NA
**Luz19 + Aztreonam**	PDC-103	NA	NA	NA	NA	NA	1	1	9	0	17	Beta lactam Genes	NA
**Luz19 + Aztreonam**	PDC-11	3.78	NA	NA	NA	NA	1	2	9	1	17	Beta lactam Genes	NA
**Luz19 + Aztreonam**	PDC-15	0	NA	NA	NA	NA	1	0	9	2	17	Beta lactam Genes	NA
**Luz19 + Aztreonam**	PDC-16	0	NA	NA	NA	NA	1	0	9	1	17	Beta lactam Genes	NA
**Luz19 + Aztreonam**	PDC-173	NA	NA	NA	NA	NA	0	0	9	0	17	Beta lactam Genes	NA
**Luz19 + Aztreonam**	PDC-19A	0.47	0.01	0	12.64	0.1857	2	2	9	8	17	Beta lactam Genes	1

bioRxiv preprint doi: https://doi.org/10.64898/2026.09.27.754834; this version posted September 29, 2026. The copyright holder for this preprint (which was not certified by peer review) is the author/funder, who has granted bioRxiv a license to display the preprint in perpetuity. It is made available under a CC-BY 4.0 International license.

**Luz19 + Aztreonam**	PDC-3	1.89	2.85	0.12	65.19	0.5123	4	2	9	2	17	Beta lactam Genes	1
**Luz19 + Aztreonam**	PDC-34	NA	NA	NA	NA	NA	0	0	9	0	17	Beta lactam Genes	NA
**Luz19 + Aztreonam**	PDC-35	0	NA	NA	NA	NA	1	0	9	2	17	Beta lactam Genes	NA
**Luz19 + Aztreonam**	PDC-36	NA	NA	NA	NA	NA	1	1	9	0	17	Beta lactam Genes	NA
**Luz19 + Aztreonam**	PDC-37	0	NA	NA	NA	NA	1	0	9	1	17	Beta lactam Genes	NA
**Luz19 + Aztreonam**	PDC-407	NA	NA	NA	NA	NA	0	0	9	0	17	Beta lactam Genes	NA
**Luz19 + Aztreonam**	PDC-5	NA	NA	NA	NA	NA	0	1	9	0	17	Beta lactam Genes	NA
**Luz19 + Aztreonam**	PDC-8	NA	NA	NA	NA	NA	0	0	9	0	17	Beta lactam Genes	NA
**Luz19 + Aztreonam**	PME-1	NA	NA	NA	NA	NA	1	2	9	0	17	Beta lactam Genes	NA
**Luz19 + Aztreonam**	qacEdelta1	0.42	0.01	0	8.95	0.1844	3	2	9	9	17	Efflux pumps Other Genes	1
**Luz19 + Aztreonam**	qacG2	0	NA	NA	NA	NA	1	0	9	1	17	Efflux pumps Other Genes	NA
**Luz19 + Aztreonam**	rmtD2	NA	NA	NA	NA	NA	1	2	9	0	17	Aminoglycoside Genes	NA
**Luz19 + Aztreonam**	strA	NA	NA	NA	NA	NA	1	1	9	0	17	Aminoglycoside Genes	NA
**Luz19 + Aztreonam**	sul1	0.87	1.35	0.12	15.19	0.807	9	6	9	13	17	Sulfonamides Genes	1
**Luz19 + Aztreonam**	tet(C)	NA	NA	NA	NA	NA	0	0	9	0	17	Tetracyclines Genes	NA
**Luz19 + Aztreonam**	tet(G)	NA	8474E+11	0	Inf	0.9982	3	4	9	0	17	Tetracyclines Genes	1
**Luz19 + Aztreonam**	tet(R)	3.78	NA	NA	NA	NA	1	2	9	1	17	Tetracyclines Genes	NA
**Luz19 + Aztreonam**	-VEB 9.00	3.78	NA	NA	NA	NA	1	2	9	1	17	Beta lactam Genes	NA
**Luz19 + Aztreonam**	VIM-11	NA	NA	NA	NA	NA	1	1	9	0	17	Beta lactam Genes	NA
**Luz19 + Aztreonam**	VIM-2	0.94	0.66	0.02	21.57	0.8178	3	1	9	2	17	Beta lactam Genes	1
**Luz19 + Cefepime**	aac(3)-Id	3.54	NA	NA	NA	NA	1	5	12	2	17	Aminoglycoside Genes	NA
**Luz19 + Cefepime**	aac(6')-29B	0	NA	NA	NA	NA	1	0	12	1	17	Aminoglycoside Genes	NA
**Luz19 + Cefepime**	aac(6')-33	0.31	0.02	0	9.16	0.214	3	2	12	9	17	Aminoglycoside Genes	1
**Luz19 + Cefepime**	aac(6')-Ib-G	0.24	0.18	0.01	3.27	0.2466	2	1	12	6	17	Aminoglycoside Genes	1
**Luz19 + Cefepime**	aac(6')-IB3	NA	NA	NA	NA	NA	1	1	12	0	17	Aminoglycoside Genes	NA
**Luz19 + Cefepime**	aac(6')-IIa	NA	NA	NA	NA	NA	1	1	12	0	17	Aminoglycoside Genes	NA
**Luz19 + Cefepime**	aac(6')-IIc	1.42	11.03	0.09	1310.83	0.3247	2	1	12	1	17	Aminoglycoside Genes	1
**Luz19 + Cefepime**	aac(6')-Il	3.78	9.21	0.7	120.63	0.0907	3	8	12	3	17	Aminoglycoside Genes	0.7944
**Luz19 + Cefepime**	aac(6')-SET	0	NA	NA	NA	NA	1	0	12	1	17	Aminoglycoside Genes	NA
**Luz19 + Cefepime**	aadA1	0.57	0.52	0.02	13.03	0.6912	4	4	12	10	17	Aminoglycoside Genes	1
**Luz19 + Cefepime**	aadA2	3.54	NA	NA	NA	NA	1	5	12	2	17	Aminoglycoside Genes	NA
**Luz19 + Cefepime**	aadA6	0	0	0	Inf	0.9976	2	0	12	3	17	Aminoglycoside Genes	1
**Luz19 + Cefepime**	aadA7	0	NA	NA	NA	NA	1	0	12	1	17	Aminoglycoside Genes	NA
**Luz19 + Cefepime**	ant(2")-la	4.25	39.28	0.73	2112.23	0.071	2	3	12	1	17	Aminoglycoside Genes	0.7861
**Luz19 + Cefepime**	aph(3')-Ia	NA	NA	NA	NA	NA	1	1	12	0	17	Aminoglycoside Genes	NA

bioRxiv preprint doi: https://doi.org/10.64898/2026.09.27.754834; this version posted September 29, 2026. The copyright holder for this preprint (which was not certified by peer review) is the author/funder, who has granted bioRxiv a license to display the preprint in perpetuity. It is made available under a CC-BY 4.0 International license.

**Luz19 + Cefepime**	aph(3')-Ib	0	NA	NA	NA	NA	1	0	12	1	17	Aminoglycoside Genes	NA
**Luz19 + Cefepime**	aph(3')-IIb	1.06	9649E+6	0	Inf	0.998	11	12	12	16	17	Aminoglycoside Genes	1
**Luz19 + Cefepime**	aph(6)-Id	NA	NA	NA	NA	NA	1	1	12	0	17	Aminoglycoside Genes	NA
**Luz19 + Cefepime**	bcr1	1.06	7171E+6	0	Inf	0.9981	10	12	12	16	17	Phenicols Bicyclomycins Genes	1
**Luz19 + Cefepime**	catB7	0	0	0	Inf	0.9976	3	0	12	3	17	Phenicols Bicyclomycins Genes	1
**Luz19 + Cefepime**	cmlA5	NA	NA	NA	NA	NA	1	1	12	0	17	Phenicols Bicyclomycins Genes	NA
**Luz19 + Cefepime**	cmlA6	3.54	NA	NA	NA	NA	1	5	12	2	17	Phenicols Bicyclomycins Genes	NA
**Luz19 + Cefepime**	cmlB	0	NA	NA	NA	NA	1	0	12	1	17	Phenicols Bicyclomycins Genes	NA
**Luz19 + Cefepime**	crpP	0	0	0	Inf	0.9981	2	0	12	2	17	Quinolone Genes	1
**Luz19 + Cefepime**	dfrB2	2.83	NA	NA	NA	NA	1	2	12	1	17	Trimethoprim Genes	NA
**Luz19 + Cefepime**	dfrB5	4.25	12.52	0.23	681.07	0.2151	2	6	12	2	17	Trimethoprim Genes	1
**Luz19 + Cefepime**	floR2	5.67	26.7	0.85	839.68	0.0619	3	8	12	2	17	Phenicols Bicyclomycins Genes	0.7861
**Luz19 + Cefepime**	fosA	1.06	7171E+6	0	Inf	0.9981	10	12	12	16	17	Fosfomycin Genes	1
**Luz19 + Cefepime**	GES-1	NA	NA	NA	NA	NA	1	1	12	0	17	Beta lactam Genes	NA
**Luz19 + Cefepime**	GES-19	0.4	0.32	0.01	8.52	0.4994	2	2	12	7	17	Beta lactam Genes	1
**Luz19 + Cefepime**	GES-20	0.18	0.07	0	1.51	0.0908	3	1	12	8	17	Beta lactam Genes	0.7944
**Luz19 + Cefepime**	GES-26	0.71	1.07	0.08	14.78	0.9593	2	1	12	2	17	Beta lactam Genes	1
**Luz19 + Cefepime**	IMP-1	0	NA	NA	NA	NA	1	0	12	1	17	Beta lactam Genes	NA
**Luz19 + Cefepime**	KPC-5	NA	NA	NA	NA	NA	1	1	12	0	17	Beta lactam Genes	NA
**Luz19 + Cefepime**	mexA	1	NA	NA	NA	NA	11	12	12	17	17	Efflux pumps Other Genes	NA
**Luz19 + Cefepime**	mexE	1	NA	NA	NA	NA	11	12	12	17	17	Efflux pumps Other Genes	NA
**Luz19 + Cefepime**	mexX	1.06	0.21	0.01	4.68	0.3274	6	9	12	12	17	Efflux pumps Other Genes	1
**Luz19 + Cefepime**	NDM-1	NA	NA	NA	NA	NA	1	2	12	0	17	Beta lactam Genes	NA
**Luz19 + Cefepime**	OXA-10	4.25	39.28	0.73	2112.23	0.071	2	3	12	1	17	Beta lactam Genes	0.7861
**Luz19 + Cefepime**	OXA-101	0	NA	NA	NA	NA	1	0	12	1	17	Beta lactam Genes	NA
**Luz19 + Cefepime**	OXA-2	0.47	0.54	0.03	9.81	0.6742	4	3	12	9	17	Beta lactam Genes	1
**Luz19 + Cefepime**	OXA-395	1.42	10.39	0.19	575.03	0.2531	2	1	12	1	17	Beta lactam Genes	1
**Luz19 + Cefepime**	OXA-396	NA	NA	NA	NA	NA	0	0	12	0	17	Beta lactam Genes	NA
**Luz19 + Cefepime**	OXA-4	3.54	NA	NA	NA	NA	1	5	12	2	17	Beta lactam Genes	NA
**Luz19 + Cefepime**	OXA-486	2.36	0.47	0.01	28.34	0.7172	2	5	12	3	17	Beta lactam Genes	1
**Luz19 + Cefepime**	OXA-488	0	NA	NA	NA	NA	1	0	12	2	17	Beta lactam Genes	NA
**Luz19 + Cefepime**	OXA-494	0	NA	NA	NA	NA	1	0	12	1	17	Beta lactam Genes	NA
**Luz19 + Cefepime**	OXA-50	NA	NA	NA	NA	NA	1	1	12	0	17	Beta lactam Genes	NA
**Luz19 + Cefepime**	OXA-846	0.63	1.01	0.03	31.15	0.9947	3	4	12	9	17	Beta lactam Genes	1

bioRxiv preprint doi: https://doi.org/10.64898/2026.09.27.754834; this version posted September 29, 2026. The copyright holder for this preprint (which was not certified by peer review) is the author/funder, who has granted bioRxiv a license to display the preprint in perpetuity. It is made available under a CC-BY 4.0 International license.

**Luz19 + Cefepime**	OXA-848	0	NA	NA	NA	NA	1	0	12	1	17	Beta lactam Genes	NA
**Luz19 + Cefepime**	OXA-9	0	NA	NA	NA	NA	1	0	12	1	17	Beta lactam Genes	NA
**Luz19 + Cefepime**	OXA-914	NA	NA	NA	NA	NA	0	1	12	0	17	Beta lactam Genes	NA
**Luz19 + Cefepime**	OXA-937	NA	NA	NA	NA	NA	0	0	12	0	17	Beta lactam Genes	NA
**Luz19 + Cefepime**	PAO	NA	NA	NA	NA	NA	0	0	12	0	17	Beta lactam Genes	NA
**Luz19 + Cefepime**	PDC-103	NA	NA	NA	NA	NA	1	1	12	0	17	Beta lactam Genes	NA
**Luz19 + Cefepime**	PDC-11	2.83	NA	NA	NA	NA	1	2	12	1	17	Beta lactam Genes	NA
**Luz19 + Cefepime**	PDC-15	0	NA	NA	NA	NA	1	0	12	1	17	Beta lactam Genes	NA
**Luz19 + Cefepime**	PDC-16	0	NA	NA	NA	NA	1	0	12	1	17	Beta lactam Genes	NA
**Luz19 + Cefepime**	PDC-173	NA	NA	NA	NA	NA	0	0	12	0	17	Beta lactam Genes	NA
**Luz19 + Cefepime**	PDC-19A	0.35	0.01	0	12.55	0.2124	2	2	12	8	17	Beta lactam Genes	1
**Luz19 + Cefepime**	PDC-3	1.77	0.41	0.01	17.12	0.6372	3	5	12	4	17	Beta lactam Genes	1
**Luz19 + Cefepime**	PDC-34	NA	NA	NA	NA	NA	0	0	12	0	17	Beta lactam Genes	NA
**Luz19 + Cefepime**	PDC-35	0	NA	NA	NA	NA	1	0	12	2	17	Beta lactam Genes	NA
**Luz19 + Cefepime**	PDC-36	NA	NA	NA	NA	NA	1	1	12	0	17	Beta lactam Genes	NA
**Luz19 + Cefepime**	PDC-37	NA	NA	NA	NA	NA	0	0	12	0	17	Beta lactam Genes	NA
**Luz19 + Cefepime**	PDC-407	NA	NA	NA	NA	NA	0	0	12	0	17	Beta lactam Genes	NA
**Luz19 + Cefepime**	PDC-5	NA	NA	NA	NA	NA	0	1	12	0	17	Beta lactam Genes	NA
**Luz19 + Cefepime**	PDC-8	NA	NA	NA	NA	NA	0	0	12	0	17	Beta lactam Genes	NA
**Luz19 + Cefepime**	PME-1	NA	NA	NA	NA	NA	1	2	12	0	17	Beta lactam Genes	NA
**Luz19 + Cefepime**	qacEdelta1	0.31	0.02	0	9.16	0.214	3	2	12	9	17	Efflux pumps Other Genes	1
**Luz19 + Cefepime**	qacG2	0	NA	NA	NA	NA	1	0	12	1	17	Efflux pumps Other Genes	NA
**Luz19 + Cefepime**	rmtD2	NA	NA	NA	NA	NA	1	2	12	0	17	Aminoglycoside Genes	NA
**Luz19 + Cefepime**	strA	NA	NA	NA	NA	NA	1	1	12	0	17	Aminoglycoside Genes	NA
**Luz19 + Cefepime**	sul1	0.94	0.8	0.06	11.44	0.8702	9	10	12	15	17	Sulfonamides Genes	1
**Luz19 + Cefepime**	tet(C)	NA	NA	NA	NA	NA	0	0	12	0	17	Tetracyclines Genes	NA
**Luz19 + Cefepime**	tet(G)	5.67	26.7	0.85	839.68	0.0619	3	8	12	2	17	Tetracyclines Genes	0.7861
**Luz19 + Cefepime**	tet(R)	2.83	NA	NA	NA	NA	1	2	12	1	17	Tetracyclines Genes	NA
**Luz19 + Cefepime**	-VEB 9.00	2.83	NA	NA	NA	NA	1	2	12	1	17	Beta lactam Genes	NA
**Luz19 + Cefepime**	VIM-11	NA	NA	NA	NA	NA	1	1	12	0	17	Beta lactam Genes	NA
**Luz19 + Cefepime**	VIM-2	1.77	0.38	0.01	13.6	0.5924	3	5	12	4	17	Beta lactam Genes	1
**Luz19 + Ceftazidime**	aac(3)-Id	3.09	NA	NA	NA	NA	1	4	11	2	17	Aminoglycoside Genes	NA
**Luz19 + Ceftazidime**	aac(6')-29B	0	NA	NA	NA	NA	1	0	11	1	17	Aminoglycoside Genes	NA

bioRxiv preprint doi: https://doi.org/10.64898/2026.09.27.754834; this version posted September 29, 2026. The copyright holder for this preprint (which was not certified by peer review) is the author/funder, who has granted bioRxiv a license to display the preprint in perpetuity. It is made available under a CC-BY 4.0 International license.

**Luz19 + Ceftazidime**	aac(6')-33	0.34	0.02	0	9.3	0.2167	3	2	11	9	17	Aminoglycoside Genes	1
**Luz19 + Ceftazidime**	aac(6')-Ib-G	0.26	0.18	0.01	3.27	0.2471	2	1	11	6	17	Aminoglycoside Genes	1
**Luz19 + Ceftazidime**	aac(6')-IB3	NA	NA	NA	NA	NA	1	1	11	0	17	Aminoglycoside Genes	NA
**Luz19 + Ceftazidime**	aac(6')-IIa	NA	NA	NA	NA	NA	1	1	11	0	17	Aminoglycoside Genes	NA
**Luz19 + Ceftazidime**	aac(6')-IIc	1.55	11.46	0.1	1255.03	0.3088	2	1	11	1	17	Aminoglycoside Genes	1
**Luz19 + Ceftazidime**	aac(6')-Il	3.61	8.71	0.66	114.99	0.1001	3	7	11	3	17	Aminoglycoside Genes	0.7944
**Luz19 + Ceftazidime**	aac(6')-SET	0	NA	NA	NA	NA	1	0	11	1	17	Aminoglycoside Genes	NA
**Luz19 + Ceftazidime**	aadA1	0.62	0.51	0.02	12.49	0.6774	4	4	11	10	17	Aminoglycoside Genes	1
**Luz19 + Ceftazidime**	aadA2	3.09	NA	NA	NA	NA	1	4	11	2	17	Aminoglycoside Genes	NA
**Luz19 + Ceftazidime**	aadA6	0	0	0	Inf	0.9976	2	0	11	3	17	Aminoglycoside Genes	1
**Luz19 + Ceftazidime**	aadA7	0	NA	NA	NA	NA	1	0	11	1	17	Aminoglycoside Genes	NA
**Luz19 + Ceftazidime**	ant(2")-la	4.64	35.91	0.7	1833.47	0.0743	2	3	11	1	17	Aminoglycoside Genes	0.7861
**Luz19 + Ceftazidime**	aph(3')-Ia	NA	NA	NA	NA	NA	1	1	11	0	17	Aminoglycoside Genes	NA
**Luz19 + Ceftazidime**	aph(3')-Ib	0	NA	NA	NA	NA	1	0	11	1	17	Aminoglycoside Genes	NA
**Luz19 + Ceftazidime**	aph(3')-IIb	1.06	1016E+7	0	Inf	0.998	11	11	11	16	17	Aminoglycoside Genes	1
**Luz19 + Ceftazidime**	aph(6)-Id	NA	NA	NA	NA	NA	1	1	11	0	17	Aminoglycoside Genes	NA
**Luz19 + Ceftazidime**	bcr1	1.06	8365E+6	0	Inf	0.9981	10	11	11	16	17	Phenicols Bicyclomycins Genes	1
**Luz19 + Ceftazidime**	catB7	0	0	0	Inf	0.9976	3	0	11	3	17	Phenicols Bicyclomycins Genes	1
**Luz19 + Ceftazidime**	cmlA5	NA	NA	NA	NA	NA	1	1	11	0	17	Phenicols Bicyclomycins Genes	NA
**Luz19 + Ceftazidime**	cmlA6	3.09	NA	NA	NA	NA	1	4	11	2	17	Phenicols Bicyclomycins Genes	NA
**Luz19 + Ceftazidime**	cmlB	0	NA	NA	NA	NA	1	0	11	1	17	Phenicols Bicyclomycins Genes	NA
**Luz19 + Ceftazidime**	crpP	0	0	0	Inf	0.9981	2	0	11	2	17	Quinolone Genes	1
**Luz19 + Ceftazidime**	dfrB2	3.09	NA	NA	NA	NA	1	2	11	1	17	Trimethoprim Genes	NA
**Luz19 + Ceftazidime**	dfrB5	3.86	11.42	0.22	606.39	0.2294	2	5	11	2	17	Trimethoprim Genes	1
**Luz19 + Ceftazidime**	floR2	5.41	25.27	0.81	793.06	0.0662	3	7	11	2	17	Phenicols Bicyclomycins Genes	0.7861
**Luz19 + Ceftazidime**	fosA	1.06	8365E+7	0	Inf	0.9981	10	11	11	16	17	Fosfomycin Genes	1
**Luz19 + Ceftazidime**	GES-1	NA	NA	NA	NA	NA	1	1	11	0	17	Beta lactam Genes	NA
**Luz19 + Ceftazidime**	GES-19	0.44	0.33	0.01	8.47	0.5004	2	2	11	7	17	Beta lactam Genes	1
**Luz19 + Ceftazidime**	GES-20	0.19	0.08	0	1.55	0.0941	3	1	11	8	17	Beta lactam Genes	0.7944
**Luz19 + Ceftazidime**	GES-26	0.77	1.14	0.08	15.63	0.923	2	1	11	2	17	Beta lactam Genes	1
**Luz19 + Ceftazidime**	IMP-1	0	NA	NA	NA	NA	1	0	11	1	17	Beta lactam Genes	NA
**Luz19 + Ceftazidime**	KPC-5	NA	NA	NA	NA	NA	1	1	11	0	17	Beta lactam Genes	NA
**Luz19 + Ceftazidime**	mexA	1	NA	NA	NA	NA	11	11	11	17	17	Efflux pumps Other Genes	NA
**Luz19 + Ceftazidime**	mexE	1	NA	NA	NA	NA	11	11	11	17	17	Efflux pumps Other Genes	NA
**Luz19 + Ceftazidime**	mexX	1.03	0.22	0.01	4.92	0.3433	6	8	11	12	17	Efflux pumps Other Genes	1

bioRxiv preprint doi: https://doi.org/10.64898/2026.09.27.754834; this version posted September 29, 2026. The copyright holder for this preprint (which was not certified by peer review) is the author/funder, who has granted bioRxiv a license to display the preprint in perpetuity. It is made available under a CC-BY 4.0 International license.

**Luz19 + Ceftazidime**	NDM-1	NA	NA	NA	NA	NA	1	2	11	0	17	Beta lactam Genes	NA
**Luz19 + Ceftazidime**	OXA-10	4.64	35.91	0.7	1833.47	0.0743	2	3	11	1	17	Beta lactam Genes	0.7861
**Luz19 + Ceftazidime**	OXA-101	0	NA	NA	NA	NA	1	0	11	1	17	Beta lactam Genes	NA
**Luz19 + Ceftazidime**	OXA-2	0.52	0.56	0.03	10.43	0.7008	4	3	11	9	17	Beta lactam Genes	1
**Luz19 + Ceftazidime**	OXA-395	1.55	9.89	0.19	523.71	0.2579	2	1	11	1	17	Beta lactam Genes	1
**Luz19 + Ceftazidime**	OXA-396	NA	NA	NA	NA	NA	0	0	11	0	17	Beta lactam Genes	NA
**Luz19 + Ceftazidime**	OXA-4	3.09	NA	NA	NA	NA	1	4	11	2	17	Beta lactam Genes	NA
**Luz19 + Ceftazidime**	OXA-486	2.06	0.4	0.01	26.64	0.6667	2	4	11	3	17	Beta lactam Genes	1
**Luz19 + Ceftazidime**	OXA-488	0	NA	NA	NA	NA	1	0	11	2	17	Beta lactam Genes	NA
**Luz19 + Ceftazidime**	OXA-494	0	NA	NA	NA	NA	1	0	11	1	17	Beta lactam Genes	NA
**Luz19 + Ceftazidime**	OXA-50	NA	NA	NA	NA	NA	1	1	11	0	17	Beta lactam Genes	NA
**Luz19 + Ceftazidime**	OXA-846	0.69	0.97	0.03	29.03	0.9843	3	4	11	9	17	Beta lactam Genes	1
**Luz19 + Ceftazidime**	OXA-848	0	NA	NA	NA	NA	1	0	11	1	17	Beta lactam Genes	NA
**Luz19 + Ceftazidime**	OXA-9	0	NA	NA	NA	NA	1	0	11	1	17	Beta lactam Genes	NA
**Luz19 + Ceftazidime**	OXA-914	NA	NA	NA	NA	NA	0	1	11	0	17	Beta lactam Genes	NA
**Luz19 + Ceftazidime**	OXA-937	NA	NA	NA	NA	NA	0	0	11	0	17	Beta lactam Genes	NA
**Luz19 + Ceftazidime**	PAO	NA	NA	NA	NA	NA	0	0	11	0	17	Beta lactam Genes	NA
**Luz19 + Ceftazidime**	PDC-103	NA	NA	NA	NA	NA	1	1	11	0	17	Beta lactam Genes	NA
**Luz19 + Ceftazidime**	PDC-11	3.09	NA	NA	NA	NA	1	2	11	1	17	Beta lactam Genes	NA
**Luz19 + Ceftazidime**	PDC-15	0	NA	NA	NA	NA	1	0	11	1	17	Beta lactam Genes	NA
**Luz19 + Ceftazidime**	PDC-16	0	NA	NA	NA	NA	1	0	11	1	17	Beta lactam Genes	NA
**Luz19 + Ceftazidime**	PDC-173	NA	NA	NA	NA	NA	0	0	11	0	17	Beta lactam Genes	NA
**Luz19 + Ceftazidime**	PDC-19A	0.39	0.01	0	12.19	0.2143	2	2	11	8	17	Beta lactam Genes	1
**Luz19 + Ceftazidime**	PDC-3	1.55	0.35	0.01	15.65	0.5866	3	4	11	4	17	Beta lactam Genes	1
**Luz19 + Ceftazidime**	PDC-34	NA	NA	NA	NA	NA	0	0	11	0	17	Beta lactam Genes	NA
**Luz19 + Ceftazidime**	PDC-35	0	NA	NA	NA	NA	1	0	11	2	17	Beta lactam Genes	NA
**Luz19 + Ceftazidime**	PDC-36	NA	NA	NA	NA	NA	1	1	11	0	17	Beta lactam Genes	NA
**Luz19 + Ceftazidime**	PDC-37	NA	NA	NA	NA	NA	0	0	11	0	17	Beta lactam Genes	NA
**Luz19 + Ceftazidime**	PDC-407	NA	NA	NA	NA	NA	0	0	11	0	17	Beta lactam Genes	NA
**Luz19 + Ceftazidime**	PDC-5	NA	NA	NA	NA	NA	0	1	11	0	17	Beta lactam Genes	NA
**Luz19 + Ceftazidime**	PDC-8	NA	NA	NA	NA	NA	0	0	11	0	17	Beta lactam Genes	NA
**Luz19 + Ceftazidime**	PME-1	NA	NA	NA	NA	NA	1	2	11	0	17	Beta lactam Genes	NA
**Luz19 + Ceftazidime**	qacEdelta1	0.34	0.02	0	9.3	0.2167	3	2	11	9	17	Efflux pumps Other Genes	1
**Luz19 + Ceftazidime**	qacG2	0	NA	NA	NA	NA	1	0	11	1	17	Efflux pumps Other Genes	NA

bioRxiv preprint doi: https://doi.org/10.64898/2026.09.27.754834; this version posted September 29, 2026. The copyright holder for this preprint (which was not certified by peer review) is the author/funder, who has granted bioRxiv a license to display the preprint in perpetuity. It is made available under a CC-BY 4.0 International license.

**Luz19 + Ceftazidime**	rmtD2	NA	NA	NA	NA	NA	1	2	11	0	17	Aminoglycoside Genes	NA
**Luz19 + Ceftazidime**	strA	NA	NA	NA	NA	NA	1	1	11	0	17	Aminoglycoside Genes	NA
**Luz19 + Ceftazidime**	sul1	0.93	0.76	0.05	10.77	0.8392	9	9	11	15	17	Sulfonamides Genes	1
**Luz19 + Ceftazidime**	tet(C)	NA	NA	NA	NA	NA	0	0	11	0	17	Tetracyclines Genes	NA
**Luz19 + Ceftazidime**	tet(G)	5.41	25.27	0.81	793.06	0.0662	3	7	11	2	17	Tetracyclines Genes	0.7861
**Luz19 + Ceftazidime**	tet(R)	3.09	NA	NA	NA	NA	1	2	11	1	17	Tetracyclines Genes	NA
**Luz19 + Ceftazidime**	-VEB 9.00	3.09	NA	NA	NA	NA	1	2	11	1	17	Beta lactam Genes	NA
**Luz19 + Ceftazidime**	VIM-11	NA	NA	NA	NA	NA	1	1	11	0	17	Beta lactam Genes	NA
**Luz19 + Ceftazidime**	VIM-2	1.55	0.33	0.01	12.99	0.5508	3	4	11	4	17	Beta lactam Genes	1
**Luz19 + Ciprofloxacin**	aac(3)-Id	4.26	NA	NA	NA	NA	1	5	17	2	29	Aminoglycoside Genes	NA
**Luz19 + Ciprofloxacin**	aac(6')-29B	0	NA	NA	NA	NA	1	0	17	1	29	Aminoglycoside Genes	NA
**Luz19 + Ciprofloxacin**	aac(6')-33	0.38	0.01	0	2.3	0.0986	3	2	17	9	29	Aminoglycoside Genes	0.7944
**Luz19 + Ciprofloxacin**	aac(6')-Ib-G	0.85	1.17	0.15	9.1	0.8795	3	3	17	6	29	Aminoglycoside Genes	1
**Luz19 + Ciprofloxacin**	aac(6')-IB3	NA	NA	NA	NA	NA	1	1	17	0	29	Aminoglycoside Genes	NA
**Luz19 + Ciprofloxacin**	aac(6')-IIa	NA	NA	NA	NA	NA	1	1	17	0	29	Aminoglycoside Genes	NA
**Luz19 + Ciprofloxacin**	aac(6')-IIc	1.71	4.35	0.13	142.77	0.4091	2	1	17	1	29	Aminoglycoside Genes	1
**Luz19 + Ciprofloxacin**	aac(6')-Il	4.55	3.37	0.49	23.33	0.2186	3	8	17	3	29	Aminoglycoside Genes	1
**Luz19 + Ciprofloxacin**	aac(6')-SET	0	NA	NA	NA	NA	1	0	17	1	29	Aminoglycoside Genes	NA
**Luz19 + Ciprofloxacin**	aadA1	0.68	0.18	0.01	3.17	0.2442	4	4	17	10	29	Aminoglycoside Genes	1
**Luz19 + Ciprofloxacin**	aadA2	4.26	NA	NA	NA	NA	1	5	17	2	29	Aminoglycoside Genes	NA
**Luz19 + Ciprofloxacin**	aadA6	1.14	1.51	0.21	10.98	0.6849	2	2	17	3	29	Aminoglycoside Genes	1
**Luz19 + Ciprofloxacin**	aadA7	0	NA	NA	NA	NA	1	0	17	1	29	Aminoglycoside Genes	NA
**Luz19 + Ciprofloxacin**	ant(2")-la	1.71	2.95	0.36	24.36	0.3149	4	3	17	3	29	Aminoglycoside Genes	1
**Luz19 + Ciprofloxacin**	aph(3')-Ia	NA	NA	NA	NA	NA	1	1	17	0	29	Aminoglycoside Genes	NA
**Luz19 + Ciprofloxacin**	aph(3')-Ib	0	NA	NA	NA	NA	1	0	17	1	29	Aminoglycoside Genes	NA
**Luz19 + Ciprofloxacin**	aph(3')-IIb	0.95	0.29	0.03	2.68	0.2775	17	15	17	27	29	Aminoglycoside Genes	1
**Luz19 + Ciprofloxacin**	aph(6)-Id	NA	3827E+8	0	Inf	0.9974	2	3	17	0	29	Aminoglycoside Genes	1
**Luz19 + Ciprofloxacin**	bcr1	1.04	1553E+7	0	Inf	0.9988	18	17	17	28	29	Phenicols Bicyclomycins Genes	1
**Luz19 + Ciprofloxacin**	catB7	0.14	0.1	0.01	1.41	0.0885	8	1	17	12	29	Phenicols Bicyclomycins Genes	0.7944
**Luz19 + Ciprofloxacin**	cmlA5	NA	NA	NA	NA	NA	1	1	17	0	29	Phenicols Bicyclomycins Genes	NA
**Luz19 + Ciprofloxacin**	cmlA6	4.26	NA	NA	NA	NA	1	5	17	2	29	Phenicols Bicyclomycins Genes	NA
**Luz19 + Ciprofloxacin**	cmlB	0	NA	NA	NA	NA	1	0	17	1	29	Phenicols Bicyclomycins Genes	NA
**Luz19 + Ciprofloxacin**	crpP	0	0	0	Inf	0.998	2	0	17	2	29	Quinolone Genes	1
**Luz19 + Ciprofloxacin**	dfrB2	3.41	NA	NA	NA	NA	1	2	17	1	29	Trimethoprim Genes	NA

bioRxiv preprint doi: https://doi.org/10.64898/2026.09.27.754834; this version posted September 29, 2026. The copyright holder for this preprint (which was not certified by peer review) is the author/funder, who has granted bioRxiv a license to display the preprint in perpetuity. It is made available under a CC-BY 4.0 International license.

**Luz19 + Ciprofloxacin**	dfrB5	5.12	2.86	0.21	38.75	0.4303	2	6	17	2	29	Trimethoprim Genes	1
**Luz19 + Ciprofloxacin**	floR2	6.82	5.91	0.61	57.44	0.1255	3	8	17	2	29	Phenicols Bicyclomycins Genes	0.8931
**Luz19 + Ciprofloxacin**	fosA	1.12	87712262.44	0	Inf	0.9985	16	17	17	26	29	Fosfomycin Genes	1
**Luz19 + Ciprofloxacin**	GES-1	NA	NA	NA	NA	NA	1	1	17	0	29	Beta lactam Genes	NA
**Luz19 + Ciprofloxacin**	GES-19	0.49	0.15	0.01	2.93	0.2123	2	2	17	7	29	Beta lactam Genes	1
**Luz19 + Ciprofloxacin**	GES-20	0.21	0.05	0	0.97	0.048	3	1	17	8	29	Beta lactam Genes	0.7744
**Luz19 + Ciprofloxacin**	GES-26	0.85	0.83	0.06	10.71	0.8832	2	1	17	2	29	Beta lactam Genes	1
**Luz19 + Ciprofloxacin**	IMP-1	0	NA	NA	NA	NA	1	0	17	1	29	Beta lactam Genes	NA
**Luz19 + Ciprofloxacin**	KPC-5	NA	NA	NA	NA	NA	1	1	17	0	29	Beta lactam Genes	NA
**Luz19 + Ciprofloxacin**	mexA	1	NA	NA	NA	NA	19	17	17	29	29	Efflux pumps Other Genes	NA
**Luz19 + Ciprofloxacin**	mexE	1	NA	NA	NA	NA	19	17	17	29	29	Efflux pumps Other Genes	NA
**Luz19 + Ciprofloxacin**	mexX	0.81	0.18	0.03	1.13	0.0666	12	10	17	21	29	Efflux pumps Other Genes	0.7861
**Luz19 + Ciprofloxacin**	NDM-1	NA	NA	NA	NA	NA	1	2	17	0	29	Beta lactam Genes	NA
**Luz19 + Ciprofloxacin**	OXA-10	5.12	8.98	0.64	126.02	0.1033	2	3	17	1	29	Beta lactam Genes	0.8068
**Luz19 + Ciprofloxacin**	OXA-101	0	NA	NA	NA	NA	1	0	17	1	29	Beta lactam Genes	NA
**Luz19 + Ciprofloxacin**	OXA-2	0.57	0.22	0.01	3.27	0.2692	4	3	17	9	29	Beta lactam Genes	1
**Luz19 + Ciprofloxacin**	OXA-395	0.34	2.33	0.11	49.28	0.587	3	1	17	5	29	Beta lactam Genes	1
**Luz19 + Ciprofloxacin**	OXA-396	0	NA	NA	NA	NA	1	0	17	1	29	Beta lactam Genes	NA
**Luz19 + Ciprofloxacin**	OXA-4	4.26	NA	NA	NA	NA	1	5	17	2	29	Beta lactam Genes	NA
**Luz19 + Ciprofloxacin**	OXA-486	2.84	0.48	0.03	8.68	0.6226	2	5	17	3	29	Beta lactam Genes	1
**Luz19 + Ciprofloxacin**	OXA-488	2.27	5.21	0.82	33	0.0794	3	4	17	3	29	Beta lactam Genes	0.7944
**Luz19 + Ciprofloxacin**	OXA-494	0	NA	NA	NA	NA	1	0	17	2	29	Beta lactam Genes	NA
**Luz19 + Ciprofloxacin**	OXA-50	0.43	0.44	0.04	5.05	0.5104	5	1	17	4	29	Beta lactam Genes	1
**Luz19 + Ciprofloxacin**	OXA-846	0.76	0.27	0.01	4.94	0.3783	3	4	17	9	29	Beta lactam Genes	1
**Luz19 + Ciprofloxacin**	OXA-848	0	NA	NA	NA	NA	1	0	17	2	29	Beta lactam Genes	NA
**Luz19 + Ciprofloxacin**	OXA-9	0	NA	NA	NA	NA	1	0	17	1	29	Beta lactam Genes	NA
**Luz19 + Ciprofloxacin**	OXA-914	NA	NA	NA	NA	NA	0	1	17	0	29	Beta lactam Genes	NA
**Luz19 + Ciprofloxacin**	OXA-937	1.71	NA	NA	NA	NA	1	1	17	1	29	Beta lactam Genes	NA
**Luz19 + Ciprofloxacin**	PAO	0	0	0	Inf	0.9979	2	0	17	2	29	Beta lactam Genes	1
**Luz19 + Ciprofloxacin**	PDC-103	NA	NA	NA	NA	NA	1	1	17	0	29	Beta lactam Genes	NA
**Luz19 + Ciprofloxacin**	PDC-11	3.41	NA	NA	NA	NA	1	2	17	1	29	Beta lactam Genes	NA
**Luz19 + Ciprofloxacin**	PDC-15	0	NA	NA	NA	NA	1	0	17	1	29	Beta lactam Genes	NA
**Luz19 + Ciprofloxacin**	PDC-16	0	NA	NA	NA	NA	1	0	17	2	29	Beta lactam Genes	NA
**Luz19 + Ciprofloxacin**	PDC-173	0	NA	NA	NA	NA	1	0	17	1	29	Beta lactam Genes	NA

bioRxiv preprint doi: https://doi.org/10.64898/2026.09.27.754834; this version posted September 29, 2026. The copyright holder for this preprint (which was not certified by peer review) is the author/funder, who has granted bioRxiv a license to display the preprint in perpetuity. It is made available under a CC-BY 4.0 International license.

**Luz19 + Ciprofloxacin**	PDC-19A	0.43	0.01	0	2.25	0.0927	2	2	17	8	29	Beta lactam Genes	0.7944
**Luz19 + Ciprofloxacin**	PDC-3	2.05	0.43	0.03	6.96	0.5542	4	6	17	5	29	Beta lactam Genes	1
**Luz19 + Ciprofloxacin**	PDC-34	NA	NA	NA	NA	NA	1	1	17	0	29	Beta lactam Genes	NA
**Luz19 + Ciprofloxacin**	PDC-35	1.71	NA	NA	NA	NA	1	2	17	2	29	Beta lactam Genes	NA
**Luz19 + Ciprofloxacin**	PDC-36	NA	NA	NA	NA	NA	1	1	17	0	29	Beta lactam Genes	NA
**Luz19 + Ciprofloxacin**	PDC-37	1.71	NA	NA	NA	NA	1	1	17	1	29	Beta lactam Genes	NA
**Luz19 + Ciprofloxacin**	PDC-407	0	NA	NA	NA	NA	0	0	17	1	29	Beta lactam Genes	NA
**Luz19 + Ciprofloxacin**	PDC-5	0.43	NA	NA	NA	NA	1	1	17	4	29	Beta lactam Genes	NA
**Luz19 + Ciprofloxacin**	PDC-8	0	0	0	Inf	0.9981	2	0	17	2	29	Beta lactam Genes	1
**Luz19 + Ciprofloxacin**	PME-1	NA	NA	NA	NA	NA	1	2	17	0	29	Beta lactam Genes	NA
**Luz19 + Ciprofloxacin**	qacEdelta1	0.38	0.01	0	2.3	0.0986	3	2	17	9	29	Efflux pumps Other Genes	0.7944
**Luz19 + Ciprofloxacin**	qacG2	0	NA	NA	NA	NA	1	0	17	1	29	Efflux pumps Other Genes	NA
**Luz19 + Ciprofloxacin**	rmtD2	NA	NA	NA	NA	NA	1	2	17	0	29	Aminoglycoside Genes	NA
**Luz19 + Ciprofloxacin**	strA	NA	382662161.51	0	Inf	0.9974	2	3	17	0	29	Aminoglycoside Genes	1
**Luz19 + Ciprofloxacin**	sul1	1.28	1.1	0.22	5.56	0.9056	10	12	17	16	29	Sulfonamides Genes	1
**Luz19 + Ciprofloxacin**	tet(C)	NA	NA	NA	NA	NA	1	2	17	0	29	Tetracyclines Genes	NA
**Luz19 + Ciprofloxacin**	tet(G)	6.82	5.91	0.61	57.44	0.1255	3	8	17	2	29	Tetracyclines Genes	0.8931
**Luz19 + Ciprofloxacin**	tet(R)	3.41	NA	NA	NA	NA	1	2	17	1	29	Tetracyclines Genes	NA
**Luz19 + Ciprofloxacin**	-VEB 9.00	3.41	NA	NA	NA	NA	1	2	17	1	29	Beta lactam Genes	NA
**Luz19 + Ciprofloxacin**	VIM-11	NA	NA	NA	NA	NA	1	1	17	0	29	Beta lactam Genes	NA
**Luz19 + Ciprofloxacin**	VIM-2	2.13	0.41	0.03	5.66	0.5055	3	5	17	4	29	Beta lactam Genes	1
**Luz19 + Doripenem**	aac(3)-Id	7.27	NA	NA	NA	NA	1	4	11	1	20	Aminoglycoside Genes	NA
**Luz19 + Doripenem**	aac(6')-29B	0	NA	NA	NA	NA	1	0	11	1	20	Aminoglycoside Genes	NA
**Luz19 + Doripenem**	aac(6')-33	0.4	0.01	0	7.41	0.184	3	2	11	9	20	Aminoglycoside Genes	1
**Luz19 + Doripenem**	aac(6')-Ib-G	0.3	0.17	0.01	3.18	0.238	2	1	11	6	20	Aminoglycoside Genes	1
**Luz19 + Doripenem**	aac(6')-IB3	NA	NA	NA	NA	NA	1	1	11	0	20	Aminoglycoside Genes	NA
**Luz19 + Doripenem**	aac(6')-IIa	NA	NA	NA	NA	NA	1	1	11	0	20	Aminoglycoside Genes	NA
**Luz19 + Doripenem**	aac(6')-IIc	1.82	21.61	0.12	4019.05	0.2491	2	1	11	1	20	Aminoglycoside Genes	1
**Luz19 + Doripenem**	aac(6')-Il	6.36	14.96	1.14	196.42	0.0394	3	7	11	2	20	Aminoglycoside Genes	0.7744
**Luz19 + Doripenem**	aac(6')-SET	0	NA	NA	NA	NA	1	0	11	1	20	Aminoglycoside Genes	NA
**Luz19 + Doripenem**	aadA1	0.73	0.78	0.03	23.03	0.883	4	4	11	10	20	Aminoglycoside Genes	1
**Luz19 + Doripenem**	aadA2	7.27	NA	NA	NA	NA	1	4	11	1	20	Aminoglycoside Genes	NA
**Luz19 + Doripenem**	aadA6	0	0	0	Inf	0.9977	2	0	11	3	20	Aminoglycoside Genes	1
**Luz19 + Doripenem**	aadA7	0	NA	NA	NA	NA	1	0	11	1	20	Aminoglycoside Genes	NA
**Luz19 + Doripenem**	ant(2")-la	2.73	54.44	0.77	3824.94	0.0654	3	3	11	2	20	Aminoglycoside Genes	0.7861

bioRxiv preprint doi: https://doi.org/10.64898/2026.09.27.754834; this version posted September 29, 2026. The copyright holder for this preprint (which was not certified by peer review) is the author/funder, who has granted bioRxiv a license to display the preprint in perpetuity. It is made available under a CC-BY 4.0 International license.

**Luz19 + Doripenem**	aph(3')-Ia	NA	NA	NA	NA	NA	1	1	11	0	20	Aminoglycoside Genes	NA
**Luz19 + Doripenem**	aph(3')-Ib	0	NA	NA	NA	NA	1	0	11	1	20	Aminoglycoside Genes	NA
**Luz19 + Doripenem**	aph(3')-IIb	1.11	26894940.59	0	Inf	0.9981	13	11	11	18	20	Aminoglycoside Genes	1
**Luz19 + Doripenem**	aph(6)-Id	NA	NA	NA	NA	NA	1	1	11	0	20	Aminoglycoside Genes	NA
**Luz19 + Doripenem**	bcr1	1.05	1552365.32	0	Inf	0.9983	13	11	11	19	20	Phenicols Bicyclomycins Genes	1
**Luz19 + Doripenem**	catB7	0	0	0	Inf	0.9978	5	0	11	6	20	Phenicols Bicyclomycins Genes	1
**Luz19 + Doripenem**	cmlA5	NA	NA	NA	NA	NA	1	1	11	0	20	Phenicols Bicyclomycins Genes	NA
**Luz19 + Doripenem**	cmlA6	7.27	NA	NA	NA	NA	1	4	11	1	20	Phenicols Bicyclomycins Genes	NA
**Luz19 + Doripenem**	cmlB	0	NA	NA	NA	NA	1	0	11	1	20	Phenicols Bicyclomycins Genes	NA
**Luz19 + Doripenem**	crpP	0	0	0	Inf	0.9981	2	0	11	2	20	Quinolone Genes	1
**Luz19 + Doripenem**	dfrB2	3.64	NA	NA	NA	NA	1	2	11	1	20	Trimethoprim Genes	NA
**Luz19 + Doripenem**	dfrB5	9.09	20.83	0.36	1191.39	0.1414	2	5	11	1	20	Trimethoprim Genes	0.9381
**Luz19 + Doripenem**	floR2	12.7	43.04	1.3	1420.11	0.0349	3	7	11	1	20	Phenicols Bicyclomycins Genes	0.7744
**Luz19 + Doripenem**	fosA	1.05	1552365.32	0	Inf	0.9983	13	11	11	19	20	Fosfomycin Genes	1
**Luz19 + Doripenem**	GES-1	NA	NA	NA	NA	NA	1	1	11	0	20	Beta lactam Genes	NA
**Luz19 + Doripenem**	GES-19	0.52	0.3	0.01	8.16	0.4722	2	2	11	7	20	Beta lactam Genes	1
**Luz19 + Doripenem**	GES-20	0.23	0.07	0	1.53	0.091	3	1	11	8	20	Beta lactam Genes	0.7944
**Luz19 + Doripenem**	GES-26	0.91	1.17	0.09	16.02	0.9076	2	1	11	2	20	Beta lactam Genes	1
**Luz19 + Doripenem**	IMP-1	0	NA	NA	NA	NA	1	0	11	1	20	Beta lactam Genes	NA
**Luz19 + Doripenem**	KPC-5	NA	NA	NA	NA	NA	1	1	11	0	20	Beta lactam Genes	NA
**Luz19 + Doripenem**	mexA	1	NA	NA	NA	NA	14	11	11	20	20	Efflux pumps Other Genes	NA
**Luz19 + Doripenem**	mexE	1	NA	NA	NA	NA	14	11	11	20	20	Efflux pumps Other Genes	NA
**Luz19 + Doripenem**	mexX	0.97	0.12	0.01	2.43	0.1657	9	8	11	15	20	Efflux pumps Other Genes	1
**Luz19 + Doripenem**	NDM-1	NA	NA	NA	NA	NA	1	2	11	0	20	Beta lactam Genes	NA
**Luz19 + Doripenem**	OXA-10	5.45	84.99	1.11	6534.52	0.0449	2	3	11	1	20	Beta lactam Genes	0.7744
**Luz19 + Doripenem**	OXA-101	0	NA	NA	NA	NA	1	0	11	1	20	Beta lactam Genes	NA
**Luz19 + Doripenem**	OXA-2	0.61	0.63	0.03	12.93	0.7623	4	3	11	9	20	Beta lactam Genes	1
**Luz19 + Doripenem**	OXA-395	0.91	16.19	0.21	1260.78	0.2101	3	1	11	2	20	Beta lactam Genes	1
**Luz19 + Doripenem**	OXA-396	NA	NA	NA	NA	NA	0	0	11	0	20	Beta lactam Genes	NA
**Luz19 + Doripenem**	OXA-4	7.27	NA	NA	NA	NA	1	4	11	1	20	Beta lactam Genes	NA
**Luz19 + Doripenem**	OXA-486	3.64	1.38	0.04	48.35	0.8608	2	4	11	2	20	Beta lactam Genes	1
**Luz19 + Doripenem**	OXA-488	0	0	0	Inf	0.9978	2	0	11	3	20	Beta lactam Genes	1
**Luz19 + Doripenem**	OXA-494	0	NA	NA	NA	NA	1	0	11	2	20	Beta lactam Genes	NA
**Luz19 + Doripenem**	OXA-50	1.82	6.51	0.17	242.46	0.3099	2	1	11	1	20	Beta lactam Genes	1

bioRxiv preprint doi: https://doi.org/10.64898/2026.09.27.754834; this version posted September 29, 2026. The copyright holder for this preprint (which was not certified by peer review) is the author/funder, who has granted bioRxiv a license to display the preprint in perpetuity. It is made available under a CC-BY 4.0 International license.

**Luz19 + Doripenem**	OXA-846	0.81	1.44	0.04	56.57	0.8468	3	4	11	9	20	Beta lactam Genes	1
**Luz19 + Doripenem**	OXA-848	0	NA	NA	NA	NA	1	0	11	1	20	Beta lactam Genes	NA
**Luz19 + Doripenem**	OXA-9	0	NA	NA	NA	NA	1	0	11	1	20	Beta lactam Genes	NA
**Luz19 + Doripenem**	OXA-914	NA	NA	NA	NA	NA	0	1	11	0	20	Beta lactam Genes	NA
**Luz19 + Doripenem**	OXA-937	NA	NA	NA	NA	NA	0	0	11	0	20	Beta lactam Genes	NA
**Luz19 + Doripenem**	PAO	0	NA	NA	NA	NA	1	0	11	1	20	Beta lactam Genes	NA
**Luz19 + Doripenem**	PDC-103	NA	NA	NA	NA	NA	1	1	11	0	20	Beta lactam Genes	NA
**Luz19 + Doripenem**	PDC-11	3.64	NA	NA	NA	NA	1	2	11	1	20	Beta lactam Genes	NA
**Luz19 + Doripenem**	PDC-15	0	NA	NA	NA	NA	1	0	11	2	20	Beta lactam Genes	NA
**Luz19 + Doripenem**	PDC-16	0	NA	NA	NA	NA	1	0	11	1	20	Beta lactam Genes	NA
**Luz19 + Doripenem**	PDC-173	NA	NA	NA	NA	NA	0	0	11	0	20	Beta lactam Genes	NA
**Luz19 + Doripenem**	PDC-19A	0.45	0.01	0	8.03	0.1752	2	2	11	8	20	Beta lactam Genes	1
**Luz19 + Doripenem**	PDC-3	2.42	1.21	0.04	34.74	0.9109	3	4	11	3	20	Beta lactam Genes	1
**Luz19 + Doripenem**	PDC-34	NA	NA	NA	NA	NA	0	0	11	0	20	Beta lactam Genes	NA
**Luz19 + Doripenem**	PDC-35	0	NA	NA	NA	NA	1	0	11	2	20	Beta lactam Genes	NA
**Luz19 + Doripenem**	PDC-36	NA	NA	NA	NA	NA	1	1	11	0	20	Beta lactam Genes	NA
**Luz19 + Doripenem**	PDC-37	0	NA	NA	NA	NA	1	0	11	1	20	Beta lactam Genes	NA
**Luz19 + Doripenem**	PDC-407	NA	NA	NA	NA	NA	0	0	11	0	20	Beta lactam Genes	NA
**Luz19 + Doripenem**	PDC-5	1.82	NA	NA	NA	NA	1	1	11	1	20	Beta lactam Genes	NA
**Luz19 + Doripenem**	PDC-8	NA	NA	NA	NA	NA	0	0	11	0	20	Beta lactam Genes	NA
**Luz19 + Doripenem**	PME-1	NA	NA	NA	NA	NA	1	2	11	0	20	Beta lactam Genes	NA
**Luz19 + Doripenem**	qacEdelta1	0.4	0.01	0	7.41	0.184	3	2	11	9	20	Efflux pumps Other Genes	1
**Luz19 + Doripenem**	qacG2	0	NA	NA	NA	NA	1	0	11	1	20	Efflux pumps Other Genes	NA
**Luz19 + Doripenem**	rmtD2	NA	NA	NA	NA	NA	1	2	11	0	20	Aminoglycoside Genes	NA
**Luz19 + Doripenem**	strA	NA	NA	NA	NA	NA	1	1	11	0	20	Aminoglycoside Genes	NA
**Luz19 + Doripenem**	sul1	1.09	1.8	0.14	22.57	0.6486	10	9	11	15	20	Sulfonamides Genes	1
**Luz19 + Doripenem**	tet(C)	NA	NA	NA	NA	NA	0	0	11	0	20	Tetracyclines Genes	NA
**Luz19 + Doripenem**	tet(G)	12.7	43.04	1.3	1420.11	0.0349	3	7	11	1	20	Tetracyclines Genes	0.7744
**Luz19 + Doripenem**	tet(R)	3.64	NA	NA	NA	NA	1	2	11	1	20	Tetracyclines Genes	NA
**Luz19 + Doripenem**	-VEB 9.00	3.64	NA	NA	NA	NA	1	2	11	1	20	Beta lactam Genes	NA
**Luz19 + Doripenem**	VIM-11	NA	NA	NA	NA	NA	1	1	11	0	20	Beta lactam Genes	NA
**Luz19 + Doripenem**	VIM-2	2.42	0.91	0.04	19.57	0.9541	3	4	11	3	20	Beta lactam Genes	1
**Luz19 + Tobramycin**	aac(3)-Id	4.11	NA	NA	NA	NA	1	5	14	2	23	Aminoglycoside Genes	NA
**Luz19 + Tobramycin**	aac(6')-29B	0	NA	NA	NA	NA	1	0	14	1	23	Aminoglycoside Genes	NA

bioRxiv preprint doi: https://doi.org/10.64898/2026.09.27.754834; this version posted September 29, 2026. The copyright holder for this preprint (which was not certified by peer review) is the author/funder, who has granted bioRxiv a license to display the preprint in perpetuity. It is made available under a CC-BY 4.0 International license.

**Luz19 + Tobramycin**	aac(6')-33	0.37	0.01	0	4.94	0.1492	3	2	14	9	23	Aminoglycoside Genes	0.9381
**Luz19 + Tobramycin**	aac(6')-Ib-G	0.82	1.26	0.16	10.06	0.8262	3	3	14	6	23	Aminoglycoside Genes	1
**Luz19 + Tobramycin**	aac(6')-IB3	NA	NA	NA	NA	NA	1	1	14	0	23	Aminoglycoside Genes	NA
**Luz19 + Tobramycin**	aac(6')-IIa	NA	NA	NA	NA	NA	1	1	14	0	23	Aminoglycoside Genes	NA
**Luz19 + Tobramycin**	aac(6')-IIc	1.64	4.26	0.11	164.88	0.4369	2	1	14	1	23	Aminoglycoside Genes	1
**Luz19 + Tobramycin**	aac(6')-Il	4.38	3.42	0.39	29.64	0.2646	3	8	14	3	23	Aminoglycoside Genes	1
**Luz19 + Tobramycin**	aac(6')-SET	0	NA	NA	NA	NA	1	0	14	1	23	Aminoglycoside Genes	NA
**Luz19 + Tobramycin**	aadA1	0.66	0.22	0.01	3.61	0.2862	4	4	14	10	23	Aminoglycoside Genes	1
**Luz19 + Tobramycin**	aadA2	4.11	NA	NA	NA	NA	1	5	14	2	23	Aminoglycoside Genes	NA
**Luz19 + Tobramycin**	aadA6	1.1	1.44	0.18	11.33	0.7262	2	2	14	3	23	Aminoglycoside Genes	1
**Luz19 + Tobramycin**	aadA7	0	NA	NA	NA	NA	1	0	14	1	23	Aminoglycoside Genes	NA
**Luz19 + Tobramycin**	ant(2'')-la	1.64	3.17	0.35	29.04	0.3071	4	3	14	3	23	Aminoglycoside Genes	1
**Luz19 + Tobramycin**	aph(3')-Ia	NA	NA	NA	NA	NA	1	1	14	0	23	Aminoglycoside Genes	NA
**Luz19 + Tobramycin**	aph(3')-Ib	0	NA	NA	NA	NA	1	0	14	1	23	Aminoglycoside Genes	NA
**Luz19 + Tobramycin**	aph(3')-IIb	1.02	0.81	0.06	10.79	0.872	13	13	14	21	23	Aminoglycoside Genes	1
**Luz19 + Tobramycin**	aph(6)-Id	NA	409643179.16	0	Inf	0.9974	2	3	14	0	23	Aminoglycoside Genes	1
**Luz19 + Tobramycin**	bcr1	1.05	17699191.9	0	Inf	0.9988	13	14	14	22	23	Phenicols Bicyclomycins Genes	1
**Luz19 + Tobramycin**	catB7	0	0	0	Inf	0.9976	5	0	14	7	23	Phenicols Bicyclomycins Genes	1
**Luz19 + Tobramycin**	cmlA5	NA	NA	NA	NA	NA	1	1	14	0	23	Phenicols Bicyclomycins Genes	NA
**Luz19 + Tobramycin**	cmlA6	4.11	NA	NA	NA	NA	1	5	14	2	23	Phenicols Bicyclomycins Genes	NA
**Luz19 + Tobramycin**	cmlB	0	NA	NA	NA	NA	1	0	14	1	23	Phenicols Bicyclomycins Genes	NA
**Luz19 + Tobramycin**	crpP	0	0	0	Inf	0.998	2	0	14	2	23	Quinolone Genes	1
**Luz19 + Tobramycin**	dfrB2	3.29	NA	NA	NA	NA	1	2	14	1	23	Trimethoprim Genes	NA
**Luz19 + Tobramycin**	dfrB5	4.93	2.47	0.11	53.1	0.5636	2	6	14	2	23	Trimethoprim Genes	1
**Luz19 + Tobramycin**	floR2	6.57	6.8	0.5	91.89	0.149	3	8	14	2	23	Phenicols Bicyclomycins Genes	0.9381
**Luz19 + Tobramycin**	fosA	1.1	19391089.25	0	Inf	0.9982	12	14	14	21	23	Fosfomycin Genes	1
**Luz19 + Tobramycin**	GES-1	NA	NA	NA	NA	NA	1	1	14	0	23	Beta lactam Genes	NA
**Luz19 + Tobramycin**	GES-19	0.47	0.18	0.01	3.48	0.2534	2	2	14	7	23	Beta lactam Genes	1
**Luz19 + Tobramycin**	GES-20	0.21	0.05	0	1.04	0.0528	3	1	14	8	23	Beta lactam Genes	0.7861
**Luz19 + Tobramycin**	GES-26	0.82	0.81	0.06	10.75	0.8705	2	1	14	2	23	Beta lactam Genes	1
**Luz19 + Tobramycin**	IMP-1	0	NA	NA	NA	NA	1	0	14	1	23	Beta lactam Genes	NA
**Luz19 + Tobramycin**	KPC-5	NA	NA	NA	NA	NA	1	1	14	0	23	Beta lactam Genes	NA
**Luz19 + Tobramycin**	mexA	1	NA	NA	NA	NA	14	14	14	23	23	Efflux pumps Other Genes	NA
**Luz19 + Tobramycin**	mexE	1	NA	NA	NA	NA	14	14	14	23	23	Efflux pumps Other Genes	NA
**Luz19 + Tobramycin**	mexX	0.87	0.18	0.02	1.79	0.1443	9	9	14	17	23	Efflux pumps Other Genes	0.9381

bioRxiv preprint doi: https://doi.org/10.64898/2026.09.27.754834; this version posted September 29, 2026. The copyright holder for this preprint (which was not certified by peer review) is the author/funder, who has granted bioRxiv a license to display the preprint in perpetuity. It is made available under a CC-BY 4.0 International license.

**Luz19 + Tobramycin**	NDM-1	NA	NA	NA	NA	NA	1	2	14	0	23	Beta lactam Genes	NA
**Luz19 + Tobramycin**	OXA-10	4.93	9.72	0.65	146.18	0.1001	2	3	14	1	23	Beta lactam Genes	0.7944
**Luz19 + Tobramycin**	OXA-101	0	NA	NA	NA	NA	1	0	14	1	23	Beta lactam Genes	NA
**Luz19 + Tobramycin**	OXA-2	0.55	0.24	0.02	3.51	0.2992	4	3	14	9	23	Beta lactam Genes	1
**Luz19 + Tobramycin**	OXA-395	0.41	2.87	0.12	71.23	0.5196	3	1	14	4	23	Beta lactam Genes	1
**Luz19 + Tobramycin**	OXA-396	NA	NA	NA	NA	NA	0	0	14	0	23	Beta lactam Genes	NA
**Luz19 + Tobramycin**	OXA-4	4.11	NA	NA	NA	NA	1	5	14	2	23	Beta lactam Genes	NA
**Luz19 + Tobramycin**	OXA-486	4.11	NA	NA	NA	NA	1	5	14	2	23	Beta lactam Genes	NA
**Luz19 + Tobramycin**	OXA-488	1.64	3	0.44	20.59	0.2647	2	3	14	3	23	Beta lactam Genes	1
**Luz19 + Tobramycin**	OXA-494	0	NA	NA	NA	NA	1	0	14	1	23	Beta lactam Genes	NA
**Luz19 + Tobramycin**	OXA-50	0.82	1.65	0.1	28.02	0.7307	3	1	14	2	23	Beta lactam Genes	1
**Luz19 + Tobramycin**	OXA-846	0.73	0.33	0.02	5.94	0.4512	3	4	14	9	23	Beta lactam Genes	1
**Luz19 + Tobramycin**	OXA-848	0	NA	NA	NA	NA	1	0	14	2	23	Beta lactam Genes	NA
**Luz19 + Tobramycin**	OXA-9	0	NA	NA	NA	NA	1	0	14	1	23	Beta lactam Genes	NA
**Luz19 + Tobramycin**	OXA-914	NA	NA	NA	NA	NA	0	0	14	0	23	Beta lactam Genes	NA
**Luz19 + Tobramycin**	OXA-937	NA	NA	NA	NA	NA	0	0	14	0	23	Beta lactam Genes	NA
**Luz19 + Tobramycin**	PAO	0	NA	NA	NA	NA	1	0	14	1	23	Beta lactam Genes	NA
**Luz19 + Tobramycin**	PDC-103	NA	NA	NA	NA	NA	1	1	14	0	23	Beta lactam Genes	NA
**Luz19 + Tobramycin**	PDC-11	3.29	NA	NA	NA	NA	1	2	14	1	23	Beta lactam Genes	NA
**Luz19 + Tobramycin**	PDC-15	0	NA	NA	NA	NA	1	0	14	1	23	Beta lactam Genes	NA
**Luz19 + Tobramycin**	PDC-16	0	NA	NA	NA	NA	1	0	14	2	23	Beta lactam Genes	NA
**Luz19 + Tobramycin**	PDC-173	0	NA	NA	NA	NA	1	0	14	1	23	Beta lactam Genes	NA
**Luz19 + Tobramycin**	PDC-19A	0.41	0.01	0	5.73	0.1417	2	2	14	8	23	Beta lactam Genes	0.9381
**Luz19 + Tobramycin**	PDC-3	2.74	0.17	0	14.04	0.4325	2	5	14	3	23	Beta lactam Genes	1
**Luz19 + Tobramycin**	PDC-34	NA	NA	NA	NA	NA	0	0	14	0	23	Beta lactam Genes	NA
**Luz19 + Tobramycin**	PDC-35	1.64	NA	NA	NA	NA	1	2	14	2	23	Beta lactam Genes	NA
**Luz19 + Tobramycin**	PDC-36	NA	NA	NA	NA	NA	1	1	14	0	23	Beta lactam Genes	NA
**Luz19 + Tobramycin**	PDC-37	1.64	NA	NA	NA	NA	1	1	14	1	23	Beta lactam Genes	NA
**Luz19 + Tobramycin**	PDC-407	NA	NA	NA	NA	NA	0	0	14	0	23	Beta lactam Genes	NA
**Luz19 + Tobramycin**	PDC-5	0	NA	NA	NA	NA	1	0	14	3	23	Beta lactam Genes	NA
**Luz19 + Tobramycin**	PDC-8	NA	NA	NA	NA	NA	0	0	14	0	23	Beta lactam Genes	NA
**Luz19 + Tobramycin**	PME-1	NA	NA	NA	NA	NA	1	2	14	0	23	Beta lactam Genes	NA
**Luz19 + Tobramycin**	qacEdelta1	0.37	0.01	0	4.94	0.1492	3	2	14	9	23	Efflux pumps Other Genes	0.9381
**Luz19 + Tobramycin**	qacG2	0	NA	NA	NA	NA	1	0	14	1	23	Efflux pumps Other Genes	NA

bioRxiv preprint doi: https://doi.org/10.64898/2026.09.27.754834; this version posted September 29, 2026. The copyright holder for this preprint (which was not certified by peer review) is the author/funder, who has granted bioRxiv a license to display the preprint in perpetuity. It is made available under a CC-BY 4.0 International license.

**Luz19 + Tobramycin**	rmtD2	NA	NA	NA	NA	NA	1	2	14	0	23	Aminoglycoside Genes	NA
**Luz19 + Tobramycin**	strA	NA	409643179.16	0	Inf	0.9974	2	3	14	0	23	Aminoglycoside Genes	1
**Luz19 + Tobramycin**	sul1	1.23	0.93	0.12	7.17	0.9456	10	12	14	16	23	Sulfonamides Genes	1
**Luz19 + Tobramycin**	tet(C)	NA	NA	NA	NA	NA	1	2	14	0	23	Tetracyclines Genes	NA
**Luz19 + Tobramycin**	tet(G)	6.57	6.8	0.5	91.89	0.149	3	8	14	2	23	Tetracyclines Genes	0.9381
**Luz19 + Tobramycin**	tet(R)	3.29	NA	NA	NA	NA	1	2	14	1	23	Tetracyclines Genes	NA
**Luz19 + Tobramycin**	-VEB 9.00	3.29	NA	NA	NA	NA	1	2	14	1	23	Beta lactam Genes	NA
**Luz19 + Tobramycin**	VIM-11	NA	NA	NA	NA	NA	1	1	14	0	23	Beta lactam Genes	NA
**Luz19 + Tobramycin**	VIM-2	2.05	0.11	0	6.39	0.2824	3	5	14	4	23	Beta lactam Genes	1
**PAML31.1 + Amikacin**	aac(3)-Id	NA	NA	NA	NA	NA	1	5	18	0	3	Aminoglycoside Genes	NA
**PAML31.1 + Amikacin**	aac(6')-29B	0	NA	NA	NA	NA	1	0	18	1	3	Aminoglycoside Genes	NA
**PAML31.1 + Amikacin**	aac(6')-33	1.5	0	0	117E+10	0.2228	3	9	18	1	3	Aminoglycoside Genes	1
**PAML31.1 + Amikacin**	aac(6')-Ib-G	NA	0	0	1.3E+91	0.9041	2	6	18	0	3	Aminoglycoside Genes	1
**PAML31.1 + Amikacin**	aac(6')-IB3	NA	NA	NA	NA	NA	1	1	18	0	3	Aminoglycoside Genes	NA
**PAML31.1 + Amikacin**	aac(6')-IIa	NA	NA	NA	NA	NA	1	1	18	0	3	Aminoglycoside Genes	NA
**PAML31.1 + Amikacin**	aac(6')-IIc	NA	NA	NA	NA	NA	0	0	18	0	3	Aminoglycoside Genes	NA
**PAML31.1 + Amikacin**	aac(6')-Il	NA	1.972E+40	0	Inf	1	3	9	18	0	3	Aminoglycoside Genes	1
**PAML31.1 + Amikacin**	aac(6')-SET	NA	NA	NA	NA	NA	0	0	18	0	3	Aminoglycoside Genes	NA
**PAML31.1 + Amikacin**	aadA1	NA	1.038E+50	0	Inf	1	3	12	18	0	3	Aminoglycoside Genes	1
**PAML31.1 + Amikacin**	aadA2	NA	NA	NA	NA	NA	1	5	18	0	3	Aminoglycoside Genes	NA
**PAML31.1 + Amikacin**	aadA6	0	NA	NA	NA	NA	1	0	18	1	3	Aminoglycoside Genes	NA
**PAML31.1 + Amikacin**	aadA7	NA	NA	NA	NA	NA	0	0	18	0	3	Aminoglycoside Genes	NA
**PAML31.1 + Amikacin**	ant(2")-Ia	NA	6.992E+53	0	Inf	1	2	4	18	0	3	Aminoglycoside Genes	1
**PAML31.1 + Amikacin**	aph(3')-Ia	NA	NA	NA	NA	NA	1	1	18	0	3	Aminoglycoside Genes	NA
**PAML31.1 + Amikacin**	aph(3')-Ib	NA	NA	NA	NA	NA	1	1	18	0	3	Aminoglycoside Genes	NA
**PAML31.1 + Amikacin**	aph(3')-IIb	0.94	0	0	1.9E+32	0.4343	8	17	18	3	3	Aminoglycoside Genes	1
**PAML31.1 + Amikacin**	aph(6)-Id	NA	NA	NA	NA	NA	1	1	18	0	3	Aminoglycoside Genes	NA
**PAML31.1 + Amikacin**	bcr1	1	NA	NA	NA	NA	8	18	18	3	3	Phenicols Bicyclomycins Genes	NA
**PAML31.1 + Amikacin**	catB7	0	0	0	Inf	1	2	0	18	2	3	Phenicols Bicyclomycins Genes	1
**PAML31.1 + Amikacin**	cmlA5	NA	NA	NA	NA	NA	1	1	18	0	3	Phenicols Bicyclomycins Genes	NA
**PAML31.1 + Amikacin**	cmlA6	NA	NA	NA	NA	NA	1	5	18	0	3	Phenicols Bicyclomycins Genes	NA
**PAML31.1 + Amikacin**	cmlB	NA	NA	NA	NA	NA	0	0	18	0	3	Phenicols Bicyclomycins Genes	NA
**PAML31.1 + Amikacin**	crpP	NA	NA	NA	NA	NA	1	1	18	0	3	Quinolone Genes	NA
**PAML31.1 + Amikacin**	dfrB2	NA	NA	NA	NA	NA	1	3	18	0	3	Trimethoprim Genes	NA
**PAML31.1 + Amikacin**	dfrB5	NA	3.186E+13	0	Inf	1	2	6	18	0	3	Trimethoprim Genes	1

bioRxiv preprint doi: https://doi.org/10.64898/2026.09.27.754834; this version posted September 29, 2026. The copyright holder for this preprint (which was not certified by peer review) is the author/funder, who has granted bioRxiv a license to display the preprint in perpetuity. It is made available under a CC-BY 4.0 International license.

**PAML31.1 + Amikacin**	floR2	NA	3.187E+11	0	Inf	0.9911	3	8	18	0	3	Phenicols Bicyclomycins Genes	1
**PAML31.1 + Amikacin**	fosA	1.5	9.937E+8	0	Inf	1	7	18	18	2	3	Fosfomycin Genes	1
**PAML31.1 + Amikacin**	GES-1	NA	NA	NA	NA	NA	1	1	18	0	3	Beta lactam Genes	NA
**PAML31.1 + Amikacin**	GES-19	NA	1431.38	0	Inf	1	2	8	18	0	3	Beta lactam Genes	1
**PAML31.1 + Amikacin**	GES-20	1.33	0	0	1.3E+12	0.6449	3	8	18	1	3	Beta lactam Genes	1
**PAML31.1 + Amikacin**	GES-26	0.17	0	0	2.03E+11	0.2307	2	1	18	1	3	Beta lactam Genes	1
**PAML31.1 + Amikacin**	IMP-1	NA	NA	NA	NA	NA	0	0	18	0	3	Beta lactam Genes	NA
**PAML31.1 + Amikacin**	KPC-5	NA	NA	NA	NA	NA	0	0	18	0	3	Beta lactam Genes	NA
**PAML31.1 + Amikacin**	mexA	1	NA	NA	NA	NA	8	18	18	3	3	Efflux pumps Other Genes	NA
**PAML31.1 + Amikacin**	mexE	1	NA	NA	NA	NA	8	18	18	3	3	Efflux pumps Other Genes	NA
**PAML31.1 + Amikacin**	mexX	1.17	0	0	8.39E+11	0.4934	5	14	18	2	3	Efflux pumps Other Genes	1
**PAML31.1 + Amikacin**	NDM-1	NA	NA	NA	NA	NA	1	2	18	0	3	Beta lactam Genes	NA
**PAML31.1 + Amikacin**	OXA-10	NA	6.992E+53	0	Inf	1	2	4	18	0	3	Beta lactam Genes	1
**PAML31.1 + Amikacin**	OXA-101	NA	NA	NA	NA	NA	0	0	18	0	3	Beta lactam Genes	NA
**PAML31.1 + Amikacin**	OXA-2	NA	0	0	4.1E+91	0.9106	2	9	18	0	3	Beta lactam Genes	1
**PAML31.1 + Amikacin**	OXA-395	NA	NA	NA	NA	NA	1	1	18	0	3	Beta lactam Genes	NA
**PAML31.1 + Amikacin**	OXA-396	NA	NA	NA	NA	NA	0	0	18	0	3	Beta lactam Genes	NA
**PAML31.1 + Amikacin**	OXA-4	NA	NA	NA	NA	NA	1	5	18	0	3	Beta lactam Genes	NA
**PAML31.1 + Amikacin**	OXA-486	NA	NA	NA	NA	NA	1	5	18	0	3	Beta lactam Genes	NA
**PAML31.1 + Amikacin**	OXA-488	NA	NA	NA	NA	NA	0	0	18	0	3	Beta lactam Genes	NA
**PAML31.1 + Amikacin**	OXA-494	0	NA	NA	NA	NA	1	0	18	1	3	Beta lactam Genes	NA
**PAML31.1 + Amikacin**	OXA-50	0	NA	NA	NA	NA	1	0	18	1	3	Beta lactam Genes	NA
**PAML31.1 + Amikacin**	OXA-846	NA	1.038E+50	0	Inf	1	3	12	18	0	3	Beta lactam Genes	1
**PAML31.1 + Amikacin**	OXA-848	0	NA	NA	NA	NA	1	0	18	1	3	Beta lactam Genes	NA
**PAML31.1 + Amikacin**	OXA-9	NA	NA	NA	NA	NA	0	0	18	0	3	Beta lactam Genes	NA
**PAML31.1 + Amikacin**	OXA-914	NA	NA	NA	NA	NA	0	0	18	0	3	Beta lactam Genes	NA
**PAML31.1 + Amikacin**	OXA-937	NA	NA	NA	NA	NA	0	0	18	0	3	Beta lactam Genes	NA
**PAML31.1 + Amikacin**	PAO	NA	NA	NA	NA	NA	0	0	18	0	3	Beta lactam Genes	NA
**PAML31.1 + Amikacin**	PDC-103	NA	NA	NA	NA	NA	0	0	18	0	3	Beta lactam Genes	NA
**PAML31.1 + Amikacin**	PDC-11	NA	NA	NA	NA	NA	1	3	18	0	3	Beta lactam Genes	NA
**PAML31.1 + Amikacin**	PDC-15	0	NA	NA	NA	NA	1	0	18	1	3	Beta lactam Genes	NA
**PAML31.1 + Amikacin**	PDC-16	0	NA	NA	NA	NA	1	0	18	1	3	Beta lactam Genes	NA
**PAML31.1 + Amikacin**	PDC-173	0	NA	NA	NA	NA	1	0	18	1	3	Beta lactam Genes	NA

bioRxiv preprint doi: https://doi.org/10.64898/2026.09.27.754834; this version posted September 29, 2026. The copyright holder for this preprint (which was not certified by peer review) is the author/funder, who has granted bioRxiv a license to display the preprint in perpetuity. It is made available under a CC-BY 4.0 International license.

**PAML31.1 + Amikacin**	PDC-19A	NA	0	0	4.1E+91	0.9106	2	9	18	0	3	Beta lactam Genes	1
**PAML31.1 + Amikacin**	PDC-3	NA	NA	NA	NA	NA	1	5	18	0	3	Beta lactam Genes	NA
**PAML31.1 + Amikacin**	PDC-34	NA	NA	NA	NA	NA	0	0	18	0	3	Beta lactam Genes	NA
**PAML31.1 + Amikacin**	PDC-35	NA	NA	NA	NA	NA	0	0	18	0	3	Beta lactam Genes	NA
**PAML31.1 + Amikacin**	PDC-36	NA	NA	NA	NA	NA	1	1	18	0	3	Beta lactam Genes	NA
**PAML31.1 + Amikacin**	PDC-37	NA	NA	NA	NA	NA	0	0	18	0	3	Beta lactam Genes	NA
**PAML31.1 + Amikacin**	PDC-407	NA	NA	NA	NA	NA	0	0	18	0	3	Beta lactam Genes	NA
**PAML31.1 + Amikacin**	PDC-5	NA	NA	NA	NA	NA	0	0	18	0	3	Beta lactam Genes	NA
**PAML31.1 + Amikacin**	PDC-8	NA	NA	NA	NA	NA	0	0	18	0	3	Beta lactam Genes	NA
**PAML31.1 + Amikacin**	PME-1	NA	NA	NA	NA	NA	1	2	18	0	3	Beta lactam Genes	NA
**PAML31.1 + Amikacin**	qacEdelta1	1.5	0	0	1.2E+10	0.2228	3	9	18	1	3	Efflux pumps Other Genes	1
**PAML31.1 + Amikacin**	qacG2	0	NA	NA	NA	NA	1	0	18	1	3	Efflux pumps Other Genes	NA
**PAML31.1 + Amikacin**	rmtD2	NA	NA	NA	NA	NA	1	2	18	0	3	Aminoglycoside Genes	NA
**PAML31.1 + Amikacin**	strA	NA	NA	NA	NA	NA	1	1	18	0	3	Aminoglycoside Genes	NA
**PAML31.1 + Amikacin**	sul1	1.42	17299.54	0	8.1E+33	0.7795	7	17	18	2	3	Sulfonamides Genes	1
**PAML31.1 + Amikacin**	tet(C)	NA	NA	NA	NA	NA	0	0	18	0	3	Tetracyclines Genes	NA
**PAML31.1 + Amikacin**	tet(G)	NA	3.187E+11	0	Inf	0.9911	3	8	18	0	3	Tetracyclines Genes	1
**PAML31.1 + Amikacin**	tet(R)	NA	NA	NA	NA	NA	1	3	18	0	3	Tetracyclines Genes	NA
**PAML31.1 + Amikacin**	-VEB 9.00	NA	NA	NA	NA	NA	1	3	18	0	3	Beta lactam Genes	NA
**PAML31.1 + Amikacin**	VIM-11	NA	NA	NA	NA	NA	1	1	18	0	3	Beta lactam Genes	NA
**PAML31.1 + Amikacin**	VIM-2	0.83	0	0	Inf	1	2	5	18	1	3	Beta lactam Genes	1
**PAML31.1 + Cefepime**	aac(3)-Id	NA	NA	NA	NA	NA	1	7	25	0	4	Aminoglycoside Genes	NA
**PAML31.1 + Cefepime**	aac(6')-29B	0	NA	NA	NA	NA	1	0	25	1	4	Aminoglycoside Genes	NA
**PAML31.1 + Cefepime**	aac(6')-33	1.6	0.34	0	2.02E+2	0.906	3	10	25	1	4	Aminoglycoside Genes	1
**PAML31.1 + Cefepime**	aac(6')-Ib-G	NA	13633824.2	0	Inf	0.988	2	7	25	0	4	Aminoglycoside Genes	1
**PAML31.1 + Cefepime**	aac(6')-IB3	NA	NA	NA	NA	NA	1	1	25	0	4	Aminoglycoside Genes	NA
**PAML31.1 + Cefepime**	aac(6')-IIa	NA	NA	NA	NA	NA	1	1	25	0	4	Aminoglycoside Genes	NA
**PAML31.1 + Cefepime**	aac(6')-IIc	NA	1.37E+18	0	Inf	0.9801	2	2	25	0	4	Aminoglycoside Genes	1
**PAML31.1 + Cefepime**	aac(6')-Il	NA	2645808877.35	0	Inf	0.9978	3	11	25	0	4	Aminoglycoside Genes	1
**PAML31.1 + Cefepime**	aac(6')-SET	NA	NA	NA	NA	NA	1	1	25	0	4	Aminoglycoside Genes	NA
**PAML31.1 + Cefepime**	aadA1	2.08	0	0	0.59	0.0416	4	13	25	1	4	Aminoglycoside Genes	0.7744
**PAML31.1 + Cefepime**	aadA2	NA	NA	NA	NA	NA	1	7	25	0	4	Aminoglycoside Genes	NA
**PAML31.1 + Cefepime**	aadA6	0.08	0.03	0	15.07	0.2672	2	1	25	2	4	Aminoglycoside Genes	1
**PAML31.1 + Cefepime**	aadA7	NA	NA	NA	NA	NA	1	1	25	0	4	Aminoglycoside Genes	NA

bioRxiv preprint doi: https://doi.org/10.64898/2026.09.27.754834; this version posted September 29, 2026. The copyright holder for this preprint (which was not certified by peer review) is the author/funder, who has granted bioRxiv a license to display the preprint in perpetuity. It is made available under a CC-BY 4.0 International license.

**PAML31.1 + Cefepime**	ant(2")-Ia	NA	273833280.34	0	Inf	0.999	2	4	25	0	4	Aminoglycoside Genes	1
**PAML31.1 + Cefepime**	aph(3')-Ia	NA	NA	NA	NA	NA	1	1	25	0	4	Aminoglycoside Genes	NA
**PAML31.1 + Cefepime**	aph(3')-Ib	NA	NA	NA	NA	NA	1	1	25	0	4	Aminoglycoside Genes	NA
**PAML31.1 + Cefepime**	aph(3')-IIb	0.96	0	0	Inf	0.9932	11	24	25	4	4	Aminoglycoside Genes	1
**PAML31.1 + Cefepime**	aph(6)-Id	NA	NA	NA	NA	NA	1	1	25	0	4	Aminoglycoside Genes	NA
**PAML31.1 + Cefepime**	bcr1	0.96	0	0	Inf	0.9925	10	24	25	4	4	Phenicols Bicyclomycins Genes	1
**PAML31.1 + Cefepime**	catB7	0.08	0	0	0.35	0.0428	3	1	25	2	4	Phenicols Bicyclomycins Genes	0.7744
**PAML31.1 + Cefepime**	cmlA5	NA	NA	NA	NA	NA	1	1	25	0	4	Phenicols Bicyclomycins Genes	NA
**PAML31.1 + Cefepime**	cmlA6	NA	NA	NA	NA	NA	1	7	25	0	4	Phenicols Bicyclomycins Genes	NA
**PAML31.1 + Cefepime**	cmlB	NA	NA	NA	NA	NA	1	1	25	0	4	Phenicols Bicyclomycins Genes	NA
**PAML31.1 + Cefepime**	crpP	NA	3.66E+12	0	Inf	0.9757	2	2	25	0	4	Quinolone Genes	1
**PAML31.1 + Cefepime**	dfrB2	NA	NA	NA	NA	NA	1	3	25	0	4	Trimethoprim Genes	NA
**PAML31.1 + Cefepime**	dfrB5	NA	4655314474.29	0	Inf	0.9978	2	8	25	0	4	Trimethoprim Genes	1
**PAML31.1 + Cefepime**	floR2	NA	3878627473.71	0	Inf	0.9979	3	10	25	0	4	Phenicols Bicyclomycins Genes	1
**PAML31.1 + Cefepime**	fosA	0.96	0	0	Inf	0.9925	10	24	25	4	4	Fosfomycin Genes	1
**PAML31.1 + Cefepime**	GES-1	NA	NA	NA	NA	NA	1	1	25	0	4	Beta lactam Genes	NA
**PAML31.1 + Cefepime**	GES-19	NA	286185479.27	0	Inf	0.988	2	9	25	0	4	Beta lactam Genes	1
**PAML31.1 + Cefepime**	GES-20	1.28	0.22	0	2611E+8	0.8542	3	8	25	1	4	Beta lactam Genes	1
**PAML31.1 + Cefepime**	GES-26	0.32	0.05	0	563.82	0.5213	2	2	25	1	4	Beta lactam Genes	1
**PAML31.1 + Cefepime**	IMP-1	NA	NA	NA	NA	NA	1	1	25	0	4	Beta lactam Genes	NA
**PAML31.1 + Cefepime**	KPC-5	NA	NA	NA	NA	NA	1	1	25	0	4	Beta lactam Genes	NA
**PAML31.1 + Cefepime**	mexA	1	NA	NA	NA	NA	11	25	25	4	4	Efflux pumps Other Genes	NA
**PAML31.1 + Cefepime**	mexE	1	NA	NA	NA	NA	11	25	25	4	4	Efflux pumps Other Genes	NA
**PAML31.1 + Cefepime**	mexX	1.52	0.43	0	5072E+7	0.9181	6	19	25	2	4	Efflux pumps Other Genes	1
**PAML31.1 + Cefepime**	NDM-1	NA	NA	NA	NA	NA	1	2	25	0	4	Beta lactam Genes	NA
**PAML31.1 + Cefepime**	OXA-10	NA	273833280.34	0	Inf	0.999	2	4	25	0	4	Beta lactam Genes	1
**PAML31.1 + Cefepime**	OXA-101	NA	NA	NA	NA	NA	1	1	25	0	4	Beta lactam Genes	NA
**PAML31.1 + Cefepime**	OXA-2	1.76	0	0	0.45	0.0378	4	11	25	1	4	Beta lactam Genes	0.7744
**PAML31.1 + Cefepime**	OXA-395	NA	4629E+13	0	Inf	0.9977	2	2	25	0	4	Beta lactam Genes	1
**PAML31.1 + Cefepime**	OXA-396	NA	NA	NA	NA	NA	0	0	25	0	4	Beta lactam Genes	NA
**PAML31.1 + Cefepime**	OXA-4	NA	NA	NA	NA	NA	1	7	25	0	4	Beta lactam Genes	NA
**PAML31.1 + Cefepime**	OXA-486	1.12	0	0	0.09	0.03	2	7	25	1	4	Beta lactam Genes	0.7744
**PAML31.1 + Cefepime**	OXA-488	0.16	NA	NA	NA	NA	1	1	25	1	4	Beta lactam Genes	NA
**PAML31.1 + Cefepime**	OXA-494	0	NA	NA	NA	NA	1	0	25	1	4	Beta lactam Genes	NA

bioRxiv preprint doi: https://doi.org/10.64898/2026.09.27.754834; this version posted September 29, 2026. The copyright holder for this preprint (which was not certified by peer review) is the author/funder, who has granted bioRxiv a license to display the preprint in perpetuity. It is made available under a CC-BY 4.0 International license.

**PAML31.1 + Cefepime**	OXA-50	NA	NA	NA	NA	NA	1	1	25	0	4	Beta lactam Genes	NA
**PAML31.1 + Cefepime**	OXA-846	NA	1551286356.41	0	Inf	0.998	3	13	25	0	4	Beta lactam Genes	1
**PAML31.1 + Cefepime**	OXA-848	0	NA	NA	NA	NA	1	0	25	1	4	Beta lactam Genes	NA
**PAML31.1 + Cefepime**	OXA-9	NA	NA	NA	NA	NA	1	1	25	0	4	Beta lactam Genes	NA
**PAML31.1 + Cefepime**	OXA-914	NA	NA	NA	NA	NA	0	1	25	0	4	Beta lactam Genes	NA
**PAML31.1 + Cefepime**	OXA-937	NA	NA	NA	NA	NA	0	0	25	0	4	Beta lactam Genes	NA
**PAML31.1 + Cefepime**	PAO	NA	NA	NA	NA	NA	0	0	25	0	4	Beta lactam Genes	NA
**PAML31.1 + Cefepime**	PDC-103	NA	NA	NA	NA	NA	1	1	25	0	4	Beta lactam Genes	NA
**PAML31.1 + Cefepime**	PDC-11	NA	NA	NA	NA	NA	1	3	25	0	4	Beta lactam Genes	NA
**PAML31.1 + Cefepime**	PDC-15	0	NA	NA	NA	NA	1	0	25	1	4	Beta lactam Genes	NA
**PAML31.1 + Cefepime**	PDC-16	0	NA	NA	NA	NA	1	0	25	1	4	Beta lactam Genes	NA
**PAML31.1 + Cefepime**	PDC-173	NA	NA	NA	NA	NA	0	0	25	0	4	Beta lactam Genes	NA
**PAML31.1 + Cefepime**	PDC-19A	NA	564492512.24	0	Inf	0.9981	2	10	25	0	4	Beta lactam Genes	1
**PAML31.1 + Cefepime**	PDC-3	1.28	8.33	0.04	1651.4	0.432	3	8	25	1	4	Beta lactam Genes	1
**PAML31.1 + Cefepime**	PDC-34	NA	NA	NA	NA	NA	0	0	25	0	4	Beta lactam Genes	NA
**PAML31.1 + Cefepime**	PDC-35	0.16	NA	NA	NA	NA	1	1	25	1	4	Beta lactam Genes	NA
**PAML31.1 + Cefepime**	PDC-36	NA	NA	NA	NA	NA	1	1	25	0	4	Beta lactam Genes	NA
**PAML31.1 + Cefepime**	PDC-37	NA	NA	NA	NA	NA	0	0	25	0	4	Beta lactam Genes	NA
**PAML31.1 + Cefepime**	PDC-407	NA	NA	NA	NA	NA	0	0	25	0	4	Beta lactam Genes	NA
**PAML31.1 + Cefepime**	PDC-5	NA	NA	NA	NA	NA	0	1	25	0	4	Beta lactam Genes	NA
**PAML31.1 + Cefepime**	PDC-8	NA	NA	NA	NA	NA	0	0	25	0	4	Beta lactam Genes	NA
**PAML31.1 + Cefepime**	PME-1	NA	NA	NA	NA	NA	1	2	25	0	4	Beta lactam Genes	NA
**PAML31.1 + Cefepime**	qacEdelta1	1.6	0.34	0	202E+11	0.906	3	10	25	1	4	Efflux pumps Other Genes	1
**PAML31.1 + Cefepime**	qacG2	0	NA	NA	NA	NA	1	0	25	1	4	Efflux pumps Other Genes	NA
**PAML31.1 + Cefepime**	rmtD2	NA	NA	NA	NA	NA	1	2	25	0	4	Aminoglycoside Genes	NA
**PAML31.1 + Cefepime**	strA	NA	NA	NA	NA	NA	1	1	25	0	4	Aminoglycoside Genes	NA
**PAML31.1 + Cefepime**	sul1	1.17	4.31	0.01	1473.63	0.6234	9	22	25	3	4	Sulfonamides Genes	1
**PAML31.1 + Cefepime**	tet(C)	NA	NA	NA	NA	NA	0	0	25	0	4	Tetracyclines Genes	NA
**PAML31.1 + Cefepime**	tet(G)	NA	3878627473.71	0	Inf	0.9979	3	10	25	0	4	Tetracyclines Genes	1
**PAML31.1 + Cefepime**	tet(R)	NA	NA	NA	NA	NA	1	3	25	0	4	Tetracyclines Genes	NA
**PAML31.1 + Cefepime**	-VEB 9.00	NA	NA	NA	NA	NA	1	3	25	0	4	Beta lactam Genes	NA
**PAML31.1 + Cefepime**	VIM-11	NA	NA	NA	NA	NA	1	1	25	0	4	Beta lactam Genes	NA
**PAML31.1 + Cefepime**	VIM-2	1.28	1111002.38	0.35	3.5E+12	0.0685	3	8	25	1	4	Beta lactam Genes	0.7861

bioRxiv preprint doi: https://doi.org/10.64898/2026.09.27.754834; this version posted September 29, 2026. The copyright holder for this preprint (which was not certified by peer review) is the author/funder, who has granted bioRxiv a license to display the preprint in perpetuity. It is made available under a CC-BY 4.0 International license.

**PAML31.1 + Ceftazidime**	aac(3)-Id	NA	NA	NA	NA	NA	1	6	24	0	4	Aminoglycoside Genes	NA
**PAML31.1 + Ceftazidime**	aac(6')-29B	0	NA	NA	NA	NA	1	0	24	1	4	Aminoglycoside Genes	NA
**PAML31.1 + Ceftazidime**	aac(6')-33	1.67	0.34	0	1869E+7	0.9048	3	10	24	1	4	Aminoglycoside Genes	1
**PAML31.1 + Ceftazidime**	aac(6')-Ib-G	NA	56928564.44	0	Inf	0.988	2	7	24	0	4	Aminoglycoside Genes	1
**PAML31.1 + Ceftazidime**	aac(6')-IB3	NA	NA	NA	NA	NA	1	1	24	0	4	Aminoglycoside Genes	NA
**PAML31.1 + Ceftazidime**	aac(6')-IIa	NA	NA	NA	NA	NA	1	1	24	0	4	Aminoglycoside Genes	NA
**PAML31.1 + Ceftazidime**	aac(6')-IIc	NA	119E+17	0	Inf	0.9986	2	2	24	0	4	Aminoglycoside Genes	1
**PAML31.1 + Ceftazidime**	aac(6')-Il	NA	2454490610.91	0	Inf	0.9979	3	10	24	0	4	Aminoglycoside Genes	1
**PAML31.1 + Ceftazidime**	aac(6')-SET	NA	NA	NA	NA	NA	1	1	24	0	4	Aminoglycoside Genes	NA
**PAML31.1 + Ceftazidime**	aadA1	2.17	0	0	0.22	0.0276	4	13	24	1	4	Aminoglycoside Genes	0.7744
**PAML31.1 + Ceftazidime**	aadA2	NA	NA	NA	NA	NA	1	6	24	0	4	Aminoglycoside Genes	NA
**PAML31.1 + Ceftazidime**	aadA6	0.08	0.03	0	13.67	0.2673	2	1	24	2	4	Aminoglycoside Genes	1
**PAML31.1 + Ceftazidime**	aadA7	NA	NA	NA	NA	NA	1	1	24	0	4	Aminoglycoside Genes	NA
**PAML31.1 + Ceftazidime**	ant(2")-la	NA	181326161.7	0	Inf	0.9948	2	4	24	0	4	Aminoglycoside Genes	1
**PAML31.1 + Ceftazidime**	aph(3')-Ia	NA	NA	NA	NA	NA	1	1	24	0	4	Aminoglycoside Genes	NA
**PAML31.1 + Ceftazidime**	aph(3')-Ib	NA	NA	NA	NA	NA	1	1	24	0	4	Aminoglycoside Genes	NA
**PAML31.1 + Ceftazidime**	aph(3')-IIb	0.96	0	0	Inf	0.9867	11	23	24	4	4	Aminoglycoside Genes	1
**PAML31.1 + Ceftazidime**	aph(6)-Id	NA	NA	NA	NA	NA	1	1	24	0	4	Aminoglycoside Genes	NA
**PAML31.1 + Ceftazidime**	bcr1	0.96	0	0	Inf	0.9944	10	23	24	4	4	Phenicols Bicyclomycins Genes	1
**PAML31.1 + Ceftazidime**	catB7	0.08	0	0	0.33	0.0423	3	1	24	2	4	Phenicols Bicyclomycins Genes	0.7744
**PAML31.1 + Ceftazidime**	cmlA5	NA	NA	NA	NA	NA	1	1	24	0	4	Phenicols Bicyclomycins Genes	NA
**PAML31.1 + Ceftazidime**	cmlA6	NA	NA	NA	NA	NA	1	6	24	0	4	Phenicols Bicyclomycins Genes	NA
**PAML31.1 + Ceftazidime**	cmlB	NA	NA	NA	NA	NA	1	1	24	0	4	Phenicols Bicyclomycins Genes	NA
**PAML31.1 + Ceftazidime**	crpP	NA	2.453E+26	0	Inf	1	2	2	24	0	4	Quinolone Genes	1
**PAML31.1 + Ceftazidime**	dfrB2	NA	NA	NA	NA	NA	1	3	24	0	4	Trimethoprim Genes	NA
**PAML31.1 + Ceftazidime**	dfrB5	NA	4419E+9	0	Inf	0.998	2	7	24	0	4	Trimethoprim Genes	1
**PAML31.1 + Ceftazidime**	floR2	NA	3630229306.07	0	Inf	0.9914	3	9	24	0	4	Phenicols Bicyclomycins Genes	1
**PAML31.1 + Ceftazidime**	fosA	0.96	0	0	Inf	0.9944	10	23	24	4	4	Fosfomycin Genes	1
**PAML31.1 + Ceftazidime**	GES-1	NA	NA	NA	NA	NA	1	1	24	0	4	Beta lactam Genes	NA
**PAML31.1 + Ceftazidime**	GES-19	NA	306518767.32	0	Inf	0.988	2	9	24	0	4	Beta lactam Genes	1
**PAML31.1 + Ceftazidime**	GES-20	1.33	0.22	0	2343680	0.8529	3	8	24	1	4	Beta lactam Genes	1
**PAML31.1 + Ceftazidime**	GES-26	0.33	0.06	0	181.42	0.4894	2	2	24	1	4	Beta lactam Genes	1
**PAML31.1 + Ceftazidime**	IMP-1	NA	NA	NA	NA	NA	1	1	24	0	4	Beta lactam Genes	NA
**PAML31.1 + Ceftazidime**	KPC-5	NA	NA	NA	NA	NA	1	1	24	0	4	Beta lactam Genes	NA

bioRxiv preprint doi: https://doi.org/10.64898/2026.09.27.754834; this version posted September 29, 2026. The copyright holder for this preprint (which was not certified by peer review) is the author/funder, who has granted bioRxiv a license to display the preprint in perpetuity. It is made available under a CC-BY 4.0 International license.

**PAML31.1 + Ceftazidime**	mexA	1	NA	NA	NA	NA	11	24	24	4	4	Efflux pumps Other Genes	NA
**PAML31.1 + Ceftazidime**	mexE	1	NA	NA	NA	NA	11	24	24	4	4	Efflux pumps Other Genes	NA
**PAML31.1 + Ceftazidime**	mexX	1.5	0.45	0	4360660	0.9216	6	18	24	2	4	Efflux pumps Other Genes	1
**PAML31.1 + Ceftazidime**	NDM-1	NA	NA	NA	NA	NA	1	2	24	0	4	Beta lactam Genes	NA
**PAML31.1 + Ceftazidime**	OXA-10	NA	181326161.7	0	Inf	0.9948	2	4	24	0	4	Beta lactam Genes	1
**PAML31.1 + Ceftazidime**	OXA-101	NA	NA	NA	NA	NA	1	1	24	0	4	Beta lactam Genes	NA
**PAML31.1 + Ceftazidime**	OXA-2	1.83	0	0	0.07	0.0166	4	11	24	1	4	Beta lactam Genes	0.7286
**PAML31.1 + Ceftazidime**	OXA-395	NA	3.936E+13	0	Inf	0.9982	2	2	24	0	4	Beta lactam Genes	1
**PAML31.1 + Ceftazidime**	OXA-396	NA	NA	NA	NA	NA	0	0	24	0	4	Beta lactam Genes	NA
**PAML31.1 + Ceftazidime**	OXA-4	NA	NA	NA	NA	NA	1	6	24	0	4	Beta lactam Genes	NA
**PAML31.1 + Ceftazidime**	OXA-486	1	0	0	0.1	0.0307	2	6	24	1	4	Beta lactam Genes	0.7744
**PAML31.1 + Ceftazidime**	OXA-488	0.17	NA	NA	NA	NA	1	1	24	1	4	Beta lactam Genes	NA
**PAML31.1 + Ceftazidime**	OXA-494	0	NA	NA	NA	NA	1	0	24	1	4	Beta lactam Genes	NA
**PAML31.1 + Ceftazidime**	OXA-50	NA	NA	NA	NA	NA	1	1	24	0	4	Beta lactam Genes	NA
**PAML31.1 + Ceftazidime**	OXA-846	NA	1513374161.52	0	Inf	0.998	3	13	24	0	4	Beta lactam Genes	1
**PAML31.1 + Ceftazidime**	OXA-848	0	NA	NA	NA	NA	1	0	24	1	4	Beta lactam Genes	NA
**PAML31.1 + Ceftazidime**	OXA-9	NA	NA	NA	NA	NA	1	1	24	0	4	Beta lactam Genes	NA
**PAML31.1 + Ceftazidime**	OXA-914	NA	NA	NA	NA	NA	0	1	24	0	4	Beta lactam Genes	NA
**PAML31.1 + Ceftazidime**	OXA-937	NA	NA	NA	NA	NA	0	0	24	0	4	Beta lactam Genes	NA
**PAML31.1 + Ceftazidime**	PAO	NA	NA	NA	NA	NA	0	0	24	0	4	Beta lactam Genes	NA
**PAML31.1 + Ceftazidime**	PDC-103	NA	NA	NA	NA	NA	1	1	24	0	4	Beta lactam Genes	NA
**PAML31.1 + Ceftazidime**	PDC-11	NA	NA	NA	NA	NA	1	3	24	0	4	Beta lactam Genes	NA
**PAML31.1 + Ceftazidime**	PDC-15	0	NA	NA	NA	NA	1	0	24	1	4	Beta lactam Genes	NA
**PAML31.1 + Ceftazidime**	PDC-16	0	NA	NA	NA	NA	1	0	24	1	4	Beta lactam Genes	NA
**PAML31.1 + Ceftazidime**	PDC-173	NA	NA	NA	NA	NA	0	0	24	0	4	Beta lactam Genes	NA
**PAML31.1 + Ceftazidime**	PDC-19A	NA	797684388.14	0	Inf	0.9902	2	10	24	0	4	Beta lactam Genes	1
**PAML31.1 + Ceftazidime**	PDC-3	1.17	7.45	0.05	1071.28	0.4282	3	7	24	1	4	Beta lactam Genes	1
**PAML31.1 + Ceftazidime**	PDC-34	NA	NA	NA	NA	NA	0	0	24	0	4	Beta lactam Genes	NA
**PAML31.1 + Ceftazidime**	PDC-35	0.17	NA	NA	NA	NA	1	1	24	1	4	Beta lactam Genes	NA
**PAML31.1 + Ceftazidime**	PDC-36	NA	NA	NA	NA	NA	1	1	24	0	4	Beta lactam Genes	NA
**PAML31.1 + Ceftazidime**	PDC-37	NA	NA	NA	NA	NA	0	0	24	0	4	Beta lactam Genes	NA
**PAML31.1 + Ceftazidime**	PDC-407	NA	NA	NA	NA	NA	0	0	24	0	4	Beta lactam Genes	NA
**PAML31.1 + Ceftazidime**	PDC-5	NA	NA	NA	NA	NA	0	1	24	0	4	Beta lactam Genes	NA
**PAML31.1 + Ceftazidime**	PDC-8	NA	NA	NA	NA	NA	0	0	24	0	4	Beta lactam Genes	NA

bioRxiv preprint doi: https://doi.org/10.64898/2026.09.27.754834; this version posted September 29, 2026. The copyright holder for this preprint (which was not certified by peer review) is the author/funder, who has granted bioRxiv a license to display the preprint in perpetuity. It is made available under a CC-BY 4.0 International license.

**PAML31.1 + Ceftazidime**	PME-1	NA	NA	NA	NA	NA	1	2	24	0	4	Beta lactam Genes	NA
**PAML31.1 + Ceftazidime**	qacEdelta1	1.67	0.34	0	1869E+9	0.9048	3	10	24	1	4	Efflux pumps Other Genes	1
**PAML31.1 + Ceftazidime**	qacG2	0	NA	NA	NA	NA	1	0	24	1	4	Efflux pumps Other Genes	NA
**PAML31.1 + Ceftazidime**	rmtD2	NA	NA	NA	NA	NA	1	2	24	0	4	Aminoglycoside Genes	NA
**PAML31.1 + Ceftazidime**	strA	NA	NA	NA	NA	NA	1	1	24	0	4	Aminoglycoside Genes	NA
**PAML31.1 + Ceftazidime**	sul1	1.17	3.8	0.03	539.98	0.5974	9	21	24	3	4	Sulfonamides Genes	1
**PAML31.1 + Ceftazidime**	tet(C)	NA	NA	NA	NA	NA	0	0	24	0	4	Tetracyclines Genes	NA
**PAML31.1 + Ceftazidime**	tet(G)	NA	3630229306.07	0	Inf	0.9914	3	9	24	0	4	Tetracyclines Genes	1
**PAML31.1 + Ceftazidime**	tet(R)	NA	NA	NA	NA	NA	1	3	24	0	4	Tetracyclines Genes	NA
**PAML31.1 + Ceftazidime**	-VEB 9.00	NA	NA	NA	NA	NA	1	3	24	0	4	Beta lactam Genes	NA
**PAML31.1 + Ceftazidime**	VIM-11	NA	NA	NA	NA	NA	1	1	24	0	4	Beta lactam Genes	NA
**PAML31.1 + Ceftazidime**	VIM-2	1.17	1109901.51	0.32	3.80E+9	0.0698	3	7	24	1	4	Beta lactam Genes	0.7861
**PAML31.1 + Ciprofloxacin**	aac(3)-Id	NA	NA	NA	NA	NA	1	7	34	0	12	Aminoglycoside Genes	NA
**PAML31.1 + Ciprofloxacin**	aac(6')-29B	0	NA	NA	NA	NA	1	0	34	1	12	Aminoglycoside Genes	NA
**PAML31.1 + Ciprofloxacin**	aac(6')-33	3.53	3.09	0.13	72.16	0.482	3	10	34	1	12	Aminoglycoside Genes	1
**PAML31.1 + Ciprofloxacin**	aac(6')-Ib-G	NA	129559299.95	0	Inf	0.9709	3	9	34	0	12	Aminoglycoside Genes	1
**PAML31.1 + Ciprofloxacin**	aac(6')-IB3	NA	NA	NA	NA	NA	1	1	34	0	12	Aminoglycoside Genes	NA
**PAML31.1 + Ciprofloxacin**	aac(6')-IIa	NA	NA	NA	NA	NA	1	1	34	0	12	Aminoglycoside Genes	NA
**PAML31.1 + Ciprofloxacin**	aac(6')-IIc	NA	82430349.34	0	Inf	0.9965	2	2	34	0	12	Aminoglycoside Genes	1
**PAML31.1 + Ciprofloxacin**	aac(6')-Il	NA	3854724354.42	0	Inf	0.9981	3	11	34	0	12	Aminoglycoside Genes	1
**PAML31.1 + Ciprofloxacin**	aac(6')-SET	NA	NA	NA	NA	NA	1	1	34	0	12	Aminoglycoside Genes	NA
**PAML31.1 + Ciprofloxacin**	aadA1	4.59	10.27	0.58	181.04	0.1115	4	13	34	1	12	Aminoglycoside Genes	0.8434
**PAML31.1 + Ciprofloxacin**	aadA2	NA	NA	NA	NA	NA	1	7	34	0	12	Aminoglycoside Genes	NA
**PAML31.1 + Ciprofloxacin**	aadA6	0.53	0.48	0.03	7.77	0.606	2	3	34	2	12	Aminoglycoside Genes	1
**PAML31.1 + Ciprofloxacin**	aadA7	NA	NA	NA	NA	NA	1	1	34	0	12	Aminoglycoside Genes	NA
**PAML31.1 + Ciprofloxacin**	ant(2")-la	1.76	3.37	0.1	109.8	0.4947	4	5	34	1	12	Aminoglycoside Genes	1
**PAML31.1 + Ciprofloxacin**	aph(3')-Ia	NA	NA	NA	NA	NA	1	1	34	0	12	Aminoglycoside Genes	NA
**PAML31.1 + Ciprofloxacin**	aph(3')-Ib	NA	NA	NA	NA	NA	1	1	34	0	12	Aminoglycoside Genes	NA
**PAML31.1 + Ciprofloxacin**	aph(3')-IIb	1.13	2.87	0.21	39.19	0.4297	17	32	34	10	12	Aminoglycoside Genes	1
**PAML31.1 + Ciprofloxacin**	aph(6)-Id	NA	65714444.11	0	Inf	0.9978	2	3	34	0	12	Aminoglycoside Genes	1
**PAML31.1 + Ciprofloxacin**	bcr1	0.97	0	0	Inf	0.9975	18	33	34	12	12	Phenicols Bicyclomycins Genes	1
**PAML31.1 + Ciprofloxacin**	catB7	0.3	0.09	0.01	1.45	0.0886	8	6	34	7	12	Phenicols Bicyclomycins Genes	0.7944

bioRxiv preprint doi: https://doi.org/10.64898/2026.09.27.754834; this version posted September 29, 2026. The copyright holder for this preprint (which was not certified by peer review) is the author/funder, who has granted bioRxiv a license to display the preprint in perpetuity. It is made available under a CC-BY 4.0 International license.

**PAML31.1 + Ciprofloxacin**	cmlA5	NA	NA	NA	NA	NA	1	1	34	0	12	Phenicols Bicyclomycins Genes	NA
**PAML31.1 + Ciprofloxacin**	cmlA6	NA	NA	NA	NA	NA	1	7	34	0	12	Phenicols Bicyclomycins Genes	NA
**PAML31.1 + Ciprofloxacin**	cmlB	NA	NA	NA	NA	NA	1	1	34	0	12	Phenicols Bicyclomycins Genes	NA
**PAML31.1 + Ciprofloxacin**	crpP	NA	4136117.13	0	Inf	0.9956	2	2	34	0	12	Quinolone Genes	1
**PAML31.1 + Ciprofloxacin**	dfrB2	NA	NA	NA	NA	NA	1	3	34	0	12	Trimethoprim Genes	NA
**PAML31.1 + Ciprofloxacin**	dfrB5	NA	5521772752.43	0	Inf	0.9983	2	8	34	0	12	Trimethoprim Genes	1
**PAML31.1 + Ciprofloxacin**	floR2	NA	4495348657.26	0	Inf	0.9982	3	10	34	0	12	Phenicols Bicyclomycins Genes	1
**PAML31.1 + Ciprofloxacin**	fosA	1.16	3.76	0.17	83.86	0.4025	16	33	34	10	12	Fosfomycin Genes	1
**PAML31.1 + Ciprofloxacin**	GES-1	NA	NA	NA	NA	NA	1	1	34	0	12	Beta lactam Genes	NA
**PAML31.1 + Ciprofloxacin**	GES-19	NA	242621996.5	0	Inf	0.9974	2	9	34	0	12	Beta lactam Genes	1
**PAML31.1 + Ciprofloxacin**	GES-20	2.82	1.8	0.09	35.71	0.7003	3	8	34	1	12	Beta lactam Genes	1
**PAML31.1 + Ciprofloxacin**	GES-26	0.71	0.34	0.01	10.71	0.5411	2	2	34	1	12	Beta lactam Genes	1
**PAML31.1 + Ciprofloxacin**	IMP-1	NA	NA	NA	NA	NA	1	1	34	0	12	Beta lactam Genes	NA
**PAML31.1 + Ciprofloxacin**	KPC-5	NA	NA	NA	NA	NA	1	1	34	0	12	Beta lactam Genes	NA
**PAML31.1 + Ciprofloxacin**	mexA	1	NA	NA	NA	NA	19	34	34	12	12	Efflux pumps Other Genes	NA
**PAML31.1 + Ciprofloxacin**	mexE	1	NA	NA	NA	NA	19	34	34	12	12	Efflux pumps Other Genes	NA
**PAML31.1 + Ciprofloxacin**	mexX	1.01	0.73	0.08	6.68	0.7824	12	23	34	8	12	Efflux pumps Other Genes	1
**PAML31.1 + Ciprofloxacin**	NDM-1	NA	NA	NA	NA	NA	1	2	34	0	12	Beta lactam Genes	NA
**PAML31.1 + Ciprofloxacin**	OXA-10	NA	60543582.03	0	Inf	0.986	2	4	34	0	12	Beta lactam Genes	1
**PAML31.1 + Ciprofloxacin**	OXA-101	NA	NA	NA	NA	NA	1	1	34	0	12	Beta lactam Genes	NA
**PAML31.1 + Ciprofloxacin**	OXA-2	3.88	4.87	0.17	141.09	0.3566	4	11	34	1	12	Beta lactam Genes	1
**PAML31.1 + Ciprofloxacin**	OXA-395	1.76	18496838.45	34.9	982E+12	0.0128	3	5	34	1	12	Beta lactam Genes	0.7286
**PAML31.1 + Ciprofloxacin**	OXA-396	0	NA	NA	NA	NA	1	0	34	1	12	Beta lactam Genes	NA
**PAML31.1 + Ciprofloxacin**	OXA-4	NA	NA	NA	NA	NA	1	7	34	0	12	Beta lactam Genes	NA
**PAML31.1 + Ciprofloxacin**	OXA-486	2.47	7.08	0.14	351.63	0.326	2	7	34	1	12	Beta lactam Genes	1
**PAML31.1 + Ciprofloxacin**	OXA-488	0.47	0.38	0.04	3.96	0.4214	3	4	34	3	12	Beta lactam Genes	1
**PAML31.1 + Ciprofloxacin**	OXA-494	0.35	NA	NA	NA	NA	1	1	34	1	12	Beta lactam Genes	NA
**PAML31.1 + Ciprofloxacin**	OXA-50	0.09	0.07	0	1.07	0.0555	5	1	34	4	12	Beta lactam Genes	0.7861
**PAML31.1 + Ciprofloxacin**	OXA-846	NA	706021643.66	0	Inf	0.9968	3	13	34	0	12	Beta lactam Genes	1
**PAML31.1 + Ciprofloxacin**	OXA-848	0.35	NA	NA	NA	NA	1	1	34	1	12	Beta lactam Genes	NA
**PAML31.1 + Ciprofloxacin**	OXA-9	NA	NA	NA	NA	NA	1	1	34	0	12	Beta lactam Genes	NA
**PAML31.1 + Ciprofloxacin**	OXA-914	NA	NA	NA	NA	NA	0	1	34	0	12	Beta lactam Genes	NA

bioRxiv preprint doi: https://doi.org/10.64898/2026.09.27.754834; this version posted September 29, 2026. The copyright holder for this preprint (which was not certified by peer review) is the author/funder, who has granted bioRxiv a license to display the preprint in perpetuity. It is made available under a CC-BY 4.0 International license.

**PAML31.1 + Ciprofloxacin**	OXA-937	0.35	NA	NA	NA	NA	1	1	34	1	12	Beta lactam Genes	NA
**PAML31.1 + Ciprofloxacin**	PAO	0	0	0	Inf	0.9864	2	0	34	2	12	Beta lactam Genes	1
**PAML31.1 + Ciprofloxacin**	PDC-103	NA	NA	NA	NA	NA	1	1	34	0	12	Beta lactam Genes	NA
**PAML31.1 + Ciprofloxacin**	PDC-11	NA	NA	NA	NA	NA	1	3	34	0	12	Beta lactam Genes	NA
**PAML31.1 + Ciprofloxacin**	PDC-15	0	NA	NA	NA	NA	1	0	34	1	12	Beta lactam Genes	NA
**PAML31.1 + Ciprofloxacin**	PDC-16	0.35	NA	NA	NA	NA	1	1	34	1	12	Beta lactam Genes	NA
**PAML31.1 + Ciprofloxacin**	PDC-173	0	NA	NA	NA	NA	1	0	34	1	12	Beta lactam Genes	NA
**PAML31.1 + Ciprofloxacin**	PDC-19A	NA	433879753.02	0	Inf	0.9972	2	10	34	0	12	Beta lactam Genes	1
**PAML31.1 + Ciprofloxacin**	PDC-3	1.59	6.01	0.27	131.75	0.2549	4	9	34	2	12	Beta lactam Genes	1
**PAML31.1 + Ciprofloxacin**	PDC-34	0	NA	NA	NA	NA	1	0	34	1	12	Beta lactam Genes	NA
**PAML31.1 + Ciprofloxacin**	PDC-35	1.06	NA	NA	NA	NA	1	3	34	1	12	Beta lactam Genes	NA
**PAML31.1 + Ciprofloxacin**	PDC-36	NA	NA	NA	NA	NA	1	1	34	0	12	Beta lactam Genes	NA
**PAML31.1 + Ciprofloxacin**	PDC-37	0.35	NA	NA	NA	NA	1	1	34	1	12	Beta lactam Genes	NA
**PAML31.1 + Ciprofloxacin**	PDC-407	NA	NA	NA	NA	NA	0	1	34	0	12	Beta lactam Genes	NA
**PAML31.1 + Ciprofloxacin**	PDC-5	1.41	NA	NA	NA	NA	1	4	34	1	12	Beta lactam Genes	NA
**PAML31.1 + Ciprofloxacin**	PDC-8	0	0	0	Inf	0.9928	2	0	34	2	12	Beta lactam Genes	1
**PAML31.1 + Ciprofloxacin**	PME-1	NA	NA	NA	NA	NA	1	2	34	0	12	Beta lactam Genes	NA
**PAML31.1 + Ciprofloxacin**	qacEdelta1	3.53	3.09	0.13	72.16	0.482	3	10	34	1	12	Efflux pumps Other Genes	1
**PAML31.1 + Ciprofloxacin**	qacG2	0	NA	NA	NA	NA	1	0	34	1	12	Efflux pumps Other Genes	NA
**PAML31.1 + Ciprofloxacin**	rmtD2	NA	NA	NA	NA	NA	1	2	34	0	12	Aminoglycoside Genes	NA
**PAML31.1 + Ciprofloxacin**	strA	NA	65714444.11	0	Inf	0.9978	2	3	34	0	12	Aminoglycoside Genes	1
**PAML31.1 + Ciprofloxacin**	sul1	2.12	4.37	0.77	24.85	0.0962	10	24	34	4	12	Sulfonamides Genes	0.7944
**PAML31.1 + Ciprofloxacin**	tet(C)	NA	NA	NA	NA	NA	1	2	34	0	12	Tetracyclines Genes	NA
**PAML31.1 + Ciprofloxacin**	tet(G)	NA	4495348657.26	0	Inf	0.9982	3	10	34	0	12	Tetracyclines Genes	1
**PAML31.1 + Ciprofloxacin**	tet(R)	NA	NA	NA	NA	NA	1	3	34	0	12	Tetracyclines Genes	NA
**PAML31.1 + Ciprofloxacin**	-VEB 9.00	NA	NA	NA	NA	NA	1	3	34	0	12	Beta lactam Genes	NA
**PAML31.1 + Ciprofloxacin**	VIM-11	NA	NA	NA	NA	NA	1	1	34	0	12	Beta lactam Genes	NA
**PAML31.1 + Ciprofloxacin**	VIM-2	2.82	7.82	0.13	458.67	0.3219	3	8	34	1	12	Beta lactam Genes	1
**PAML31.1 + Doripenem**	aac(3)-Id	NA	NA	NA	NA	NA	1	5	24	0	7	Aminoglycoside Genes	NA
**PAML31.1 + Doripenem**	aac(6')-29B	0	NA	NA	NA	NA	1	0	24	1	7	Aminoglycoside Genes	NA
**PAML31.1 + Doripenem**	aac(6')-33	2.92	8.68	0	1.12E+7	0.7189	3	10	24	1	7	Aminoglycoside Genes	1
**PAML31.1 + Doripenem**	aac(6')-Ib-G	NA	82348281.13	0	Inf	0.9929	2	7	24	0	7	Aminoglycoside Genes	1
**PAML31.1 + Doripenem**	aac(6')-IB3	NA	NA	NA	NA	NA	1	1	24	0	7	Aminoglycoside Genes	NA
**PAML31.1 + Doripenem**	aac(6')-IIa	NA	NA	NA	NA	NA	1	1	24	0	7	Aminoglycoside Genes	NA

bioRxiv preprint doi: https://doi.org/10.64898/2026.09.27.754834; this version posted September 29, 2026. The copyright holder for this preprint (which was not certified by peer review) is the author/funder, who has granted bioRxiv a license to display the preprint in perpetuity. It is made available under a CC-BY 4.0 International license.

**PAML31.1 + Doripenem**	aac(6')-IIc	NA	7804289264.57	0	Inf	0.9875	2	2	24	0	7	Aminoglycoside Genes	1
**PAML31.1 + Doripenem**	aac(6')-Il	NA	13292849506	0	Inf	0.9889	3	9	24	0	7	Aminoglycoside Genes	1
**PAML31.1 + Doripenem**	aac(6')-SET	NA	NA	NA	NA	NA	1	1	24	0	7	Aminoglycoside Genes	NA
**PAML31.1 + Doripenem**	aadA1	3.79	0	0	0.07	0.014	4	13	24	1	7	Aminoglycoside Genes	0.7286
**PAML31.1 + Doripenem**	aadA2	NA	NA	NA	NA	NA	1	5	24	0	7	Aminoglycoside Genes	NA
**PAML31.1 + Doripenem**	aadA6	0.15	0	0	1.27E+11	0.6272	2	1	24	2	7	Aminoglycoside Genes	1
**PAML31.1 + Doripenem**	aadA7	NA	NA	NA	NA	NA	1	1	24	0	7	Aminoglycoside Genes	NA
**PAML31.1 + Doripenem**	ant(2")-la	1.17	0.91	0	32379.77	0.9855	3	4	24	1	7	Aminoglycoside Genes	1
**PAML31.1 + Doripenem**	aph(3')-Ia	NA	NA	NA	NA	NA	1	1	24	0	7	Aminoglycoside Genes	NA
**PAML31.1 + Doripenem**	aph(3')-Ib	NA	NA	NA	NA	NA	1	1	24	0	7	Aminoglycoside Genes	NA
**PAML31.1 + Doripenem**	aph(3')-IIb	1.12	9453644.22	1.46	613E+13	0.0447	13	23	24	6	7	Aminoglycoside Genes	0.7744
**PAML31.1 + Doripenem**	aph(6)-Id	NA	NA	NA	NA	NA	1	1	24	0	7	Aminoglycoside Genes	NA
**PAML31.1 + Doripenem**	bcr1	0.96	0	0	Inf	0.9711	13	23	24	7	7	Phenicols Bicyclomycins Genes	1
**PAML31.1 + Doripenem**	catB7	0.15	0	0	0.02	0.028	5	2	24	4	7	Phenicols Bicyclomycins Genes	0.7744
**PAML31.1 + Doripenem**	cmlA5	NA	NA	NA	NA	NA	1	1	24	0	7	Phenicols Bicyclomycins Genes	NA
**PAML31.1 + Doripenem**	cmlA6	NA	NA	NA	NA	NA	1	5	24	0	7	Phenicols Bicyclomycins Genes	NA
**PAML31.1 + Doripenem**	cmlB	NA	NA	NA	NA	NA	1	1	24	0	7	Phenicols Bicyclomycins Genes	NA
**PAML31.1 + Doripenem**	crpP	NA	154170683231	0	Inf	0.9994	2	2	24	0	7	Quinolone Genes	1
**PAML31.1 + Doripenem**	dfrB2	NA	NA	NA	NA	NA	1	3	24	0	7	Trimethoprim Genes	NA
**PAML31.1 + Doripenem**	dfrB5	NA	5520989481.3	0	Inf	0.9939	2	6	24	0	7	Trimethoprim Genes	1
**PAML31.1 + Doripenem**	floR2	NA	6776175604.25	0	Inf	0.9953	3	8	24	0	7	Phenicols Bicyclomycins Genes	1
**PAML31.1 + Doripenem**	fosA	0.96	0	0	Inf	0.9711	13	23	24	7	7	Fosfomycin Genes	1
**PAML31.1 + Doripenem**	GES-1	NA	NA	NA	NA	NA	1	1	24	0	7	Beta lactam Genes	NA
**PAML31.1 + Doripenem**	GES-19	NA	301364540.34	0	Inf	0.9924	2	9	24	0	7	Beta lactam Genes	1
**PAML31.1 + Doripenem**	GES-20	2.33	3.15	0	66821.88	0.8216	3	8	24	1	7	Beta lactam Genes	1
**PAML31.1 + Doripenem**	GES-26	0.58	0.58	0	27624.24	0.9222	2	2	24	1	7	Beta lactam Genes	1
**PAML31.1 + Doripenem**	IMP-1	NA	NA	NA	NA	NA	1	1	24	0	7	Beta lactam Genes	NA
**PAML31.1 + Doripenem**	KPC-5	NA	NA	NA	NA	NA	1	1	24	0	7	Beta lactam Genes	NA
**PAML31.1 + Doripenem**	mexA	1	NA	NA	NA	NA	14	24	24	7	7	Efflux pumps Other Genes	NA
**PAML31.1 + Doripenem**	mexE	1	NA	NA	NA	NA	14	24	24	7	7	Efflux pumps Other Genes	NA
**PAML31.1 + Doripenem**	mexX	1.05	0.67	0	6935.07	0.9335	9	18	24	5	7	Efflux pumps Other Genes	1
**PAML31.1 + Doripenem**	NDM-1	NA	NA	NA	NA	NA	1	2	24	0	7	Beta lactam Genes	NA
**PAML31.1 + Doripenem**	OXA-10	NA	385493286.02	0	Inf	0.9991	2	4	24	0	7	Beta lactam Genes	1
**PAML31.1 + Doripenem**	OXA-101	NA	NA	NA	NA	NA	1	1	24	0	7	Beta lactam Genes	NA

bioRxiv preprint doi: https://doi.org/10.64898/2026.09.27.754834; this version posted September 29, 2026. The copyright holder for this preprint (which was not certified by peer review) is the author/funder, who has granted bioRxiv a license to display the preprint in perpetuity. It is made available under a CC-BY 4.0 International license.

**PAML31.1 + Doripenem**	OXA-2	3.21	0	0	0.08	0.0154	4	11	24	1	7	Beta lactam Genes	0.7286
**PAML31.1 + Doripenem**	OXA-395	NA	1.019E+20	0	Inf	0.9873	3	3	24	0	7	Beta lactam Genes	1
**PAML31.1 + Doripenem**	OXA-396	NA	NA	NA	NA	NA	0	0	24	0	7	Beta lactam Genes	NA
**PAML31.1 + Doripenem**	OXA-4	NA	NA	NA	NA	NA	1	5	24	0	7	Beta lactam Genes	NA
**PAML31.1 + Doripenem**	OXA-486	1.46	3.68	0	9.12E+9	0.9061	2	5	24	1	7	Beta lactam Genes	1
**PAML31.1 + Doripenem**	OXA-488	0.15	0	0	0.81	0.0474	2	1	24	2	7	Beta lactam Genes	0.7744
**PAML31.1 + Doripenem**	OXA-494	0	NA	NA	NA	NA	1	0	24	2	7	Beta lactam Genes	NA
**PAML31.1 + Doripenem**	OXA-50	0.29	0.23	0	24773.71	0.8013	2	1	24	1	7	Beta lactam Genes	1
**PAML31.1 + Doripenem**	OXA-846	NA	4687932384.74	0	Inf	0.997	3	13	24	0	7	Beta lactam Genes	1
**PAML31.1 + Doripenem**	OXA-848	0	NA	NA	NA	NA	1	0	24	1	7	Beta lactam Genes	NA
**PAML31.1 + Doripenem**	OXA-9	NA	NA	NA	NA	NA	1	1	24	0	7	Beta lactam Genes	NA
**PAML31.1 + Doripenem**	OXA-914	NA	NA	NA	NA	NA	0	1	24	0	7	Beta lactam Genes	NA
**PAML31.1 + Doripenem**	OXA-937	NA	NA	NA	NA	NA	0	0	24	0	7	Beta lactam Genes	NA
**PAML31.1 + Doripenem**	PAO	0	NA	NA	NA	NA	1	0	24	1	7	Beta lactam Genes	NA
**PAML31.1 + Doripenem**	PDC-103	NA	NA	NA	NA	NA	1	1	24	0	7	Beta lactam Genes	NA
**PAML31.1 + Doripenem**	PDC-11	NA	NA	NA	NA	NA	1	3	24	0	7	Beta lactam Genes	NA
**PAML31.1 + Doripenem**	PDC-15	0	NA	NA	NA	NA	1	0	24	2	7	Beta lactam Genes	NA
**PAML31.1 + Doripenem**	PDC-16	0	NA	NA	NA	NA	1	0	24	1	7	Beta lactam Genes	NA
**PAML31.1 + Doripenem**	PDC-173	NA	NA	NA	NA	NA	0	0	24	0	7	Beta lactam Genes	NA
**PAML31.1 + Doripenem**	PDC-19A	NA	6435080018.75	0	Inf	0.998	2	10	24	0	7	Beta lactam Genes	1
**PAML31.1 + Doripenem**	PDC-3	1.75	15.15	0	736E+8	0.7293	3	6	24	1	7	Beta lactam Genes	1
**PAML31.1 + Doripenem**	PDC-34	NA	NA	NA	NA	NA	0	0	24	0	7	Beta lactam Genes	NA
**PAML31.1 + Doripenem**	PDC-35	0.29	NA	NA	NA	NA	1	1	24	1	7	Beta lactam Genes	NA
**PAML31.1 + Doripenem**	PDC-36	NA	NA	NA	NA	NA	1	1	24	0	7	Beta lactam Genes	NA
**PAML31.1 + Doripenem**	PDC-37	0	NA	NA	NA	NA	1	0	24	1	7	Beta lactam Genes	NA
**PAML31.1 + Doripenem**	PDC-407	NA	NA	NA	NA	NA	0	0	24	0	7	Beta lactam Genes	NA
**PAML31.1 + Doripenem**	PDC-5	NA	NA	NA	NA	NA	1	2	24	0	7	Beta lactam Genes	NA
**PAML31.1 + Doripenem**	PDC-8	NA	NA	NA	NA	NA	0	0	24	0	7	Beta lactam Genes	NA
**PAML31.1 + Doripenem**	PME-1	NA	NA	NA	NA	NA	1	2	24	0	7	Beta lactam Genes	NA
**PAML31.1 + Doripenem**	qacEdelta1	2.92	8.68	0	1122550	0.7189	3	10	24	1	7	Efflux pumps Other Genes	1
**PAML31.1 + Doripenem**	qacG2	0	NA	NA	NA	NA	1	0	24	1	7	Efflux pumps Other Genes	NA
**PAML31.1 + Doripenem**	rmtD2	NA	NA	NA	NA	NA	1	2	24	0	7	Aminoglycoside Genes	NA
**PAML31.1 + Doripenem**	strA	NA	NA	NA	NA	NA	1	1	24	0	7	Aminoglycoside Genes	NA
**PAML31.1 + Doripenem**	sul1	1.46	7.25	0.02	2406.29	0.5035	10	20	24	4	7	Sulfonamides Genes	1

bioRxiv preprint doi: https://doi.org/10.64898/2026.09.27.754834; this version posted September 29, 2026. The copyright holder for this preprint (which was not certified by peer review) is the author/funder, who has granted bioRxiv a license to display the preprint in perpetuity. It is made available under a CC-BY 4.0 International license.

**PAML31.1 + Doripenem**	tet(C)	NA	NA	NA	NA	NA	0	0	24	0	7	Tetracyclines Genes	NA
**PAML31.1 + Doripenem**	tet(G)	NA	6776175604.25	0	Inf	0.9953	3	8	24	0	7	Tetracyclines Genes	1
**PAML31.1 + Doripenem**	tet(R)	NA	NA	NA	NA	NA	1	3	24	0	7	Tetracyclines Genes	NA
**PAML31.1 + Doripenem**	-VEB 9.00	NA	NA	NA	NA	NA	1	3	24	0	7	Beta lactam Genes	NA
**PAML31.1 + Doripenem**	VIM-11	NA	NA	NA	NA	NA	1	1	24	0	7	Beta lactam Genes	NA
**PAML31.1 + Doripenem**	VIM-2	1.75	580825.7	0.09	3.8E+12	0.0973	3	6	24	1	7	Beta lactam Genes	0.7944
**PAML31.1 + Imipenem**	aac(3)-Id	NA	NA	NA	NA	NA	1	7	33	0	10	Aminoglycoside Genes	NA
**PAML31.1 + Imipenem**	aac(6')-29B	0	NA	NA	NA	NA	1	0	33	1	10	Aminoglycoside Genes	NA
**PAML31.1 + Imipenem**	aac(6')-33	NA	165198320.39	0	Inf	0.9896	2	10	33	0	10	Aminoglycoside Genes	1
**PAML31.1 + Imipenem**	aac(6')-Ib-G	NA	64269598.81	0	Inf	0.9978	3	9	33	0	10	Aminoglycoside Genes	1
**PAML31.1 + Imipenem**	aac(6')-IB3	NA	NA	NA	NA	NA	1	1	33	0	10	Aminoglycoside Genes	NA
**PAML31.1 + Imipenem**	aac(6')-IIa	NA	NA	NA	NA	NA	1	1	33	0	10	Aminoglycoside Genes	NA
**PAML31.1 + Imipenem**	aac(6')-IIc	NA	1106885467.23	0	Inf	0.9988	2	2	33	0	10	Aminoglycoside Genes	1
**PAML31.1 + Imipenem**	aac(6')-Il	NA	5588094635.9	0	Inf	0.9979	3	11	33	0	10	Aminoglycoside Genes	1
**PAML31.1 + Imipenem**	aac(6')-SET	NA	NA	NA	NA	NA	1	1	33	0	10	Aminoglycoside Genes	NA
**PAML31.1 + Imipenem**	aadA1	NA	293653464.78	0	Inf	0.9814	3	13	33	0	10	Aminoglycoside Genes	1
**PAML31.1 + Imipenem**	aadA2	NA	NA	NA	NA	NA	1	7	33	0	10	Aminoglycoside Genes	NA
**PAML31.1 + Imipenem**	aadA6	NA	NA	NA	NA	NA	1	3	33	0	10	Aminoglycoside Genes	NA
**PAML31.1 + Imipenem**	aadA7	NA	NA	NA	NA	NA	1	1	33	0	10	Aminoglycoside Genes	NA
**PAML31.1 + Imipenem**	ant(2")-la	1.52	4.03	0.02	926.19	0.6151	4	5	33	1	10	Aminoglycoside Genes	1
**PAML31.1 + Imipenem**	aph(3')-Ia	NA	NA	NA	NA	NA	1	1	33	0	10	Aminoglycoside Genes	NA
**PAML31.1 + Imipenem**	aph(3')-Ib	NA	NA	NA	NA	NA	1	1	33	0	10	Aminoglycoside Genes	NA
**PAML31.1 + Imipenem**	aph(3')-IIb	1.08	6.16	0.06	586.39	0.4342	15	32	33	9	10	Aminoglycoside Genes	1
**PAML31.1 + Imipenem**	aph(6)-Id	NA	25219604.38	0	Inf	0.9885	2	3	33	0	10	Aminoglycoside Genes	1
**PAML31.1 + Imipenem**	bcr1	0.97	0	0	Inf	0.9981	15	32	33	10	10	Phenicols Bicyclomycins Genes	1
**PAML31.1 + Imipenem**	catB7	0.23	0.02	0	0.31	0.0064	7	6	33	8	10	Phenicols Bicyclomycins Genes	0.7286
**PAML31.1 + Imipenem**	cmlA5	NA	NA	NA	NA	NA	1	1	33	0	10	Phenicols Bicyclomycins Genes	NA
**PAML31.1 + Imipenem**	cmlA6	NA	NA	NA	NA	NA	1	7	33	0	10	Phenicols Bicyclomycins Genes	NA
**PAML31.1 + Imipenem**	cmlB	NA	NA	NA	NA	NA	1	1	33	0	10	Phenicols Bicyclomycins Genes	NA
**PAML31.1 + Imipenem**	crpP	NA	12248562.99	0	Inf	0.9985	2	2	33	0	10	Quinolone Genes	1
**PAML31.1 + Imipenem**	dfrB2	NA	NA	NA	NA	NA	1	3	33	0	10	Trimethoprim Genes	NA
**PAML31.1 + Imipenem**	dfrB5	NA	8336997857.68	0	Inf	0.9982	2	8	33	0	10	Trimethoprim Genes	1
**PAML31.1 + Imipenem**	floR2	NA	6649415560.47	0	Inf	0.9981	3	10	33	0	10	Phenicols Bicyclomycins Genes	1

bioRxiv preprint doi: https://doi.org/10.64898/2026.09.27.754834; this version posted September 29, 2026. The copyright holder for this preprint (which was not certified by peer review) is the author/funder, who has granted bioRxiv a license to display the preprint in perpetuity. It is made available under a CC-BY 4.0 International license.

**PAML31.1 + Imipenem**	fosA	0.97	0	0	Inf	0.9981	15	32	33	10	10	Fosfomycin Genes	1
**PAML31.1 + Imipenem**	GES-1	NA	NA	NA	NA	NA	1	1	33	0	10	Beta lactam Genes	NA
**PAML31.1 + Imipenem**	GES-19	NA	72267790.17	0	Inf	0.9827	2	9	33	0	10	Beta lactam Genes	1
**PAML31.1 + Imipenem**	GES-20	NA	52418742.95	0	Inf	0.983	2	8	33	0	10	Beta lactam Genes	1
**PAML31.1 + Imipenem**	GES-26	NA	NA	NA	NA	NA	1	2	33	0	10	Beta lactam Genes	NA
**PAML31.1 + Imipenem**	IMP-1	NA	NA	NA	NA	NA	1	1	33	0	10	Beta lactam Genes	NA
**PAML31.1 + Imipenem**	KPC-5	NA	NA	NA	NA	NA	1	1	33	0	10	Beta lactam Genes	NA
**PAML31.1 + Imipenem**	mexA	1	NA	NA	NA	NA	16	33	33	10	10	Efflux pumps Other Genes	NA
**PAML31.1 + Imipenem**	mexE	1	NA	NA	NA	NA	16	33	33	10	10	Efflux pumps Other Genes	NA
**PAML31.1 + Imipenem**	mexX	0.87	0.34	0.01	11.12	0.5443	10	23	33	8	10	Efflux pumps Other Genes	1
**PAML31.1 + Imipenem**	NDM-1	NA	NA	NA	NA	NA	1	2	33	0	10	Beta lactam Genes	NA
**PAML31.1 + Imipenem**	OXA-10	NA	105412970.1	0	Inf	0.9898	2	4	33	0	10	Beta lactam Genes	1
**PAML31.1 + Imipenem**	OXA-101	NA	NA	NA	NA	NA	1	1	33	0	10	Beta lactam Genes	NA
**PAML31.1 + Imipenem**	OXA-2	NA	174605066.15	0	Inf	0.9974	3	11	33	0	10	Beta lactam Genes	1
**PAML31.1 + Imipenem**	OXA-395	0.76	78836.33	3.17	1959E+6	0.029	3	5	33	2	10	Beta lactam Genes	0.7744
**PAML31.1 + Imipenem**	OXA-396	NA	NA	NA	NA	NA	0	0	33	0	10	Beta lactam Genes	NA
**PAML31.1 + Imipenem**	OXA-4	NA	NA	NA	NA	NA	1	7	33	0	10	Beta lactam Genes	NA
**PAML31.1 + Imipenem**	OXA-486	2.12	5.96	0.1	366.73	0.396	2	7	33	1	10	Beta lactam Genes	1
**PAML31.1 + Imipenem**	OXA-488	0.91	0.58	0.01	44.8	0.8075	2	3	33	1	10	Beta lactam Genes	1
**PAML31.1 + Imipenem**	OXA-494	0.15	NA	NA	NA	NA	1	1	33	2	10	Beta lactam Genes	NA
**PAML31.1 + Imipenem**	OXA-50	0.2	0.15	0.01	3.52	0.2386	4	2	33	3	10	Beta lactam Genes	1
**PAML31.1 + Imipenem**	OXA-846	NA	293653464.78	0	Inf	0.9814	3	13	33	0	10	Beta lactam Genes	1
**PAML31.1 + Imipenem**	OXA-848	0.3	NA	NA	NA	NA	1	1	33	1	10	Beta lactam Genes	NA
**PAML31.1 + Imipenem**	OXA-9	NA	NA	NA	NA	NA	1	1	33	0	10	Beta lactam Genes	NA
**PAML31.1 + Imipenem**	OXA-914	NA	NA	NA	NA	NA	0	1	33	0	10	Beta lactam Genes	NA
**PAML31.1 + Imipenem**	OXA-937	0	NA	NA	NA	NA	1	0	33	1	10	Beta lactam Genes	NA
**PAML31.1 + Imipenem**	PAO	0	0	0	Inf	0.9975	2	0	33	2	10	Beta lactam Genes	1
**PAML31.1 + Imipenem**	PDC-103	NA	NA	NA	NA	NA	1	1	33	0	10	Beta lactam Genes	NA
**PAML31.1 + Imipenem**	PDC-11	NA	NA	NA	NA	NA	1	3	33	0	10	Beta lactam Genes	NA
**PAML31.1 + Imipenem**	PDC-15	0	NA	NA	NA	NA	1	0	33	2	10	Beta lactam Genes	NA
**PAML31.1 + Imipenem**	PDC-16	0.3	NA	NA	NA	NA	1	1	33	1	10	Beta lactam Genes	NA
**PAML31.1 + Imipenem**	PDC-173	NA	NA	NA	NA	NA	0	0	33	0	10	Beta lactam Genes	NA
**PAML31.1 + Imipenem**	PDC-19A	NA	165198320.39	0	Inf	0.9896	2	10	33	0	10	Beta lactam Genes	1
**PAML31.1 + Imipenem**	PDC-3	1.21	2.56	0.07	93.87	0.6084	4	8	33	2	10	Beta lactam Genes	1
**PAML31.1 + Imipenem**	PDC-34	NA	NA	NA	NA	NA	0	0	33	0	10	Beta lactam Genes	NA

bioRxiv preprint doi: https://doi.org/10.64898/2026.09.27.754834; this version posted September 29, 2026. The copyright holder for this preprint (which was not certified by peer review) is the author/funder, who has granted bioRxiv a license to display the preprint in perpetuity. It is made available under a CC-BY 4.0 International license.

**PAML31.1 + Imipenem**	PDC-35	NA	NA	NA	NA	NA	1	3	33	0	10	Beta lactam Genes	NA
**PAML31.1 + Imipenem**	PDC-36	NA	NA	NA	NA	NA	1	1	33	0	10	Beta lactam Genes	NA
**PAML31.1 + Imipenem**	PDC-37	0	NA	NA	NA	NA	1	0	33	1	10	Beta lactam Genes	NA
**PAML31.1 + Imipenem**	PDC-407	NA	NA	NA	NA	NA	0	1	33	0	10	Beta lactam Genes	NA
**PAML31.1 + Imipenem**	PDC-5	0.61	NA	NA	NA	NA	1	4	33	2	10	Beta lactam Genes	NA
**PAML31.1 + Imipenem**	PDC-8	0.3	NA	NA	NA	NA	1	1	33	1	10	Beta lactam Genes	NA
**PAML31.1 + Imipenem**	PME-1	NA	NA	NA	NA	NA	1	2	33	0	10	Beta lactam Genes	NA
**PAML31.1 + Imipenem**	qacEdelta1	NA	165198320.39	0	Inf	0.9896	2	10	33	0	10	Efflux pumps Other Genes	1
**PAML31.1 + Imipenem**	qacG2	NA	NA	NA	NA	NA	0	0	33	0	10	Efflux pumps Other Genes	NA
**PAML31.1 + Imipenem**	rmtD2	NA	NA	NA	NA	NA	1	2	33	0	10	Aminoglycoside Genes	NA
**PAML31.1 + Imipenem**	strA	NA	25219604.38	0	Inf	0.9885	2	3	33	0	10	Aminoglycoside Genes	1
**PAML31.1 + Imipenem**	sul1	3.64	10.98	1.49	81.05	0.0188	9	24	33	2	10	Sulfonamides Genes	0.7599
**PAML31.1 + Imipenem**	tet(C)	NA	NA	NA	NA	NA	1	2	33	0	10	Tetracyclines Genes	NA
**PAML31.1 + Imipenem**	tet(G)	NA	6649415560.47	0	Inf	0.9981	3	10	33	0	10	Tetracyclines Genes	1
**PAML31.1 + Imipenem**	tet(R)	NA	NA	NA	NA	NA	1	3	33	0	10	Tetracyclines Genes	NA
**PAML31.1 + Imipenem**	-VEB 9.00	NA	NA	NA	NA	NA	1	3	33	0	10	Beta lactam Genes	NA
**PAML31.1 + Imipenem**	VIM-11	NA	NA	NA	NA	NA	1	1	33	0	10	Beta lactam Genes	NA
**PAML31.1 + Imipenem**	VIM-2	2.42	10.93	0.44	268.84	0.1432	3	8	33	1	10	Beta lactam Genes	0.9381
**PAML31.1 + Levofloxacin**	aac(3)-Id	NA	NA	NA	NA	NA	1	7	33	0	13	Aminoglycoside Genes	NA
**PAML31.1 + Levofloxacin**	aac(6')-29B	0	NA	NA	NA	NA	1	0	33	1	13	Aminoglycoside Genes	NA
**PAML31.1 + Levofloxacin**	aac(6')-33	3.94	4.85	0.11	215.25	0.4148	3	10	33	1	13	Aminoglycoside Genes	1
**PAML31.1 + Levofloxacin**	aac(6')-Ib-G	NA	116857122.42	0	Inf	0.9975	3	9	33	0	13	Aminoglycoside Genes	1
**PAML31.1 + Levofloxacin**	aac(6')-IB3	NA	NA	NA	NA	NA	1	1	33	0	13	Aminoglycoside Genes	NA
**PAML31.1 + Levofloxacin**	aac(6')-IIa	NA	NA	NA	NA	NA	1	1	33	0	13	Aminoglycoside Genes	NA
**PAML31.1 + Levofloxacin**	aac(6')-IIc	NA	384410175.63	0	Inf	0.9971	2	2	33	0	13	Aminoglycoside Genes	1
**PAML31.1 + Levofloxacin**	aac(6')-Il	NA	5911956783.23	0	Inf	0.998	3	11	33	0	13	Aminoglycoside Genes	1
**PAML31.1 + Levofloxacin**	aac(6')-SET	NA	NA	NA	NA	NA	1	1	33	0	13	Aminoglycoside Genes	NA
**PAML31.1 + Levofloxacin**	aadA1	5.12	7.41	0.2	274.18	0.277	4	13	33	1	13	Aminoglycoside Genes	1
**PAML31.1 + Levofloxacin**	aadA2	NA	NA	NA	NA	NA	1	7	33	0	13	Aminoglycoside Genes	NA
**PAML31.1 + Levofloxacin**	aadA6	0.59	0.66	0.02	20.51	0.8153	2	3	33	2	13	Aminoglycoside Genes	1
**PAML31.1 + Levofloxacin**	aadA7	NA	NA	NA	NA	NA	1	1	33	0	13	Aminoglycoside Genes	NA
**PAML31.1 + Levofloxacin**	ant(2")-la	1.97	10.67	0.05	2258.77	0.3862	4	5	33	1	13	Aminoglycoside Genes	1
**PAML31.1 + Levofloxacin**	aph(3')-Ia	NA	NA	NA	NA	NA	1	1	33	0	13	Aminoglycoside Genes	NA
**PAML31.1 + Levofloxacin**	aph(3')-Ib	NA	NA	NA	NA	NA	1	1	33	0	13	Aminoglycoside Genes	NA
**PAML31.1 + Levofloxacin**	aph(3')-IIb	1.15	8.28	0.2	343.94	0.2661	17	32	33	11	13	Aminoglycoside Genes	1
**PAML31.1 + Levofloxacin**	aph(6)-Id	NA	217142879	0	Inf	0.9985	2	3	33	0	13	Aminoglycoside Genes	1

bioRxiv preprint doi: https://doi.org/10.64898/2026.09.27.754834; this version posted September 29, 2026. The copyright holder for this preprint (which was not certified by peer review) is the author/funder, who has granted bioRxiv a license to display the preprint in perpetuity. It is made available under a CC-BY 4.0 International license.

**PAML31.1 + Levofloxacin**	bcr1	0.97	0	0	Inf	0.978	18	32	33	13	13	Phenicols Bicyclomycins Genes	1
**PAML31.1 + Levofloxacin**	catB7	0.3	0.06	0	1.53	0.0894	8	6	33	8	13	Phenicols Bicyclomycins Genes	0.7944
**PAML31.1 + Levofloxacin**	cmlA5	NA	NA	NA	NA	NA	1	1	33	0	13	Phenicols Bicyclomycins Genes	NA
**PAML31.1 + Levofloxacin**	cmlA6	NA	NA	NA	NA	NA	1	7	33	0	13	Phenicols Bicyclomycins Genes	NA
**PAML31.1 + Levofloxacin**	cmlB	NA	NA	NA	NA	NA	1	1	33	0	13	Phenicols Bicyclomycins Genes	NA
**PAML31.1 + Levofloxacin**	crpP	NA	10988873	0	Inf	0.9911	2	2	33	0	13	Quinolone Genes	1
**PAML31.1 + Levofloxacin**	dfrB2	NA	NA	NA	NA	NA	1	3	33	0	13	Trimethoprim Genes	NA
**PAML31.1 + Levofloxacin**	dfrB5	NA	8528994150.45	0	Inf	0.9982	2	8	33	0	13	Trimethoprim Genes	1
**PAML31.1 + Levofloxacin**	floR2	NA	6902543416.38	0	Inf	0.9981	3	10	33	0	13	Phenicols Bicyclomycins Genes	1
**PAML31.1 + Levofloxacin**	fosA	1.15	3.59	0.08	155.3	0.5059	16	32	33	11	13	Fosfomycin Genes	1
**PAML31.1 + Levofloxacin**	GES-1	NA	NA	NA	NA	NA	1	1	33	0	13	Beta lactam Genes	NA
**PAML31.1 + Levofloxacin**	GES-19	NA	64093754.78	0	Inf	0.9815	2	9	33	0	13	Beta lactam Genes	1
**PAML31.1 + Levofloxacin**	GES-20	3.15	2.67	0.08	90.3	0.5852	3	8	33	1	13	Beta lactam Genes	1
**PAML31.1 + Levofloxacin**	GES-26	0.79	0.48	0.01	28.98	0.7279	2	2	33	1	13	Beta lactam Genes	1
**PAML31.1 + Levofloxacin**	IMP-1	NA	NA	NA	NA	NA	1	1	33	0	13	Beta lactam Genes	NA
**PAML31.1 + Levofloxacin**	KPC-5	NA	NA	NA	NA	NA	1	1	33	0	13	Beta lactam Genes	NA
**PAML31.1 + Levofloxacin**	mexA	1	NA	NA	NA	NA	19	33	33	13	13	Efflux pumps Other Genes	NA
**PAML31.1 + Levofloxacin**	mexE	1	NA	NA	NA	NA	19	33	33	13	13	Efflux pumps Other Genes	NA
**PAML31.1 + Levofloxacin**	mexX	0.96	0.38	0.02	7.66	0.5279	12	22	33	9	13	Efflux pumps Other Genes	1
**PAML31.1 + Levofloxacin**	NDM-1	NA	NA	NA	NA	NA	1	2	33	0	13	Beta lactam Genes	NA
**PAML31.1 + Levofloxacin**	OXA-10	NA	202893025.41	0	Inf	0.9851	2	4	33	0	13	Beta lactam Genes	1
**PAML31.1 + Levofloxacin**	OXA-101	NA	NA	NA	NA	NA	1	1	33	0	13	Beta lactam Genes	NA
**PAML31.1 + Levofloxacin**	OXA-2	4.33	3.33	0.09	118.33	0.5087	4	11	33	1	13	Beta lactam Genes	1
**PAML31.1 + Levofloxacin**	OXA-395	1.97	36396088.01	71.2	1.9E+13	0.0094	3	5	33	1	13	Beta lactam Genes	0.7286
**PAML31.1 + Levofloxacin**	OXA-396	0	NA	NA	NA	NA	1	0	33	1	13	Beta lactam Genes	NA
**PAML31.1 + Levofloxacin**	OXA-4	NA	NA	NA	NA	NA	1	7	33	0	13	Beta lactam Genes	NA
**PAML31.1 + Levofloxacin**	OXA-486	2.76	8.48	0.18	391.34	0.2742	2	7	33	1	13	Beta lactam Genes	1
**PAML31.1 + Levofloxacin**	OXA-488	0.39	0.14	0	7.03	0.3274	3	3	33	3	13	Beta lactam Genes	1
**PAML31.1 + Levofloxacin**	OXA-494	0.2	NA	NA	NA	NA	1	1	33	2	13	Beta lactam Genes	NA
**PAML31.1 + Levofloxacin**	OXA-50	0.1	0.04	0	2.14	0.115	5	1	33	4	13	Beta lactam Genes	0.8562
**PAML31.1 + Levofloxacin**	OXA-846	NA	856771480.18	0	Inf	0.9965	3	13	33	0	13	Beta lactam Genes	1
**PAML31.1 + Levofloxacin**	OXA-848	0.39	NA	NA	NA	NA	1	1	33	1	13	Beta lactam Genes	NA
**PAML31.1 + Levofloxacin**	OXA-9	NA	NA	NA	NA	NA	1	1	33	0	13	Beta lactam Genes	NA
**PAML31.1 + Levofloxacin**	OXA-914	NA	NA	NA	NA	NA	0	1	33	0	13	Beta lactam Genes	NA
**PAML31.1 + Levofloxacin**	OXA-937	0.39	NA	NA	NA	NA	1	1	33	1	13	Beta lactam Genes	NA
**PAML31.1 + Levofloxacin**	PAO	0	0	0	Inf	0.9977	2	0	33	2	13	Beta lactam Genes	1

bioRxiv preprint doi: https://doi.org/10.64898/2026.09.27.754834; this version posted September 29, 2026. The copyright holder for this preprint (which was not certified by peer review) is the author/funder, who has granted bioRxiv a license to display the preprint in perpetuity. It is made available under a CC-BY 4.0 International license.

**PAML31.1 + Levofloxacin**	PDC-103	NA	NA	NA	NA	NA	1	1	33	0	13	Beta lactam Genes	NA
**PAML31.1 + Levofloxacin**	PDC-11	NA	NA	NA	NA	NA	1	3	33	0	13	Beta lactam Genes	NA
**PAML31.1 + Levofloxacin**	PDC-15	0	NA	NA	NA	NA	1	0	33	2	13	Beta lactam Genes	NA
**PAML31.1 + Levofloxacin**	PDC-16	0.39	NA	NA	NA	NA	1	1	33	1	13	Beta lactam Genes	NA
**PAML31.1 + Levofloxacin**	PDC-173	0	NA	NA	NA	NA	1	0	33	1	13	Beta lactam Genes	NA
**PAML31.1 + Levofloxacin**	PDC-19A	NA	175722014.85	0	Inf	0.9926	2	10	33	0	13	Beta lactam Genes	1
**PAML31.1 + Levofloxacin**	PDC-3	1.77	12.58	0.3	530.28	0.1846	4	9	33	2	13	Beta lactam Genes	1
**PAML31.1 + Levofloxacin**	PDC-34	0	NA	NA	NA	NA	1	0	33	1	13	Beta lactam Genes	NA
**PAML31.1 + Levofloxacin**	PDC-35	1.18	NA	NA	NA	NA	1	3	33	1	13	Beta lactam Genes	NA
**PAML31.1 + Levofloxacin**	PDC-36	NA	NA	NA	NA	NA	1	1	33	0	13	Beta lactam Genes	NA
**PAML31.1 + Levofloxacin**	PDC-37	0	NA	NA	NA	NA	1	0	33	1	13	Beta lactam Genes	NA
**PAML31.1 + Levofloxacin**	PDC-407	NA	NA	NA	NA	NA	0	1	33	0	13	Beta lactam Genes	NA
**PAML31.1 + Levofloxacin**	PDC-5	1.58	NA	NA	NA	NA	1	4	33	1	13	Beta lactam Genes	NA
**PAML31.1 + Levofloxacin**	PDC-8	0	0	0	Inf	0.9967	2	0	33	2	13	Beta lactam Genes	1
**PAML31.1 + Levofloxacin**	PME-1	NA	NA	NA	NA	NA	1	2	33	0	13	Beta lactam Genes	NA
**PAML31.1 + Levofloxacin**	qacEdelta1	3.94	4.85	0.11	215.25	0.4148	3	10	33	1	13	Efflux pumps Other Genes	1
**PAML31.1 + Levofloxacin**	qacG2	0	NA	NA	NA	NA	1	0	33	1	13	Efflux pumps Other Genes	NA
**PAML31.1 + Levofloxacin**	rmtD2	NA	NA	NA	NA	NA	1	2	33	0	13	Aminoglycoside Genes	NA
**PAML31.1 + Levofloxacin**	strA	NA	217142879	0	Inf	0.9985	2	3	33	0	13	Aminoglycoside Genes	1
**PAML31.1 + Levofloxacin**	sul1	2.36	6.36	0.89	45.28	0.0646	10	24	33	4	13	Sulfonamides Genes	0.7861
**PAML31.1 + Levofloxacin**	tet(C)	NA	NA	NA	NA	NA	1	2	33	0	13	Tetracyclines Genes	NA
**PAML31.1 + Levofloxacin**	tet(G)	NA	6902543416.38	0	Inf	0.9981	3	10	33	0	13	Tetracyclines Genes	1
**PAML31.1 + Levofloxacin**	tet(R)	NA	NA	NA	NA	NA	1	3	33	0	13	Tetracyclines Genes	NA
**PAML31.1 + Levofloxacin**	-VEB 9.00	NA	NA	NA	NA	NA	1	3	33	0	13	Beta lactam Genes	NA
**PAML31.1 + Levofloxacin**	VIM-11	NA	NA	NA	NA	NA	1	1	33	0	13	Beta lactam Genes	NA
**PAML31.1 + Levofloxacin**	VIM-2	3.15	7.26	0.14	368.46	0.3225	3	8	33	1	13	Beta lactam Genes	1
**PAML31.1 + Meropenem**	aac(3)-Id	NA	NA	NA	NA	NA	1	5	25	0	8	Aminoglycoside Genes	NA
**PAML31.1 + Meropenem**	aac(6')-29B	0	NA	NA	NA	NA	1	0	25	1	8	Aminoglycoside Genes	NA
**PAML31.1 + Meropenem**	aac(6')-33	3.2	12.98	0.01	11843.57	0.4611	3	10	25	1	8	Aminoglycoside Genes	1
**PAML31.1 + Meropenem**	aac(6')-Ib-G	NA	127161833.99	0	Inf	0.9897	2	7	25	0	8	Aminoglycoside Genes	1
**PAML31.1 + Meropenem**	aac(6')-IB3	NA	NA	NA	NA	NA	1	1	25	0	8	Aminoglycoside Genes	NA
**PAML31.1 + Meropenem**	aac(6')-IIa	NA	NA	NA	NA	NA	1	1	25	0	8	Aminoglycoside Genes	NA
**PAML31.1 + Meropenem**	aac(6')-IIc	NA	3.791E+21	0	Inf	0.8925	2	2	25	0	8	Aminoglycoside Genes	1
**PAML31.1 + Meropenem**	aac(6')-Il	NA	5.196E+10	0	Inf	0.9904	3	9	25	0	8	Aminoglycoside Genes	1
**PAML31.1 + Meropenem**	aac(6')-SET	NA	NA	NA	NA	NA	1	1	25	0	8	Aminoglycoside Genes	NA
**PAML31.1 + Meropenem**	aadA1	4.16	6.21	0.02	2353.71	0.5468	4	13	25	1	8	Aminoglycoside Genes	1

bioRxiv preprint doi: https://doi.org/10.64898/2026.09.27.754834; this version posted September 29, 2026. The copyright holder for this preprint (which was not certified by peer review) is the author/funder, who has granted bioRxiv a license to display the preprint in perpetuity. It is made available under a CC-BY 4.0 International license.

**PAML31.1 + Meropenem**	aadA2	NA	NA	NA	NA	NA	1	5	25	0	8	Aminoglycoside Genes	NA
**PAML31.1 + Meropenem**	aadA6	0.16	0.08	0	157.89	0.5123	2	1	25	2	8	Aminoglycoside Genes	1
**PAML31.1 + Meropenem**	aadA7	NA	NA	NA	NA	NA	1	1	25	0	8	Aminoglycoside Genes	NA
**PAML31.1 + Meropenem**	ant(2")-la	1.28	6.54	0	38545.01	0.6716	3	4	25	1	8	Aminoglycoside Genes	1
**PAML31.1 + Meropenem**	aph(3')-Ia	NA	NA	NA	NA	NA	1	1	25	0	8	Aminoglycoside Genes	NA
**PAML31.1 + Meropenem**	aph(3')-Ib	NA	NA	NA	NA	NA	1	1	25	0	8	Aminoglycoside Genes	NA
**PAML31.1 + Meropenem**	aph(3')-IIb	1.1	10.25	0	82037.12	0.6118	14	24	25	7	8	Aminoglycoside Genes	1
**PAML31.1 + Meropenem**	aph(6)-Id	NA	NA	NA	NA	NA	1	1	25	0	8	Aminoglycoside Genes	NA
**PAML31.1 + Meropenem**	bcr1	0.96	0	0	0	0	14	24	25	8	8	Phenicols Bicyclomycins Genes	0
**PAML31.1 + Meropenem**	catB7	0.19	0	0	0	0.0115	6	3	25	5	8	Phenicols Bicyclomycins Genes	0.7286
**PAML31.1 + Meropenem**	cmlA5	NA	NA	NA	NA	NA	1	1	25	0	8	Phenicols Bicyclomycins Genes	NA
**PAML31.1 + Meropenem**	cmlA6	NA	NA	NA	NA	NA	1	5	25	0	8	Phenicols Bicyclomycins Genes	NA
**PAML31.1 + Meropenem**	cmlB	NA	NA	NA	NA	NA	1	1	25	0	8	Phenicols Bicyclomycins Genes	NA
**PAML31.1 + Meropenem**	crpP	NA	19656042636	0	Inf	0.9827	2	2	25	0	8	Quinolone Genes	1
**PAML31.1 + Meropenem**	dfrB2	NA	NA	NA	NA	NA	1	3	25	0	8	Trimethoprim Genes	NA
**PAML31.1 + Meropenem**	dfrB5	NA	110651562133	0	Inf	0.986	2	6	25	0	8	Trimethoprim Genes	1
**PAML31.1 + Meropenem**	floR2	NA	7525241732.69	0	Inf	0.9911	3	8	25	0	8	Phenicols Bicyclomycins Genes	1
**PAML31.1 + Meropenem**	fosA	1.1	2.08	0	160443.6	0.8985	13	24	25	7	8	Fosfomycin Genes	1
**PAML31.1 + Meropenem**	GES-1	NA	NA	NA	NA	NA	1	1	25	0	8	Beta lactam Genes	NA
**PAML31.1 + Meropenem**	GES-19	NA	322619499.64	0	Inf	0.9983	2	9	25	0	8	Beta lactam Genes	1
**PAML31.1 + Meropenem**	GES-20	2.56	4.14	0.02	926.92	0.6066	3	8	25	1	8	Beta lactam Genes	1
**PAML31.1 + Meropenem**	GES-26	0.64	0.38	0	182.8	0.7594	2	2	25	1	8	Beta lactam Genes	1
**PAML31.1 + Meropenem**	IMP-1	NA	NA	NA	NA	NA	1	1	25	0	8	Beta lactam Genes	NA
**PAML31.1 + Meropenem**	KPC-5	NA	NA	NA	NA	NA	1	1	25	0	8	Beta lactam Genes	NA
**PAML31.1 + Meropenem**	mexA	1	NA	NA	NA	NA	15	25	25	8	8	Efflux pumps Other Genes	NA
**PAML31.1 + Meropenem**	mexE	1	NA	NA	NA	NA	15	25	25	8	8	Efflux pumps Other Genes	NA
**PAML31.1 + Meropenem**	mexX	1.01	0.03	0	169800	0.6508	10	19	25	6	8	Efflux pumps Other Genes	1
**PAML31.1 + Meropenem**	NDM-1	NA	NA	NA	NA	NA	1	2	25	0	8	Beta lactam Genes	NA
**PAML31.1 + Meropenem**	OXA-10	NA	690190988.04	0	Inf	0.999	2	4	25	0	8	Beta lactam Genes	1
**PAML31.1 + Meropenem**	OXA-101	NA	NA	NA	NA	NA	1	1	25	0	8	Beta lactam Genes	NA
**PAML31.1 + Meropenem**	OXA-2	3.52	0	0	0.04	0.0083	4	11	25	1	8	Beta lactam Genes	0.7286
**PAML31.1 + Meropenem**	OXA-395	NA	1.336E+20	0	Inf	0.9986	3	4	25	0	8	Beta lactam Genes	1
**PAML31.1 + Meropenem**	OXA-396	NA	NA	NA	NA	NA	0	0	25	0	8	Beta lactam Genes	NA
**PAML31.1 + Meropenem**	OXA-4	NA	NA	NA	NA	NA	1	5	25	0	8	Beta lactam Genes	NA
**PAML31.1 + Meropenem**	OXA-486	1.6	0.57	0	190487	0.9308	2	5	25	1	8	Beta lactam Genes	1
**PAML31.1 + Meropenem**	OXA-488	0.16	0	0	0.7	0.0455	2	1	25	2	8	Beta lactam Genes	0.7744
**PAML31.1 + Meropenem**	OXA-494	0	NA	NA	NA	NA	1	0	25	2	8	Beta lactam Genes	NA

bioRxiv preprint doi: https://doi.org/10.64898/2026.09.27.754834; this version posted September 29, 2026. The copyright holder for this preprint (which was not certified by peer review) is the author/funder, who has granted bioRxiv a license to display the preprint in perpetuity. It is made available under a CC-BY 4.0 International license.

**PAML31.1 + Meropenem**	OXA-50	0.16	0	0	0.03	0.0141	3	1	25	2	8	Beta lactam Genes	0.7286
**PAML31.1 + Meropenem**	OXA-846	NA	14334975214	0	Inf	0.9888	3	13	25	0	8	Beta lactam Genes	1
**PAML31.1 + Meropenem**	OXA-848	0	NA	NA	NA	NA	1	0	25	1	8	Beta lactam Genes	NA
**PAML31.1 + Meropenem**	OXA-9	NA	NA	NA	NA	NA	1	1	25	0	8	Beta lactam Genes	NA
**PAML31.1 + Meropenem**	OXA-914	NA	NA	NA	NA	NA	0	1	25	0	8	Beta lactam Genes	NA
**PAML31.1 + Meropenem**	OXA-937	NA	NA	NA	NA	NA	0	0	25	0	8	Beta lactam Genes	NA
**PAML31.1 + Meropenem**	PAO	0	NA	NA	NA	NA	1	0	25	1	8	Beta lactam Genes	NA
**PAML31.1 + Meropenem**	PDC-103	NA	NA	NA	NA	NA	1	1	25	0	8	Beta lactam Genes	NA
**PAML31.1 + Meropenem**	PDC-11	NA	NA	NA	NA	NA	1	3	25	0	8	Beta lactam Genes	NA
**PAML31.1 + Meropenem**	PDC-15	0	NA	NA	NA	NA	1	0	25	2	8	Beta lactam Genes	NA
**PAML31.1 + Meropenem**	PDC-16	0	NA	NA	NA	NA	1	0	25	1	8	Beta lactam Genes	NA
**PAML31.1 + Meropenem**	PDC-173	0	NA	NA	NA	NA	1	0	25	1	8	Beta lactam Genes	NA
**PAML31.1 + Meropenem**	PDC-19A	NA	328E+10	0	Inf	0.9811	2	10	25	0	8	Beta lactam Genes	1
**PAML31.1 + Meropenem**	PDC-3	1.92	2.46E+15	10.6	5.7E+29	0.0358	3	6	25	1	8	Beta lactam Genes	0.7744
**PAML31.1 + Meropenem**	PDC-34	NA	NA	NA	NA	NA	0	0	25	0	8	Beta lactam Genes	NA
**PAML31.1 + Meropenem**	PDC-35	0.32	NA	NA	NA	NA	1	1	25	1	8	Beta lactam Genes	NA
**PAML31.1 + Meropenem**	PDC-36	NA	NA	NA	NA	NA	1	1	25	0	8	Beta lactam Genes	NA
**PAML31.1 + Meropenem**	PDC-37	0	NA	NA	NA	NA	1	0	25	1	8	Beta lactam Genes	NA
**PAML31.1 + Meropenem**	PDC-407	NA	NA	NA	NA	NA	0	0	25	0	8	Beta lactam Genes	NA
**PAML31.1 + Meropenem**	PDC-5	NA	NA	NA	NA	NA	1	3	25	0	8	Beta lactam Genes	NA
**PAML31.1 + Meropenem**	PDC-8	NA	NA	NA	NA	NA	0	0	25	0	8	Beta lactam Genes	NA
**PAML31.1 + Meropenem**	PME-1	NA	NA	NA	NA	NA	1	2	25	0	8	Beta lactam Genes	NA
**PAML31.1 + Meropenem**	qacEdelta1	3.2	12.98	0.01	11843.57	0.4611	3	10	25	1	8	Efflux pumps Other Genes	1
**PAML31.1 + Meropenem**	qacG2	0	NA	NA	NA	NA	1	0	25	1	8	Efflux pumps Other Genes	NA
**PAML31.1 + Meropenem**	rmtD2	NA	NA	NA	NA	NA	1	2	25	0	8	Aminoglycoside Genes	NA
**PAML31.1 + Meropenem**	strA	NA	NA	NA	NA	NA	1	1	25	0	8	Aminoglycoside Genes	NA
**PAML31.1 + Meropenem**	sul1	1.6	15.85	0.03	9232.98	0.395	10	20	25	4	8	Sulfonamides Genes	1
**PAML31.1 + Meropenem**	tet(C)	NA	NA	NA	NA	NA	0	0	25	0	8	Tetracyclines Genes	NA
**PAML31.1 + Meropenem**	tet(G)	NA	7.53E+9	0	Inf	0.9911	3	8	25	0	8	Tetracyclines Genes	1
**PAML31.1 + Meropenem**	tet(R)	NA	NA	NA	NA	NA	1	3	25	0	8	Tetracyclines Genes	NA
**PAML31.1 + Meropenem**	-VEB 9.00	NA	NA	NA	NA	NA	1	3	25	0	8	Beta lactam Genes	NA
**PAML31.1 + Meropenem**	VIM-11	NA	NA	NA	NA	NA	1	1	25	0	8	Beta lactam Genes	NA
**PAML31.1 + Meropenem**	VIM-2	1.92	3.64E+16	13303	9.9E+28	0.0091	3	6	25	1	8	Beta lactam Genes	0.7286
**PAML31.1 + Tobramycin**	aac(3)-Id	NA	NA	NA	NA	NA	1	7	31	0	6	Aminoglycoside Genes	NA
**PAML31.1 + Tobramycin**	aac(6')-29B	0	NA	NA	NA	NA	1	0	31	1	6	Aminoglycoside Genes	NA
**PAML31.1 + Tobramycin**	aac(6')-33	1.94	1.49	0.04	58.8	0.8301	3	10	31	1	6	Aminoglycoside Genes	1

bioRxiv preprint doi: https://doi.org/10.64898/2026.09.27.754834; this version posted September 29, 2026. The copyright holder for this preprint (which was not certified by peer review) is the author/funder, who has granted bioRxiv a license to display the preprint in perpetuity. It is made available under a CC-BY 4.0 International license.

**PAML31.1 + Tobramycin**	aac(6')-Ib-G	NA	110656538.58	0	Inf	0.9977	3	9	31	0	6	Aminoglycoside Genes	1
**PAML31.1 + Tobramycin**	aac(6')-IB3	NA	NA	NA	NA	NA	1	1	31	0	6	Aminoglycoside Genes	NA
**PAML31.1 + Tobramycin**	aac(6')-IIa	NA	NA	NA	NA	NA	1	1	31	0	6	Aminoglycoside Genes	NA
**PAML31.1 + Tobramycin**	aac(6')-IIc	NA	61988783.24	0	Inf	0.9901	2	2	31	0	6	Aminoglycoside Genes	1
**PAML31.1 + Tobramycin**	aac(6')-Il	NA	2661430851.21	0	Inf	0.9981	3	11	31	0	6	Aminoglycoside Genes	1
**PAML31.1 + Tobramycin**	aac(6')-SET	NA	NA	NA	NA	NA	1	1	31	0	6	Aminoglycoside Genes	NA
**PAML31.1 + Tobramycin**	aadA1	2.52	6.34	0.28	140.94	0.2433	4	13	31	1	6	Aminoglycoside Genes	1
**PAML31.1 + Tobramycin**	aadA2	NA	NA	NA	NA	NA	1	7	31	0	6	Aminoglycoside Genes	NA
**PAML31.1 + Tobramycin**	aadA6	0.29	0.12	0	5.82	0.2872	2	3	31	2	6	Aminoglycoside Genes	1
**PAML31.1 + Tobramycin**	aadA7	NA	NA	NA	NA	NA	1	1	31	0	6	Aminoglycoside Genes	NA
**PAML31.1 + Tobramycin**	ant(2'')-la	0.97	1.64	0.04	72.78	0.7973	4	5	31	1	6	Aminoglycoside Genes	1
**PAML31.1 + Tobramycin**	aph(3')-Ia	NA	NA	NA	NA	NA	1	1	31	0	6	Aminoglycoside Genes	NA
**PAML31.1 + Tobramycin**	aph(3')-Ib	NA	NA	NA	NA	NA	1	1	31	0	6	Aminoglycoside Genes	NA
**PAML31.1 + Tobramycin**	aph(3')-IIb	1.12	2.41	0.09	65.33	0.6012	13	29	31	5	6	Aminoglycoside Genes	1
**PAML31.1 + Tobramycin**	aph(6)-Id	NA	47878265	0	Inf	0.9931	2	3	31	0	6	Aminoglycoside Genes	1
**PAML31.1 + Tobramycin**	bcr1	0.97	0	0	Inf	0.9977	13	30	31	6	6	Phenicols Bicyclomycins Genes	1
**PAML31.1 + Tobramycin**	catB7	0.26	0.01	0	4.85	0.1412	5	4	31	3	6	Phenicols Bicyclomycins Genes	0.9381
**PAML31.1 + Tobramycin**	cmlA5	NA	NA	NA	NA	NA	1	1	31	0	6	Phenicols Bicyclomycins Genes	NA
**PAML31.1 + Tobramycin**	cmlA6	NA	NA	NA	NA	NA	1	7	31	0	6	Phenicols Bicyclomycins Genes	NA
**PAML31.1 + Tobramycin**	cmlB	NA	NA	NA	NA	NA	1	1	31	0	6	Phenicols Bicyclomycins Genes	NA
**PAML31.1 + Tobramycin**	crpP	NA	10003741.85	0	Inf	0.9846	2	2	31	0	6	Quinolone Genes	1
**PAML31.1 + Tobramycin**	dfrB2	NA	NA	NA	NA	NA	1	3	31	0	6	Trimethoprim Genes	NA
**PAML31.1 + Tobramycin**	dfrB5	NA	3815944802.76	0	Inf	0.9983	2	8	31	0	6	Trimethoprim Genes	1
**PAML31.1 + Tobramycin**	floR2	NA	3114389559.07	0	Inf	0.9982	3	10	31	0	6	Phenicols Bicyclomycins Genes	1
**PAML31.1 + Tobramycin**	fosA	1.16	4.52	0.06	338.24	0.493	12	30	31	5	6	Fosfomycin Genes	1
**PAML31.1 + Tobramycin**	GES-1	NA	NA	NA	NA	NA	1	1	31	0	6	Beta lactam Genes	NA
**PAML31.1 + Tobramycin**	GES-19	NA	223874539.6	0	Inf	0.9974	2	9	31	0	6	Beta lactam Genes	1
**PAML31.1 + Tobramycin**	GES-20	1.55	0.92	0.03	27.5	0.9623	3	8	31	1	6	Beta lactam Genes	1
**PAML31.1 + Tobramycin**	GES-26	0.39	0.16	0	6.56	0.3298	2	2	31	1	6	Beta lactam Genes	1
**PAML31.1 + Tobramycin**	IMP-1	NA	NA	NA	NA	NA	1	1	31	0	6	Beta lactam Genes	NA
**PAML31.1 + Tobramycin**	KPC-5	NA	NA	NA	NA	NA	1	1	31	0	6	Beta lactam Genes	NA
**PAML31.1 + Tobramycin**	mexA	1	NA	NA	NA	NA	14	31	31	6	6	Efflux pumps Other Genes	NA
**PAML31.1 + Tobramycin**	mexE	1	NA	NA	NA	NA	14	31	31	6	6	Efflux pumps Other Genes	NA
**PAML31.1 + Tobramycin**	mexX	1.06	0.65	0.03	13.31	0.7785	9	22	31	4	6	Efflux pumps Other Genes	1
**PAML31.1 + Tobramycin**	NDM-1	NA	NA	NA	NA	NA	1	2	31	0	6	Beta lactam Genes	NA
**PAML31.1 + Tobramycin**	OXA-10	NA	42864153.82	0	Inf	0.9981	2	4	31	0	6	Beta lactam Genes	1
**PAML31.1 + Tobramycin**	OXA-101	NA	NA	NA	NA	NA	1	1	31	0	6	Beta lactam Genes	NA

bioRxiv preprint doi: https://doi.org/10.64898/2026.09.27.754834; this version posted September 29, 2026. The copyright holder for this preprint (which was not certified by peer review) is the author/funder, who has granted bioRxiv a license to display the preprint in perpetuity. It is made available under a CC-BY 4.0 International license.

**PAML31.1 + Tobramycin**	OXA-2	2.13	3.79	0.17	82.67	0.3968	4	11	31	1	6	Beta lactam Genes	1
**PAML31.1 + Tobramycin**	OXA-395	NA	5.574E+12	0	Inf	0.9965	3	5	31	0	6	Beta lactam Genes	1
**PAML31.1 + Tobramycin**	OXA-396	NA	NA	NA	NA	NA	0	0	31	0	6	Beta lactam Genes	NA
**PAML31.1 + Tobramycin**	OXA-4	NA	NA	NA	NA	NA	1	7	31	0	6	Beta lactam Genes	NA
**PAML31.1 + Tobramycin**	OXA-486	NA	NA	NA	NA	NA	1	7	31	0	6	Beta lactam Genes	NA
**PAML31.1 + Tobramycin**	OXA-488	0.39	0.29	0.02	4.44	0.372	2	4	31	2	6	Beta lactam Genes	1
**PAML31.1 + Tobramycin**	OXA-494	0	NA	NA	NA	NA	1	0	31	1	6	Beta lactam Genes	NA
**PAML31.1 + Tobramycin**	OXA-50	0.1	0.06	0	2	0.1174	3	1	31	2	6	Beta lactam Genes	0.8608
**PAML31.1 + Tobramycin**	OXA-846	NA	1743886427.15	0	Inf	0.998	3	13	31	0	6	Beta lactam Genes	1
**PAML31.1 + Tobramycin**	OXA-848	0.19	NA	NA	NA	NA	1	1	31	1	6	Beta lactam Genes	NA
**PAML31.1 + Tobramycin**	OXA-9	NA	NA	NA	NA	NA	1	1	31	0	6	Beta lactam Genes	NA
**PAML31.1 + Tobramycin**	OXA-914	NA	NA	NA	NA	NA	0	0	31	0	6	Beta lactam Genes	NA
**PAML31.1 + Tobramycin**	OXA-937	NA	NA	NA	NA	NA	0	0	31	0	6	Beta lactam Genes	NA
**PAML31.1 + Tobramycin**	PAO	0	NA	NA	NA	NA	1	0	31	1	6	Beta lactam Genes	NA
**PAML31.1 + Tobramycin**	PDC-103	NA	NA	NA	NA	NA	1	1	31	0	6	Beta lactam Genes	NA
**PAML31.1 + Tobramycin**	PDC-11	NA	NA	NA	NA	NA	1	3	31	0	6	Beta lactam Genes	NA
**PAML31.1 + Tobramycin**	PDC-15	0	NA	NA	NA	NA	1	0	31	1	6	Beta lactam Genes	NA
**PAML31.1 + Tobramycin**	PDC-16	0.19	NA	NA	NA	NA	1	1	31	1	6	Beta lactam Genes	NA
**PAML31.1 + Tobramycin**	PDC-173	0	NA	NA	NA	NA	1	0	31	1	6	Beta lactam Genes	NA
**PAML31.1 + Tobramycin**	PDC-19A	NA	461486056.33	0	Inf	0.9972	2	10	31	0	6	Beta lactam Genes	1
**PAML31.1 + Tobramycin**	PDC-3	NA	4675971657.89	0	Inf	0.9984	2	8	31	0	6	Beta lactam Genes	1
**PAML31.1 + Tobramycin**	PDC-34	NA	NA	NA	NA	NA	0	0	31	0	6	Beta lactam Genes	NA
**PAML31.1 + Tobramycin**	PDC-35	0.58	NA	NA	NA	NA	1	3	31	1	6	Beta lactam Genes	NA
**PAML31.1 + Tobramycin**	PDC-36	NA	NA	NA	NA	NA	1	1	31	0	6	Beta lactam Genes	NA
**PAML31.1 + Tobramycin**	PDC-37	0.19	NA	NA	NA	NA	1	1	31	1	6	Beta lactam Genes	NA
**PAML31.1 + Tobramycin**	PDC-407	NA	NA	NA	NA	NA	0	0	31	0	6	Beta lactam Genes	NA
**PAML31.1 + Tobramycin**	PDC-5	NA	NA	NA	NA	NA	1	3	31	0	6	Beta lactam Genes	NA
**PAML31.1 + Tobramycin**	PDC-8	NA	NA	NA	NA	NA	0	0	31	0	6	Beta lactam Genes	NA
**PAML31.1 + Tobramycin**	PME-1	NA	NA	NA	NA	NA	1	2	31	0	6	Beta lactam Genes	NA
**PAML31.1 + Tobramycin**	qacEdelta1	1.94	1.49	0.04	58.8	0.8301	3	10	31	1	6	Efflux pumps Other Genes	1
**PAML31.1 + Tobramycin**	qacG2	0	NA	NA	NA	NA	1	0	31	1	6	Efflux pumps Other Genes	NA
**PAML31.1 + Tobramycin**	rmtD2	NA	NA	NA	NA	NA	1	2	31	0	6	Aminoglycoside Genes	NA
**PAML31.1 + Tobramycin**	strA	NA	47878265	0	Inf	0.9931	2	3	31	0	6	Aminoglycoside Genes	1
**PAML31.1 + Tobramycin**	sul1	1.16	0.71	0.04	14.42	0.8266	10	24	31	4	6	Sulfonamides Genes	1
**PAML31.1 + Tobramycin**	tet(C)	NA	NA	NA	NA	NA	1	2	31	0	6	Tetracyclines Genes	NA
**PAML31.1 + Tobramycin**	tet(G)	NA	3114389559.07	0	Inf	0.9982	3	10	31	0	6	Tetracyclines Genes	1
**PAML31.1 + Tobramycin**	tet(R)	NA	NA	NA	NA	NA	1	3	31	0	6	Tetracyclines Genes	NA
**PAML31.1 + Tobramycin**	-VEB 9.00	NA	NA	NA	NA	NA	1	3	31	0	6	Beta lactam Genes	NA

bioRxiv preprint doi: https://doi.org/10.64898/2026.09.27.754834; this version posted September 29, 2026. The copyright holder for this preprint (which was not certified by peer review) is the author/funder, who has granted bioRxiv a license to display the preprint in perpetuity. It is made available under a CC-BY 4.0 International license.

**PAML31.1 + Tobramycin**	VIM-11	NA	NA	NA	NA	NA	1	1	31	0	6	Beta lactam Genes	NA
**PAML31.1 + Tobramycin**	VIM-2	1.55	1.09	0.03	34.61	0.9603	3	8	31	1	6	Beta lactam Genes	1

Supplemental Table 9 Analysis of gene enrichment/depletion in phage-resistant groups of strains among antibiotic-resistant groups of strains, as reported by a single mixed model adjusting for phylogenetic position and clonal lineage. Pairing, the particular phage and antibiotic being examined; Gene, the resistance gene being examined for presence/absence in various groups of strains; FC, fold change in phage-resistant group compared to phage-susceptible group; Odds Ratio, the ratio of the odds of being phage-resistant versus phage-susceptible within the antibiotic-resistant group of strains after controlling for phylogenetic position and clonal lineage; CIL, 95% Wald Confidence interval lower bound; CIU: 95% Wald Confidence interval upper bound; Pval: unadjusted p value; LW, total number of lineages with the gene; PRW, number of phage resistant strains that carry the gene (out of the antibiotic resistant strains); PR, number of phage resistant strains total (out of the antibiotic resistant strains); PSW, number of phage susceptible strains that carry the gene (out of the antibiotic resistant strains); PS, number of phage susceptible strains total (out of the antibiotic resistant strains); Category, refers to type of resistance conferred by a particular gene; FDR, FDR-adjusted P value. If only 1 lineage carries the gene, statistical analysis throws NA because the gene effect is perfectly confounded by the lineage effect.
